# Synthesis of Small‐Molecule Fluorescent Probes for the In Vitro Imaging of Calcium‐Activated Potassium Channel K_Ca_3.1

**DOI:** 10.1002/anie.202001201

**Published:** 2020-03-17

**Authors:** Kathrin Brömmel, Sarah Maskri, Ivan Maisuls, Christian Paul Konken, Marius Rieke, Zoltan Pethő, Cristian A. Strassert, Oliver Koch, Albrecht Schwab, Bernhard Wünsch

**Affiliations:** ^1^ Institute for Pharmaceutical and Medicinal Chemistry Westphalian Wilhelms-University Münster Corrensstraße 48 48149 Münster Germany; ^2^ Center for Nanotechnology Center for Soft Nanoscience Institute for Inorganic and Analytical Chemistry Westphalian Wilhelms-University Münster Heisenbergstraße 11 48149 Münster Germany; ^3^ Department of Nuclear Medicine University Hospital Münster Albert-Schweitzer-Campus 1, Building A1 48149 Münster Germany; ^4^ Institute for Physiology II University Hospital Münster Robert-Koch-Straße 27b 48149 Münster Germany; ^5^ Cells-in-Motion Interfaculty Center Westphalian Wilhelms-University Münster Waldeyerstraße 15 84149 Münster Germany

**Keywords:** cycloaddition, fluorescent probes, K_Ca_3.1 channels, molecular modelling, non-small cell lung cancer

## Abstract

Small‐molecule probes for the in vitro imaging of K_Ca_3.1 channel‐expressing cells were developed. Senicapoc, showing high affinity and selectivity for the K_Ca_3.1 channels, was chosen as the targeting component. BODIPY dyes **15**–**20** were synthesized and connected by a Cu^I^‐catalyzed azide–alkyne [3+2]cycloaddition with propargyl ether senicapoc derivative **8**, yielding fluorescently labeled ligands **21**–**26**. The dimethylpyrrole‐based imaging probes **25** and **26** allow staining of K_Ca_3.1 channels in NSCLC cells. The specificity was shown by removing the punctate staining pattern by pre‐incubation with senicapoc. The density of K_Ca_3.1 channels detected with **25** and by immunostaining was identical. The punctate structure of the labeled channels could also be observed in living cells. Molecular modeling showed binding of the senicapoc‐targeting component towards the binding site within the ion channel and orientation of the linker with the dye along the inner surface of the ion channel.

## Graphical abstract

**Imaging channels**: Small‐molecule probes for the in vitro imaging of K_Ca_3.1 channel‐expressing cells were designed and developed. Using senicapoc as the targeting component, the BODIPY‐labeled probes could selectively stain the channels in living cells and the channel punctate structure could be observed.

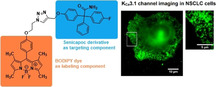

## Introduction

Ion channels play a critical role in the progression of cancer and contribute to features of essentially all “cancer hallmarks”.^1^ The calcium‐activated potassium channel 3.1 (K_Ca_3.1) is an intensively studied ion channel in this context. It is involved in critical steps of the metastatic cascade, such as proliferation, migration, invasion, and extravasation.^2^, ^3^ Inhibition of the K_Ca_3.1 channel leads in many different tumor entities to reduced proliferation, migration, and metastasis.^4^, ^5^, ^6^ Overexpression of this channel directly correlates with tumor grade and metastatic status and is often related to poor prognosis for tumor patients and high lethality rates.^7^ Moreover, expression of K_Ca_3.1 channels is dysregulated in many tumor entities. When patients are stratified according to K_Ca_3.1 channel expression, patient survival is worse for those with elevated K_Ca_3.1 expression. This points to the predictive power of analyzing K_Ca_3.1 channel expression with respect to patient survival.^8^, ^9^

Therefore, developing tools for the visualization of K_Ca_3.1‐expressing cells would be a great asset for a better understanding of its (diagnostic) role and predictive value in cancer. Currently, only indirect immunoflourescence staining is available for optical imaging of the K_Ca_3.1 channel, making the process time‐consuming and expensive. Herein, we present the development and synthesis of novel small‐molecule probes, which show promising results in in vitro imaging of the K_Ca_3.1‐expressing cells by fluorescence microscopy. Simple, fast, and efficient staining protocols are the advantages of these novel imaging probes, which can be instrumental for elucidating the mechanisms by which K_Ca_3.1 channels contribute to cancer progression and metastasis.

## Results and Discussion

### Synthesis of the Probes

**Figure 1:**
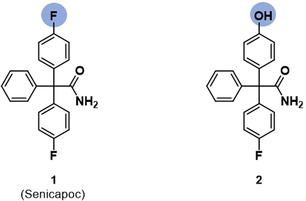
Chemical structure of senicapoc (**1**) and its derivative **2**.

For the development of small‐molecule fluorescently labeled probes of the K_Ca_3.1 channel, senicapoc (**1**) served as starting point. Senicapoc (**1**) has a high affinity towards the K_Ca_3.1 channel in the low‐nanomolar range (IC_50_=11±2 nm; measured on human erythrocytes) and excellent selectivity over related ion channels.^10^ In order to introduce a fluorescent dye one F‐atom of senicapoc should be replaced by a hydroxy moiety. The OH moiety of the senicapoc derivative **2** should allow broad chemical modifications including the introduction of a fluorescent dye (Figure 1).

**Scheme 1:**
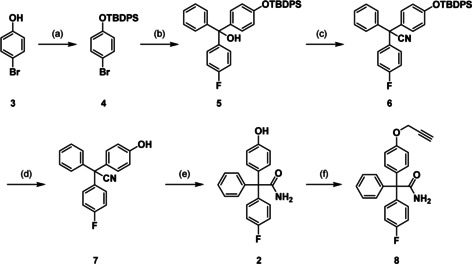
Synthesis of the senicapoc derivative **8**. Reagents and reaction conditions: a) imidazole, TBDPS‐Cl, DMF, 24 h, rt, 96 %. b) 1. *n*‐ butyllithium, THF, 1 h, −78 °C; 2. 4‐fluorobenzophenone, THF, 3 h, rt, 58 %. c) 1. InCl_3_, trimethylsilyl cyanide, CH_2_Cl_2,_ 1 h, 20–40 °C, 1.5 h, 40–50 °C; 2. KOH, KNa‐tartrate, H_2_O, 12 h, 0 °C—rt, 89 %. d) KOH, KNa‐tartrate, 2‐methylbutan‐2‐ol,5 h, 100 °C, 88 %. e) KOH, EtOH, H_2_O, 90 °C, 48 h, 23 %, f) propargyl bromide, Cs_2_Co_3_, DMF, 30 min, rt, 90 %.

The phenol **2** was obtained in five steps starting with the protection of 4‐bromophenol (**3**) with *tert*‐butyldiphenylsilyl chloride (TBDPS‐Cl). The protected 4‐bromophenol **4** was reacted with *n*‐butyllithium in THF to obtain the lithiated intermediate, which was trapped with 4‐fluorobenzophenone yielding the tertiary alcohol **5**. Trimethylsilyl cyanide and catalytic amounts of InCl_3_ were used to convert the alcohol **5** into the nitrile **6**. The silyl protective group was cleaved under basic conditions resulting in the intermediate **7** with a free hydroxy group in *p*‐position. In the last step, the nitrile was hydrolyzed partially under basic conditions to obtain the senicapoc derivative **2**. Although the partial hydrolysis of the nitrile to an amide has been described for the synthesis of senicapoc in high yields, in case of the phenol **7** the transformation was rather low and incomplete resulting in a yield of the amide **2** of 23 %. However, a large amount of the starting material **7** could be re‐isolated, which increased the efficiency of the last reaction step considerably (Scheme 1).

For the introduction of the fluorescent label a Cu^I^‐catalyzed azide–alkyne [3+2]cycloaddition was envisaged. Therefore, an alkyne moiety had to be introduced into the phenol **2**. For this purpose, phenol **2** was alkylated with propargyl bromide in the presence of Cs_2_CO_3_ to afford the propargyl ether **8** (Scheme 1).

In order to visualize the senicapoc‐derived targeting component by the introduction of a fluorescent dye, different boron dipyrromethene (BODIPY) dyes were designed bearing an azide moiety suitable for the planned [3+2]cycloaddition reaction with the alkyne **8**.

The photochemical properties of the BODIPY dyes are easily adjustable by variation of the substitution pattern of the dipyrromethene core.^11^, ^12^ In terms of medicinal research, the BODIPY dyes show some important advantages; namely, the emission intensity derived from high quantum yields is almost independent from the pH value and the polarity of the solvent. Moreover, low toxicity was reported. On the other hand, low solubility of some dyes in water and physiological water‐based buffer systems may cause problems concerning their application in biological systems.

**Scheme 2:**
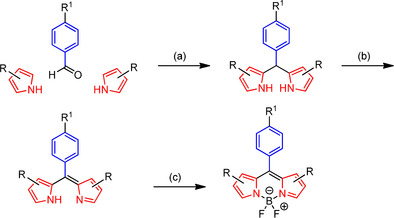
General concept for the synthesis of BODIPY dyes. a) H^+^. b) oxidant, for example, *p*‐chloranil. c) NEt_3_, BF_3_⋅OEt_2_. (modified according to literature^13^).

For the synthesis of BODIPY‐based dyes, a general one‐pot three‐step approach was pursued, which includes the acid catalyzed condensation of two equivalents of a pyrrole with one equivalent of a benzaldehyde followed by oxidation of the dipyrrolylmethane with 2,3,5,6‐tetrachloro‐1,4‐benzoquinone (*p*‐chloranil). Then, the oxidized product was deprotonated with a base and complexed with BF_3_⋅OEt_2_ to give the BODIPY dye^13^ (Scheme 2).

Due to the limited conjugation with the dipyrrolomethyne core, the phenyl moiety has only minor effects on the excitation and emission wavelength of the resulting BODIPY dye. Herein, benzaldehyde derivatives were used bearing an azide moiety within the substituent in *p*‐position. This azido moiety should be used for the connection of the BODIPY dye with the targeting component through a Cu^I^‐catalyzed azide–alkyne [3+2]cycloaddition. Consequently, the benzaldehyde derivatives contain the linker to the targeting component. Benzaldehyde derivatives with different lengths and polarity of the linker, that is, an ethoxy‐ (**9**), a 3,6,9‐trioxaundecyloxy (**10**) and a dodecyloxy linker (**11**) between the phenyl ring and the azido moiety were synthesized as described in literature^13^, ^14^, ^15^ and further used for the synthesis of the BODIPY dyes.

**Scheme 3:**
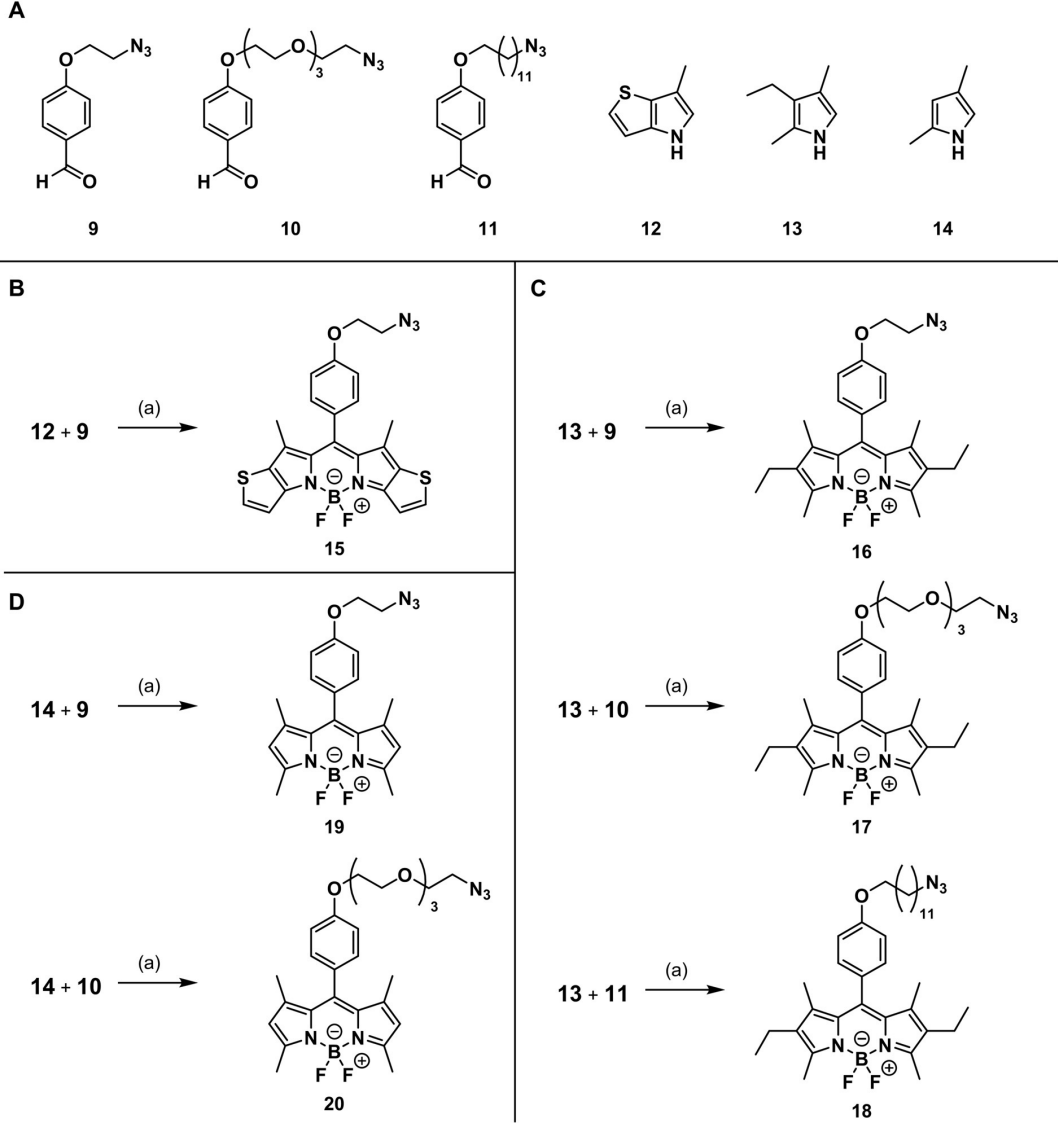
A) Structures of benzaldehyde derivatives **9**–**11** and pyrrole derivatives **12**–**14** used for the BODIPY synthesis. B–D) Synthesis of BODIPY dyes with azide moiety in the side chain. Series (B) is based on the thiophene annulated pyrrole **12**, series (C) is based on the ethyldimethylpyrrole **13** and series (D) on the dimethylpyrrole **14**. Reagents and reaction conditions: a) 1. TFA, CH_2_Cl_2_, molecular sieves 3 Å, rt, 24 h; 2. *p*‐chloranil, CH_2_Cl_2_, rt, 30 min; 3. NEt_3_, CH_2_Cl_2_, rt, 15 min; 4. BF_3_⋅OEt_2_, CH_2_Cl_2_, rt, 12 h. Yields: **15**: 7 %. **16**: 14 %. **17**: 30 %. **18**: 22 %. **19**: 16 %. **20**: 27 %. Yields were calculated over three reaction steps for >90 % purity compounds.

The dipyrromethene core, which defines the optical properties of the dye, can be varied by starting from different pyrrole derivatives. An enlarged conjugated π‐electron system resulting from annulation of an aromatic ring at the pyrrole ring shifts the emission wavelength towards the near‐infrared region. To achieve this effect, the annulated thienopyrrole **12** was reacted with benzaldehyde **9** according to the general one‐pot procedure outlined in Scheme 3 to obtain BODIPY dye **15**. Due to stability issues with **15**, containing the thienopyrrole system, alkyl substituents were taken into consideration. In general, most of the reactive pyrrole positions should be blocked by suitable substituents to avoid side reactions during the condensation with benzaldehydes and to enhance the stability of the final dye. Therefore, 3‐ethyl‐2,4‐dimethylpyrrole (**13**) was reacted with the benzaldehydes **9**–**11** to provide the stable and storable BODIPY dyes **16**–**18**. In order to reduce the size and lipophilicity of the dyes, the reaction of 2,4‐dimethylpyrrole (**14**) with benzaldehydes **9** and **10** was carried out to afford two additional stable and storable BODIPY dyes **19** and **20**.

Since fluorescence microscopy should be performed with the designed fluorescently labeled ligands, the synthesized dyes need to be suitable for this imaging technique. Due to lack of conjugation with the dipyrrolomethyne core, electronic effects were not expected regarding the photophysical properties in relation to the conjugation of the phenyl moiety, its substituents, or the targeting component. Therefore, the optical characteristics of the six BODIPY dyes **15**–**20** were analyzed before attachment of the targeting component. High quantum yields are desirable. Excitation and emission wavelengths suitable for commercially available filter sets (FITC, TRITC) are necessary. The photophysical properties of the BODIPY dyes **15**–**20** are summarized in Tables 1–3 and discussed thereafter (see below).

The wavelengths of the FITC and TRITC filter sets are as follows: *λ*_excitation_(FITC)=450–490 nm, *λ*_emission_(FITC)=515–565 nm; and *λ*_excitation_(TRITC)=546/12 nm (bandpass filter), *λ*_emission_(TRITC)=590 nm (longpass filter). Thus, the absorption and emission wavelengths of BODIPY dyes **16**–**20** fit to the FITC filter set. The TRITC filter set can be used for fluorescence microscopy of BODIPY **15**.

The solvatochromic shift in the absorption and in the emission maxima is modest for all dyes when increasing the polarity of the solvent, which points to a predominant ππ* character of the excited states that also accounts for the small Stokes shifts and the vibrational shoulders observed in all cases (see Table 1 and the corresponding spectra in the Supporting Information).

**Table 1:** Absorption and emission maxima of the dyes **15**–**20**.

compd.	*λ*_abs, max_ [nm]^[a]^	*λ*_em, max_ [nm]^[b]^	*λ*_abs, max_ [nm]^[c]^	*λ*_em, max_ [nm]^[d]^
**15**	553	571	559	574
**16**	521	533	524	537
**17**	521	533	524	537
**18**	521	532	524	535
**19**	497	507	500	510
**20**	497	508	500	510

[a]  Absorption in CH_3_CN. [b] Emission in CH_3_CN. [c] Absorption in CH_2_Cl_2_/CH_3_OH. [d] Emission in CH_2_Cl_2_/CH_3_OH.

However, the absorption and the emission spectra both in CH_3_CN and in the more polar CH_2_Cl_2_/CH_3_OH mixture clearly show a bathochromic shift for compound **15** and a blue‐shift for **19** and **20** compared with **16**–**18**, indicating that the energy of the excited states can be fine‐tuned by judicious choice of the substitution pattern. In particular, the fused thiophene rings extend the delocalization of the π system, causing the observed red‐shifted absorption and emission maxima. Interestingly, the fluorescence rate constants in CH_3_CN are comparable for all exemplars except for compound **15**, which is significantly smaller (Table 2).

**Table 2:** Photoluminescence quantum yields (*Φ*_F_), fluorescence lifetimes (*τ*_F_), fluorescence (*k*_F_) and non‐radiative (*k*_nr_) rate constants in fluid CH_3_CN at 298 K of compounds **15**–**20**.

compd.	*Φ*_F_	*τ*_F_ [ns]	*k*_F_ [×10^6^ s^−1^]	*k*_nr_ [×10^6^ s^−1^]
**15**	0.04	0.891±0.006	40±20	1077±30
**16**	0.81	6.32±0.05	128±4	30±5
**17**	0.81	6.37±0.05	127±4	30±5
**18**	0.82	6.60±0.06	124±4	27±6
**19**	0.53	3.96±0.03	134±6	120±8
**20**	0.50	4.09±0.03	122±6	122±8

[a] (±0.02)

Moreover, the radiationless deactivation rates are drastically higher for the latter and significantly enhanced for **19** and **20**, if compared with **16**–**18**. This is surprising since **16**–**18** have additional side chains providing extra degrees of freedom and therefore a higher density of rotovibrational states that could favor non‐radiative relaxation pathways. This points to radiationless transitions that are mostly governed by electronic effects associated to the variable substitution patterns. The latter is evident for **19**–**20** but particularly for **15**, as can be also observed in the more polar CH_2_Cl_2_/CH_3_OH mixture in which all cases the radiative relaxation rates are slightly slower than in CH_3_CN while the radiationless processes are somewhat faster than in the less polar environment (Table 3). Interestingly, in frozen matrices at 77 K, all dyes show practically identically high radiative rate constants, whereas the radiationless process is only (yet drastically) boosted for **15**. This observation points to an enhanced intersystem crossing that could be ascribed to the additional lone pairs from the sulfur atoms at the thiophene rings (El Syed rule). It is also evident that the substitution at the methyne bridge has no significant effect on the excited state energies and their relaxation rate constants.

**Table 3:** Photoluminescence quantum yields (*Φ*_F_), fluorescence lifetimes (*τ*_F_), fluorescence (*k*_F_) and non‐radiative (*k*_nr_) rate constants in CH_2_Cl_2_/CH_3_OH (1:1) of compounds **15**–**20**.

compd.	*Φ*_F_^[a], [b]^	*τ*_F_ [ns]^[b]^	*k*_F_ [×10^6^ s^−1^]^[b]^	*k*_nr_ [×10^6^ s^−1^]^[b]^	*Φ*_F_^[c], [d]^	*τ*_F_ [ns]^[d]^	*k*_F_ [×10^6^ s^−1^]^[d]^	*k*_nr_ [×10^6^ s^−1^]^[d]^
**15**	0.05	0.818±0.004	61±30	1160±40	0.1	1.252±0.002	<160	720±80
**16**	0.73	6.00±0.02	122±4	45±5	0.9	5.99±0.02	150±20	<30
**17**	0.63	6.54±0.03	96±4	57±5	0.9	5.46±0.02	160±20	<40
**18**	0.64	5.83±0.03	110±4	62±6	0.9	5.6±0.2	160±20	<40
**19**	0.41	3.810±0.016	108±6	154±8	0.9	5.06±0.02	180±20	<40
**20**	0.44	4.408±0.018	100±5	127±8	0.9	5.8±0.2	160±20	<30

[a] (±0.02). [b] 298 K. [c] (±0.1). [d] 77 K.

**Scheme 4:**
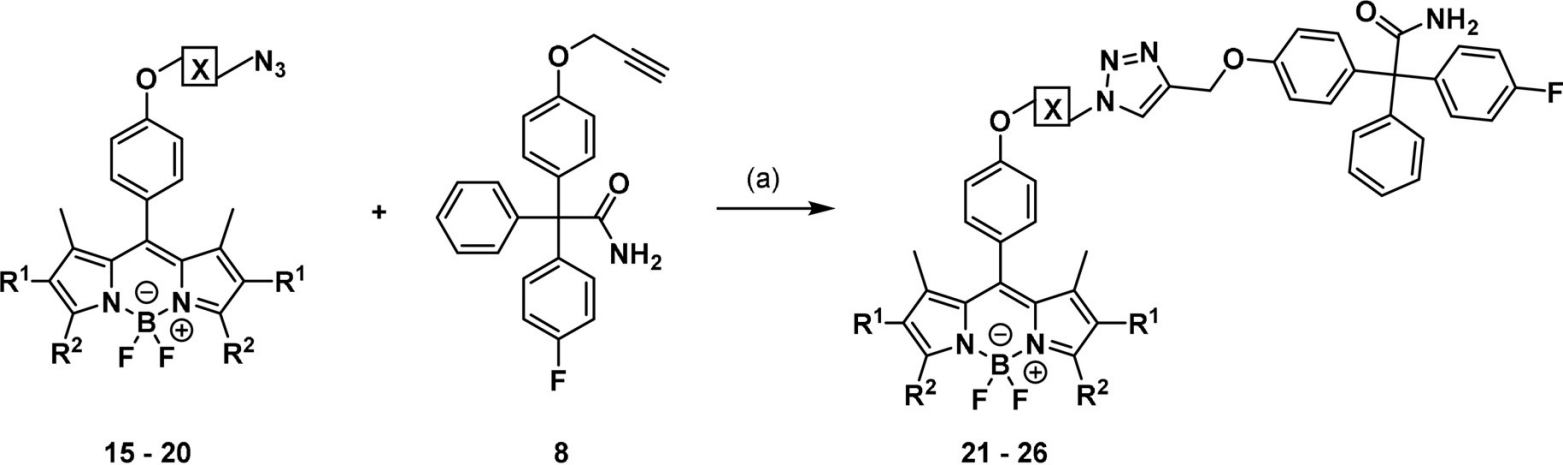
Synthesis of fluorescently labeled tool compounds. Reagents and reaction conditions: a) CuSO_4_, sodium ascorbate, DMF, H_2_O, rt, 24 h, **21**: 58 %, **22**: 57 %, **23**: 61 %, **24**: 2 %, **25**: 19 %, **26**: 68 %.

In order to obtain the desired fluorescently labeled ligands, the synthesized BODIPY dyes **15**–**20** were coupled with the alkyne **8** by a Cu^I^‐catalyzed azide–alkyne [3+2]cycloaddition reaction (Scheme 4).

**Figure 2:**
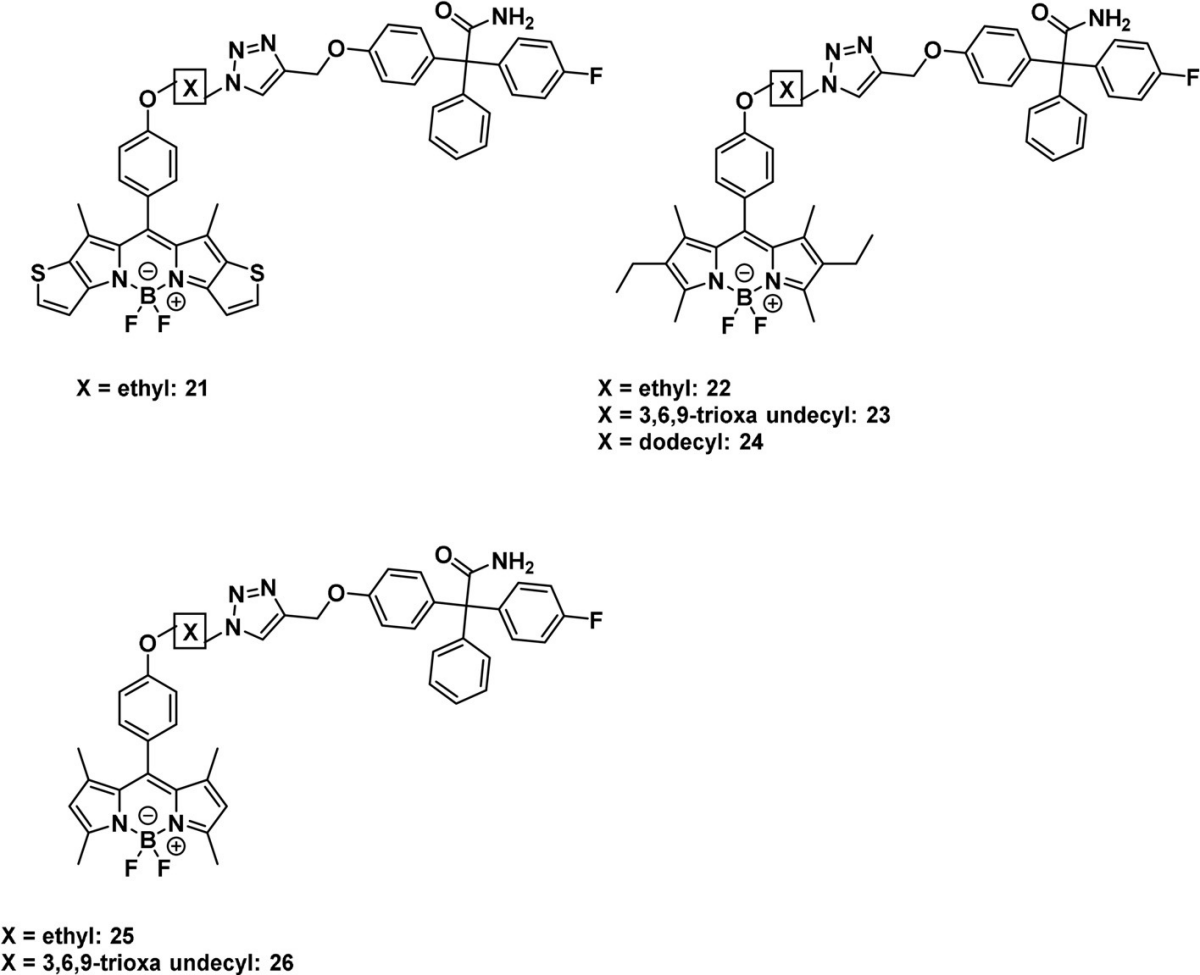
Fluorescently labeled ligands **21**–**26** designed to image K_Ca_3.1 channels.

The six fluorescently labeled ligands **21**–**26** shown in Figure 2 were tested in first simple staining experiments to analyze their suitability for in vitro imaging of the K_Ca_3.1 channel.

### In Vitro Imaging of NSCLC Cells

The A549 cell line, used for all experiments, is a hypotriploid epithelial cell line from a non‐small‐cell lung cancer (NSCLC) taken from a 58‐year‐old Caucasian in 1972. The suffix “3R” refers to the repeated process of intravenous administration of parental cells (also initially “0R”) into immunocompromised mice to obtain lung metastases. Cells from these metastases were isolated and cultured in vitro followed by another round of reinjection to select for tumor cells with high metastatic potential.^16^

**Figure 3:**
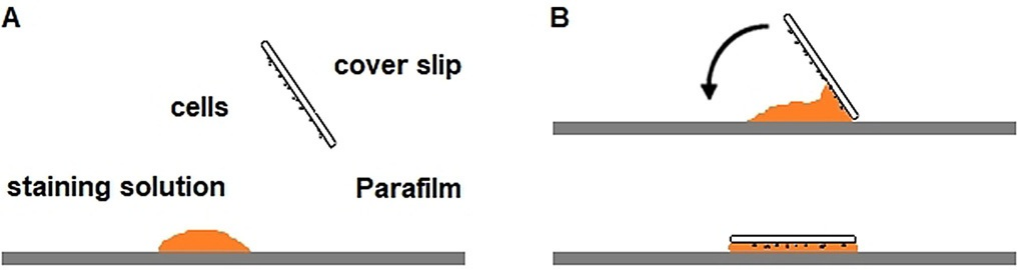
Setup for the staining of NSCLC cells.

A549‐3R cells overexpress the K_Ca_3.1 channel.^7^ Thus, they were selected for testing of the novel small‐molecule imaging probes. We aim at the development of a fast and simple staining protocol. Therefore, a drop (30 μL) of a previously prepared staining solution (10 μm in PBS) of the respective fluorescently labeled ligand was pipetted onto Parafilm. The cover slip with the adherent cells was placed carefully upside down onto the drop and the NSCLC cells were incubated in a humidified dark chamber for 10 min (Figure 3).

The NSCLC cells are ready for microscopy after only 10 min incubation time and subsequent washing with PBS‐buffer. This newly developed protocol using the fluorescently labeled probes is very fast and simple in comparison to the much more complicated indirect immunofluorescence assay (ca. 4 h). An inverted fluorescence microscope was used to image NSCLC cells labeled with the fluorescent senicapoc derivative.

After performing the staining protocol with the fluorescently labeled thiophene annulated ligand **21**, staining of the A549‐3R cells could not be observed. To ensure that this result is not due to the low quantum yield of BODIPY **15**, a drop of the staining solution of **21** was examined under the microscope and an orange‐red color was observed. Thus, imaging probe **21** is not suitable for the visualization of the K_Ca_3.1 channels.

The same staining protocol was performed with the fluorescently labeled ligands **22** and **23** bearing an ethyl and two methyl moieties at the pyrrole rings and the NSCLC cells were observed. Unfortunately, the staining was not specific and no staining pattern of the K_Ca_3.1 channels was found (see Figure S1 in the Supporting Information).

Problems occurred while preparing the staining solution of fluorescently labeled ligand **24** with the undecyloxy linker. Due to its high lipophilicity, it was not possible to dilute the DMSO stock solution with PBS‐buffer. The molecule precipitated immediately and neither ultrasound nor heating of the suspension led to a clear solution. Thus, it was not possible to perform the staining experiments with imaging probe **24**.

**Figure 4:**
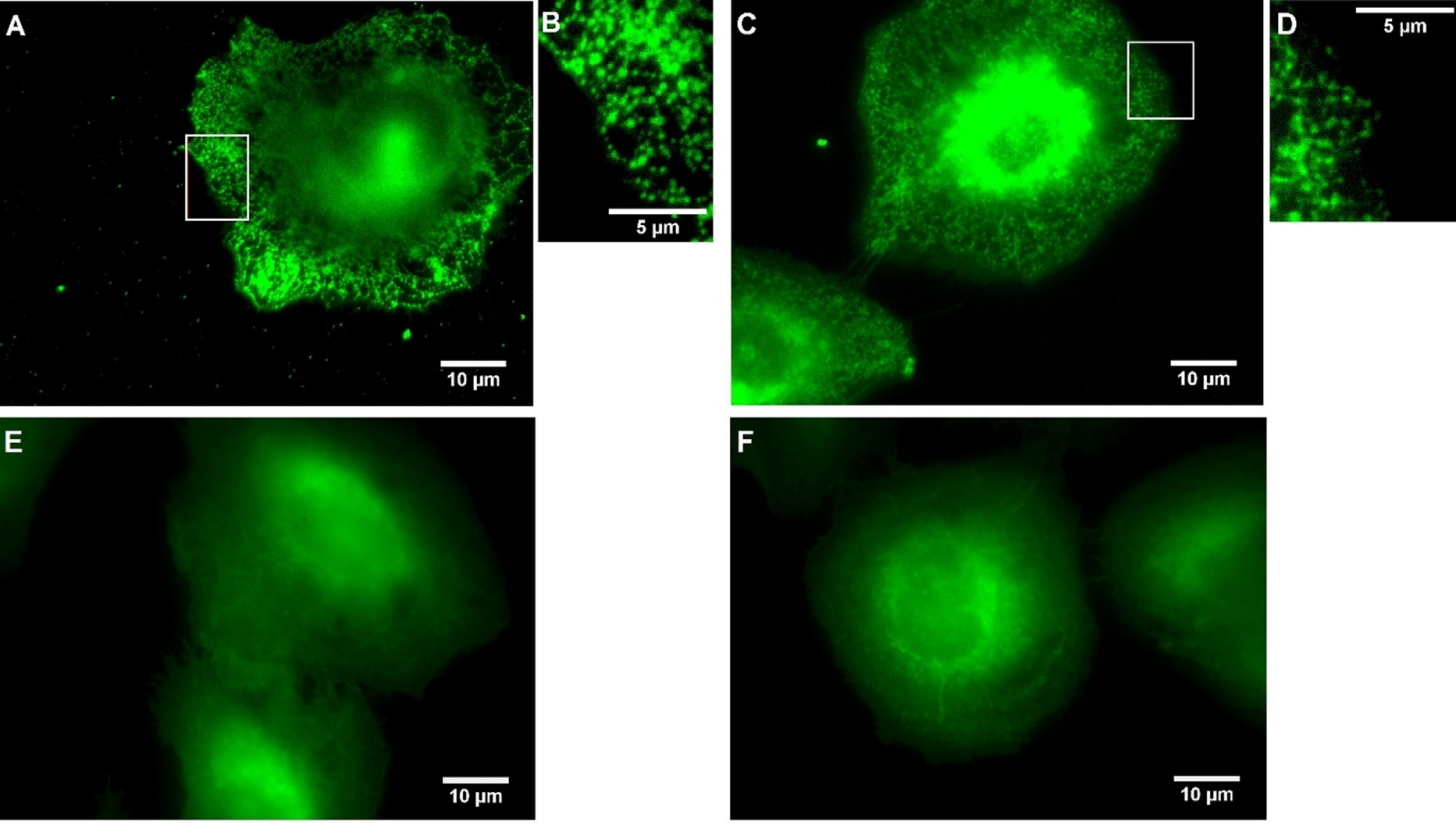
A) NSCLC cells incubated for 10 min with a 10 μm staining solution of imaging probe **25**. B) Magnification of (A) (white box). C) NSCLC cells incubated for 10 min with a 10 μm staining solution of imaging probe **26**. D) Magnification of (C) (white box). E) NSCLC cells blocked for 5 min with unlabeled senicapoc (30 μm) and subsequently stained for 10 min with a 10 μm staining solution of imaging probe **25**. F) NSCLC cells blocked for 5 min with unlabeled senicapoc (30 μm) and subsequently for 10 min with a 10 μm staining solution of imaging probe **26**.

In contrast, the fluorescently labeled dimethylpyrrole‐based ligands **25** and **26** showed promising results. In both cases the typical punctate staining pattern of the K_Ca_3.1 channel was observed^7^, ^17^ (Figure 4 A–D).

As a proof of principle, the NSCLC cells were pre‐incubated with unlabeled senicapoc (30 μm) 5 min before staining. Consequently, the typical punctate staining pattern could not be observed any more (Figure 4 E,F).

Since all the binding regions were blocked with senicapoc, the fluorescently labeled probes **25** and **26** were no longer able to bind specifically to the K_Ca_3.1 channel. This experiment indicates that the developed fluorescently labeled ligands bind to the same region of the K_Ca_3.1 channel as senicapoc does. Therefore, specific binding of the new probes to the K_Ca_3.1 channel was confirmed. Thus, the two novel imaging probes **25** and **26** are suitable for fast and simple visualization of the K_Ca_3.1 channels, which appear as bright spots in the fluorescence images.

Since the best results were obtained using the fluorescently labeled ligand **25** (best signal‐to‐noise ratio), this imaging probe was further evaluated. In a control experiment, HEK293 cells not expressing the K_Ca_3.1 channel were incubated with a 10 μm staining solution of **25** following the same protocol as used for the NSCLC cell‐staining experiments. Staining of the HEK293 cells could not be observed (see Figure S2 in the Supporting Information).

Further staining experiments with the fluorescently labeled ligand **25** in living NSCLC cells showed more background signals owing to the incomplete permeation of the cell membrane. However, the punctate staining pattern of the K_Ca_3.1 channel was still visible (see Figure S3 in the Supporting Information).

**Figure 5:**
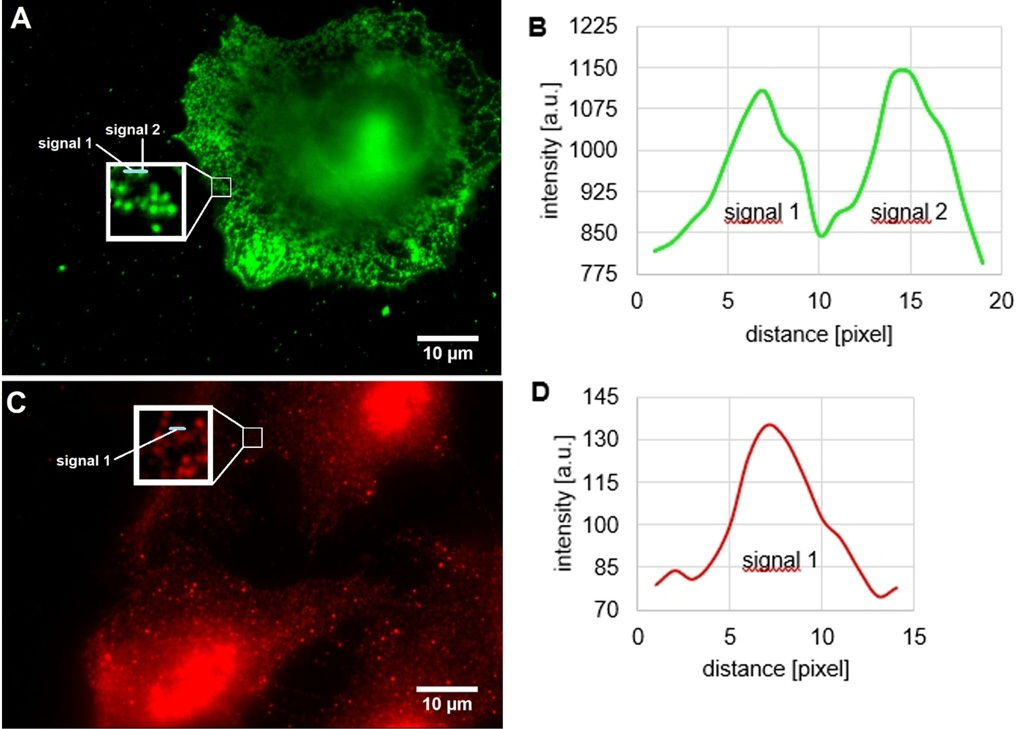
Signal analysis with linescans (blue lines in magnification boxes). 1 pixel≈60 nm. A) NSCLC cells incubated for 10 min with a 10 μm staining solution of imaging probe **25** with 50×50 pixel square, magnification of this square with linescan and 16 signals for K_Ca_3.1 channels. B) Linescan of (A) Intensity profile [arbitrary units] of signal 1 and signal 2. C) NSCLC cells after performing the indirect immunofluorescence assay with 50×50 pixel square, magnification of this square with linescan and 16 signals for K_Ca_3.1 channels. D) Linescan of (C) Intensity profile [arbitrary units] of signal 1.

In order to compare the results obtained by K_Ca_3.1 channel imaging with fluorescently labeled ligand **25** with the results obtained by the indirect immunofluorescence assay, both methods were performed and the K_Ca_3.1 channel density was determined for each experiment. (Figure 5) Therefore, ten squares with a side length of 50 pixels, corresponding to an area of 9 μm^2^, were randomly located in one cell. Signals were analyzed with a linescan (MetaVue software) and were counted as one K_Ca_3.1 channel if the full width at half maximum (FWHM) was ≤5 pixels (ca. 300 nm). In total, 15 cells (150 squares) were analyzed for each staining method.

For both the imaging with fluorescently labeled ligand **25** and the indirect immunofluorescence assay, an average K_Ca_3.1 channel density of 1.79 μm^−2^ was found (compare Figures S4 and S5 in the Supporting Information). This analysis shows, that the same results can be achieved with **25** compared to the indirect immunofluorescence assay but staining with **25** is more than six‐fold faster and more convenient than performing the indirect immunofluorescence assay.

**Figure 6:**
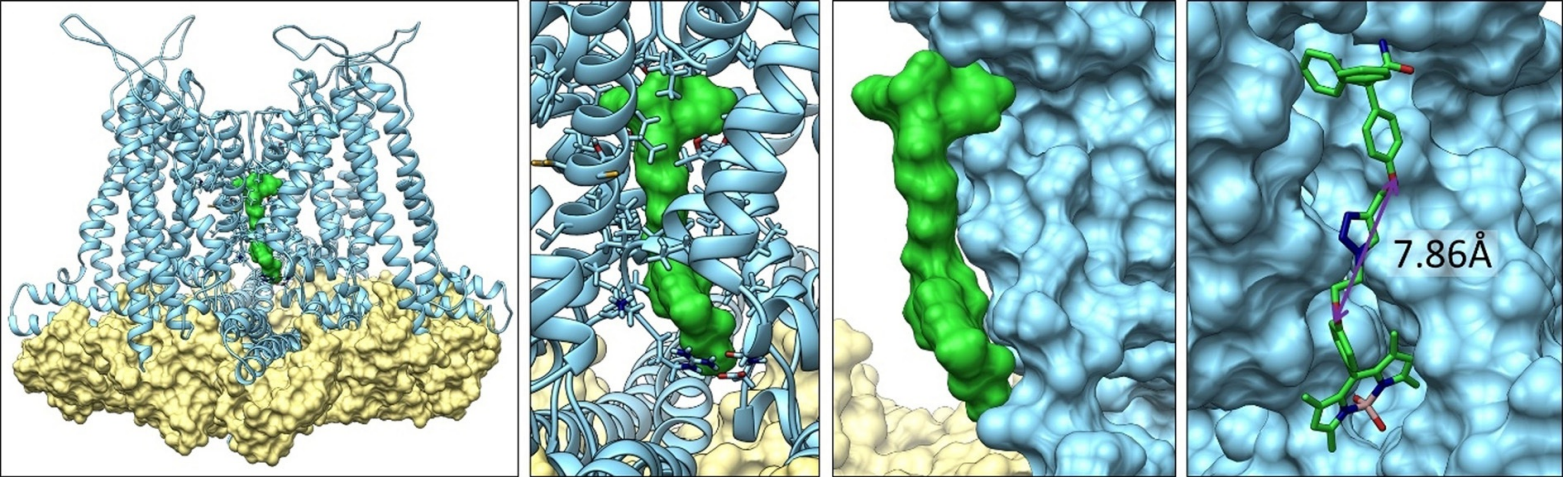
A model of **25** (green) in the K_Ca_3.1 Cryo‐EM structure (pdb 6cn0)^18^ in the bound state II (light blue) with bound calmodulin (khaki). A,B) Overview of **25** binding in the inner pore. C) Surface representation of **25**. D) Molecular representation of **25** and surface representation of ion channel. The given distance of 7.86 Å, indicates the distance between the O‐atoms and thus the length of the linker and the distance between senicapoc moiety and the fluorescent dye.

In order to investigate the binding mode of the fluorescently labeled ligand **25**, molecular modelling of **25** in the available K_Ca_3.1 Cryo‐EM structure (pdb 6cn0)^18^ was performed (Figure 6).

The modelling of **25** indicates a linker length that is nearly perfect to span the distance between the senicapoc moiety and the dye. Figure 6 A,B shows the binding of **25** in the inner pore of the K_Ca_3.1 channel. Figure 6 C,D shows a more detailed view and how **25** fits onto the surface of the K_Ca_3.1 channel. The senicapoc moiety (in the upper part) is binding in a similar region as described for a rosetta homology model of K_Ca_3.1 channel. In this model senicapoc binds also to the upper inner pore of the channel.^19^ The dye is lying outside of the inner pore (lower part) directly on the surface. Therefore, the linker shows a suitable length to connect both moieties through the inner pore. (Figure 6 D) The distance between both O‐atoms is circa 7.86 Å. This analysis confirms a similar binding mode of the fluorescently labeled probe **25** and senicapoc in the inner pore of the K_Ca_3.1 channel.

## Conclusion

A series of fluorescently labeled ligands targeting the K_Ca_3.1 channel was designed, synthesized, and evaluated for their suitability for imaging K_Ca_3.1 channels in vitro in NSCLC cells by fluorescence microscopy. Ligands **25** and **26** labeled with dimethylpyrrole‐based BODIPY dyes and different linkers showed promising results in staining experiments. The typical punctate staining pattern was observed, which was reversed after pre‐incubation with the competitor senicapoc. Imaging of the K_Ca_3.1 channels with the indirect immunofluorescence assay and ligand **25** led to the same density of signals, but imaging with **25** was more than six‐fold faster. Additionally, modelling in the available K_Ca_3.1 Cryo‐EM structure showed similar binding modes for **25** and senicapoc. Compounds **22** and **23** labeled with ethyldimethylpyrrole‐based BODIPY dyes revealed unspecific staining of NSCLC cells without any structure. Whereas ethyl and 3,6,9‐trioxaundecyl moieties are suitable linkers for fluorescently labeled probes, a dodecyl linker (e.g., **24**) led to highly lipophilic compounds resulting in low water solubility, which caused problems in terms of preparing staining solutions. Compound **21** labeled with the BODIPY bearing the thienopyrrole moiety was not suitable as a small‐molecule imaging probe owing to lack of staining the NSCLC cells. The low quantum yields of the connected dye **15,** resulting in low intensity of emitted light is an additional disadvantage of this compound. In summary, dimethylpyrrole‐based BODIPY dyes connected with the targeting senicapoc moiety through an ethyl linker (**25**) represents a promising new small‐molecule probe for selective imaging of K_Ca_3.1 channels of NSCLC cells in vitro and in vivo using a simple, fast, and efficient staining protocol.

## Conflict of interest

The authors declare no conflict of interest.

## Supporting information

As a service to our authors and readers, this journal provides supporting information supplied by the authors. Such materials are peer reviewed and may be re‐organized for online delivery, but are not copy‐edited or typeset. Technical support issues arising from supporting information (other than missing files) should be addressed to the authors.

Supplementary
