# Supplementary material for: Synthesis of Small‐Molecule Fluorescent Probes for the In Vitro Imaging of Calcium‐Activated Potassium Channel KCa3.1
Source: Angew Chem Int Ed Engl. 2020 Mar 17;59(21):8277–84. doi: 10.1002/anie.202001201 (PMC7318252; doi:10.1002/anie.202001201)
Supplement: Supplementary file 1 — Supplementary [file ANIE-59-8277-s001.pdf]

Angewandte Chemie
Eine Zeitschrift der Gesellschaft Deutscher Chemiker

Supporting Information

Synthesis of Small-Molecule Fluorescent Probes for the In Vitro Imaging of Calcium-Activated Potassium Channel $K_{Ca}3.1$

*Kathrin Brömmel, Sarah Maskri, Ivan Maisuls, Christian Paul Konken, Marius Rieke, Zoltan Pethő, Cristian A. Strassert, Oliver Koch, Albrecht Schwab, and Bernhard Wünsch**

anie_202001201_sm_miscellaneous_information.pdf

Content **page**

1. Purity data (HPLC)………………………..…………….………………… S2
2. Additional cell staining experiments………………..…………………… S3
3. Analysis of the $K_{Ca}3.1$ channel density………………………………… S5
4. Molecular Modelling……………………………………………………… S7
5. Experimental part………………………………………………………… S8
   5.1. Chemistry, general………………………………………………………S8
   5.2. Synthetic procedures……………………………………………………S9
   5.3. In vitro studies……………………………………………………………S32
6. ^{1}H and ^{13}C NMR spectra…………………………………………………… S35
7. HPLC traces of key target compounds………………………………… S53
8. Absorption and emission spectra of compounds **15** - **20**…………….. S65
9. Photophysical data of compounds **15** – **20**…………………………….. S71
10. References…………………………………………………………………S79

1. Purity data (HPLC)

Compd.	Purity [%]	Compd.	Purity [%]
**2**	98.1	**18**	94.4
**4**	99.1	**19**	90.4
**5**	91.0	**20**	94.6
**6**	95.1	**21**	95.6
**7**	97.7	**22**	98.7
**8**	99.6	**23**	99.4
**15**	92.1	**24**	98.3
**16**	99.5	**25**	97.7
**17**	94.8	**26**	94.8

2. Additional cell staining experiments

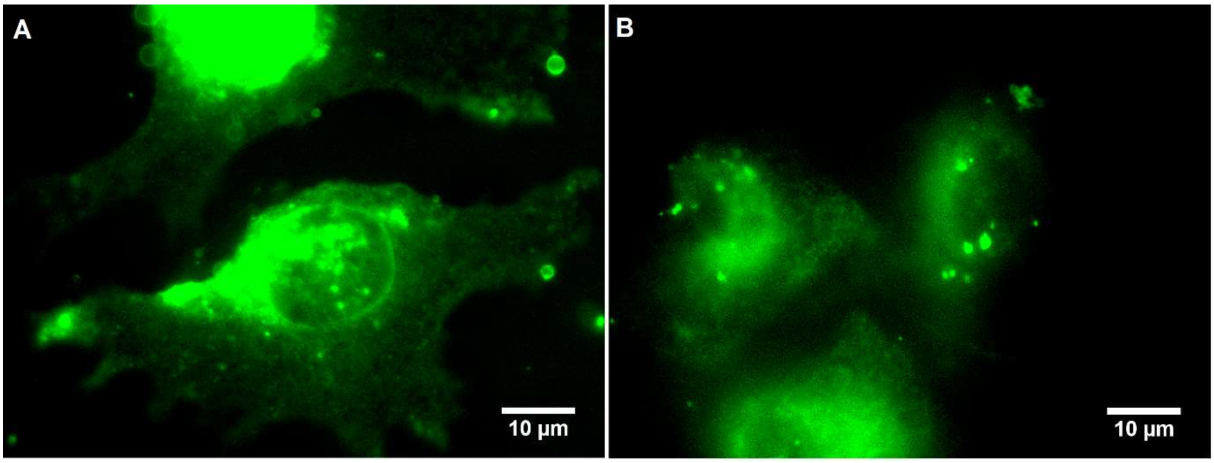

Figure S1: The staining protocol 1 was performed for staining of A549-3R cells as described in the manuscript. Incubation time: 10 min.

A: NSCLC cells incubated with a 10 µM staining solution of imaging probe **22**. **B**: NSCLC cells incubated with a 10 µM staining solution of imaging probe **23**.

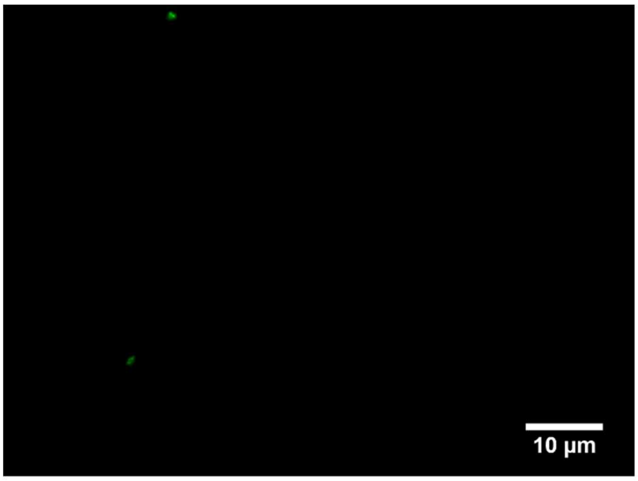

Figure S2: The staining protocol 1 was performed for staining of HEK293 cells (negative control) as described in the manuscript. Incubation time: 10 min

HEK293 cells incubated with a 10 µM staining solution of imaging probe **25**.

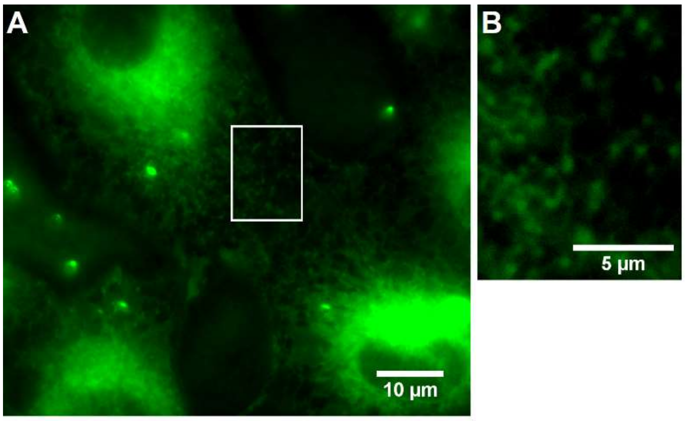

Figure S3: Living A549-3R cells were used. The staining protocol 4 was performed as described in the manuscript. Incubation time: 10 min

A: Living NSCLC cells incubated with a 10 µM staining solution of imaging probe **25**.

B: Magnification of **A** (white box).

3. Analysis of the K_{Ca}3.1 channel density

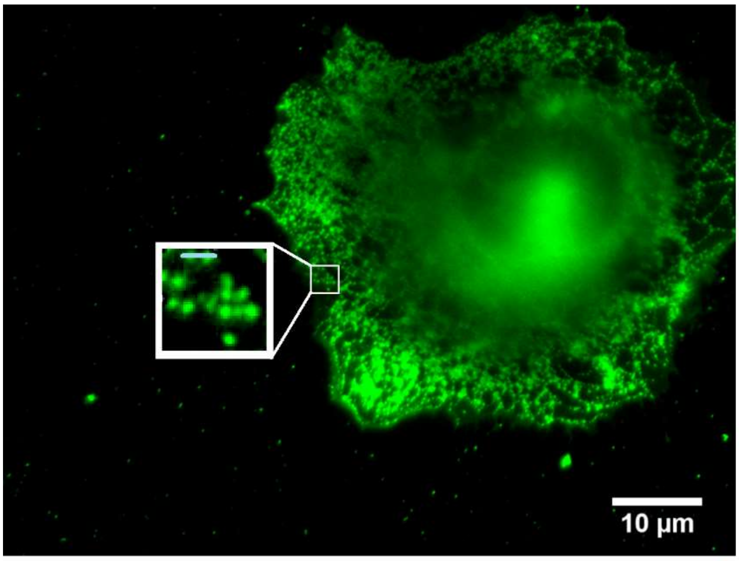

Figure S4: **A**: NSCLC cells incubated with a 10 µM staining solution of imaging probe **25**. **B**: Magnification of **A** (white box) with linescan (blue line).

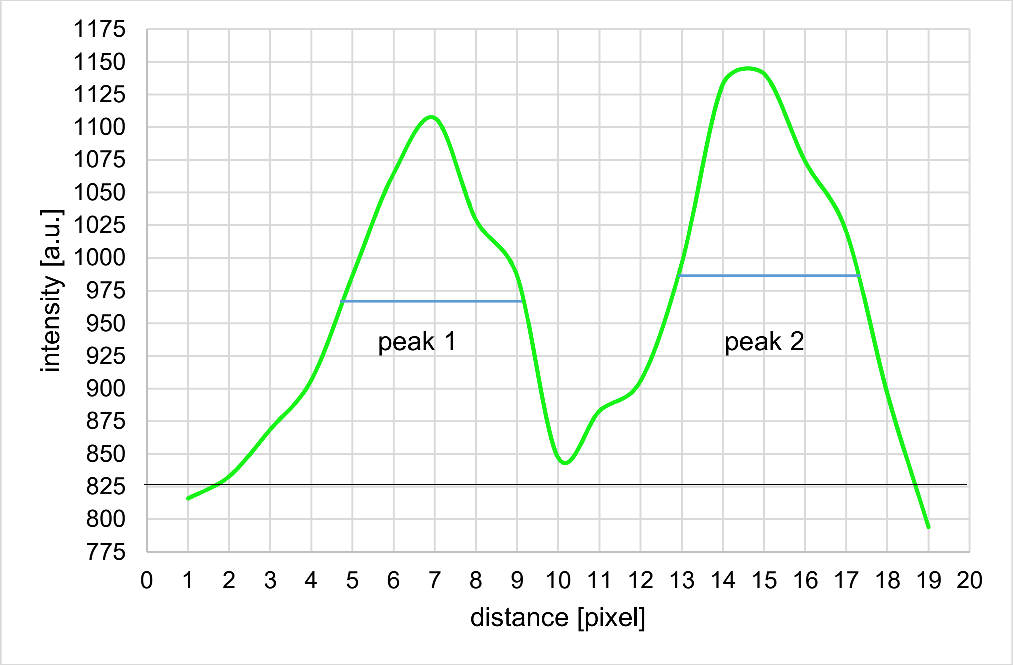

Figure S5: Intensity profile of the linescan in Figure S4. Y axis: intensity in arbitrary units. X-axis: distance in pixel.

At first the zero line is set (black line; (y_{min} = 825)). The *Full Width at Half Maximum* (FWHM, $y_{1/2}$) was calculated for each peak by the following formula:

$$y_{1/2} = [0{,}5(I_{max} + I_{min})]$$

In this example:

Peak 1: I_{min} = 825 a.u. ; I_{max} = 1107 a.u.: $y_{1/2}$ = [0.5 (825 + 1107) = 966 a.u.

Peak 2: I_{min} = 825 a.u. ; I_{max} = 1141 a.u.: $y_{1/2}$ = [0.5 (825 + 1141) = 983 a.u.

Next, the difference of the x-values for $y_{1/2}$ were calculated, which is the FWHM. (Table S1)

Table S1: Calculation of FWHM

Peak	$y_{1/2}$ [a.u.]	x_1 [pixel]	x_2 [pixel]	FWHM [pixel]
peak 1	966	4.7	9.2	4.4
peak 2	983	12.9	17.3	4.4

Signals with FWHM ≤ 5 pixel were counted as $K_{Ca}3.1$ channel.

4. Modelling

The modelling was based on the available Cryo-EM structure (pdb 6cno)[4] and performed with the Molecular Operating Environment program version 2019 (MOE)[5]. At the beginning, a structure preparation was performed including the 3D protonation of the protein (at pH 7.4), deleting distant waters, tethering and fixing atoms, and refining the structure by adjusting and adding missing residues and loop segments. Next, the protein structure was subjected to energy minimization in MOE with a force field AMBER parameters for proteins (ff10)[6], a root-mean-square (RMS) gradient of 0.1 kcal/mol/ Å² was also used. Finally, the cleaned structure was manually inspected. Ligands with senicapoc as a root adding linkers of different length were prepared using MOE and carefully inspected manually. Amber10:EHT parameters and AM1-BCC[7] partial charges for small molecules were used.

The active site was defined based on the core residues of TRAM-34 binding mode from the rosetta-model[8] using a 5 Å sphere to guide the placement. The docking studies were carried using MOE and the triangle matcher placement methodology. The London dG scoring function was taken into account as an initial scoring methodology. The Induced Fit type of post-placement refinement was used. The final energy is evaluated using the Generalized Born solvation model (GB/VI).

Unfortunately, MOE is unable to deduce all the atom type of the dye while docking. These dummy atoms were therefore modelled at the end of the docking and the ligand was minimized afterwards.

5. Experimental part

5.1. Chemistry, general

Unless otherwise noted, moisture sensitive reactions were conducted under dry nitrogen. CH_2Cl_2 was distilled over CaH_2. THF was distilled over sodium/benzophenone. Thin layer chromatography (tlc): Silica gel 60 F254 plates (Merck). Flash chromatography: Silica gel 60, 40–64 µm (Merck); parentheses include: diameter of the column (d), length of the stationary phase (l), fraction size (V), eluent. Automatic flash chromotography: System: Isolera™ One (Biotage®); parentheses include: cartridge-type, eluent, flow rate, fraction volume. Semi-preparative HPLC: Pump: UltiMate 3000, degasser: UltiMate 3000, injection loop (1 ml), UV-detector: UltiMate 3000 variable Wavelength Detector; data acquisition: Chromeleon Client 8.0.0 (Dionex Corpor.), Column: Agilent® Agilent Prep C18 (10µm, 21.1 x 250 mm), PN 410910-102, SN USAZZ01013, Guard column: Agilent® PrepHT Guard Column, PN 82044-901, injection volume: 300 – 800 µL; detection at λ = 210 nm; solvents: A: water; B: acetonitrile; methods: see supporting information. Melting point: Melting point apparatus Mettler Toledo MP50 Melting Point System, uncorrected. MS: microTOF-Q II (Bruker Daltonics); APCI, atmospheric pressure chemical ionization; ESI, electrospray ionization. Nuclear magnetic resonance (NMR) spectra were recorded on Agilent 600-MR (600 MHz for ^{1}H, 151 MHz for ^{13}C) or Agilent 400-MR spectrometer (400 MHz for ^{1}H, 101 MHz for ^{13}C) and on Bruker Avance II 300 MHz or Bruker Avance II 300 MHz spectrometer; δ in ppm related to tetramethylsilane and measured referring to $CHCl_3$ (δ = 7.26 ppm (^{1}H NMR) and δ = 77.2 ppm (^{13}C NMR)) and DMSO-d_6 (δ = 2.54 ppm (^{1}H NMR) and δ = 39.5 ppm (^{13}C NMR)); coupling constants are given with 0.5 Hz resolution; the assignments of ^{13}C and ^{1}H NMR signals were supported by 2-D NMR techniques where necessary. HPLC: Pump: LPG-3400SD, degasser: DG-1210, autosampler: ACC-3000T, UV-detector: VWD-3400RS, interface: DIONEX UltiMate 3000, data acquisition: Chromeleon 7 (Thermo Fisher Scientific); column: LiChrospher® 60 RP-select B (5 µm), LiChroCART® 250-4 mm cartridge; guard column: LiChrospher® 60 RP-select B (5 µm), LiChroCART® 4-4 mm cartridge (No.: 1.50963.0001), manu-CART® NT cartridge holder; flow rate: 1.0 mL/min; injection volume: 5.0 µL; detection at λ = 210 nm; solvents: A: water with 0.05 % (v/v) trifluoroacetic acid; B: acetonitrile with 0.05 % (v/v) trifluoroacetic acid; gradient elution: method 1: (A %): 0-4 min: 90 %, 4-29 min: 90 $\rightarrow$ 0 %, 29-31 min: 0 %, 31-31.5 min: 0 $\rightarrow$ 90 %, 31.5-40 min: 90 %; method 2:

(A %): 0-4 min: 90 %, 4-20 min: 90 → 0 %, 20-31 min: 0 %, 31-31.5 min: 0 → 90 %, 31.5-40 min: 90 %. The purity of all compounds was determined by this method. Unless otherwise mentioned, the purity of all test compounds is higher than 95 %. UV spectroscopy: UV- spectra were measured on an UV-2450 spectrometer from SHIMADZU. Data were processed using the UVProbe Ver.2.21 software from SHIMADZU. Fluorescence spectroscopy: Fluorescence spectra were measured on a Cary Eclipse Fluorescence Spectrophotometer from VARIAN. Data were processed using Cary Eclipse SCAN software; Fluorescence quantum yield: HAMAMATSU PHOTONICS absolute PL quantum yield measurement 54 system (C9920-02), xenon lamp: L9799-01CW xenon lamp, monochromator, detector: C7473 photon55multichannel-detector, Ulbricht-sphere. Analysis and processing of spectra was performed using the U6039-05 56 PLQY software from HAMAMATSU PHOTONICS.

5.2. Synthetic procedures

(4-Bromophenoxy)-(*tert*-butyl)-diphenylsilane (4)[20]

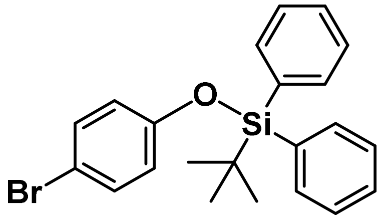

Under N_2 atmosphere, 4-bromophenol (**3**, 10.0 g, 57.8 mmol, 1.0 eq.) and imidazole (7.79 g, 114 mmol, 2.0 eq.) were dissolved in dry DMF (20.0 mL). TBDPS-Cl (18.0 mL, 69.2 mmol, 1.2 eq.) was added and the solution was stirred for 24 h at room temperature. H_2O (20 mL) and CH_2Cl_2 (20 mL) were added. The aqueous layer was separated and extracted with CH_2Cl_2 (3 x 20 mL). The combined organic layers were dried (Na_2SO_4), filtered and concentrated *in vacuo*. The crude product was purified by flash column chromatography (Ø = 8 cm, h = 30 cm, V = 65 mL, cyclohexane: ethyl acetate = 20:1), R_f = 0.64 (cyclohexane: ethyl acetate = 20:1). Colorless solid, mp = 53 °C, yield 22.9 g (96 %), $C_{22}H_{23}BrOSi$ (M_r = 411,4). Purity (HPLC, method 2): 99.1 % (t_R = 22.4 min). HR-MS (APCI): (m/z) = 411.0796 (calcd. 411.0774 for $C_{22}H_{23}BrOSi$ $[M+H]^+$). ^1H NMR (400 MHz, $CDCl_3$): δ (ppm) = 1.09 (s, 9H, C(C*H*$_3$)$_3$), 6.60 – 6.65 (m, 2H, 2-*H*, 6-*H* $_{(OPh)}$), 7.15 – 7.20 (m, 2H, 3-*H*, 5-*H* $_{(OPh)}$)), 7.37 (m, 4H, 3-*H*, 5-*H* $_{(SiPh_2)}$), 7.41 – 7.46 (m, 2H, 4-*H* $_{(SiPh_2)}$), 7.66 – 7.72 (m, 4H, 2-*H*, 6-*H* $_{(SiPh_2)}$). ^{13}C NMR (101 MHz, $CDCl_3$): δ (ppm) = 19.6 (1C, *C*(CH$_3$)$_3$), 26.6 (3C, C(*C*H$_3$)$_3$), 113.5 (1C, C-4 $_{(OPh)}$),

121.6 (2C, C-2, C-6 $_{(OPh)}$), 128.0 (4C, C-3, C-5 $_{(SiPh_2)}$) 130.2 (2C, C-4 $_{(SiPh_2)}$), 132.2 (2C, C-3, C-5 $_{(OPh)}$), 132.6 (2C, C-1 $_{(SiPh_2)}$), 135.6 (4C, C-2, C-6 $_{(SiPh_2)}$), 154.9 (1C, C-1 $_{(OPh)}$).

{4-[(*tert*-Butyldiphenylsilyl)oxy]phenyl}(4-fluorophenyl)(phenyl)methanol (5)

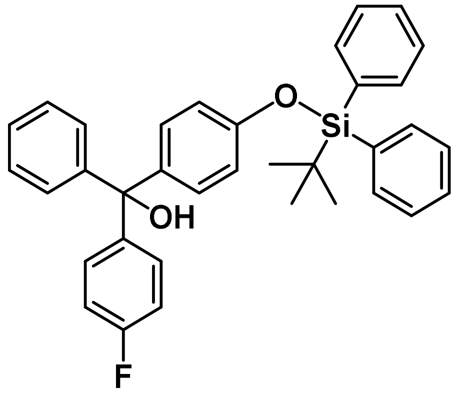

Under N_2 atmosphere, aryl bromide **4** (750 mg, 1.82 mmol, 1.0 eq.) was dissolved in dry THF (8.0 mL). The solution was cooled to -78 °C. *n*-Butyllithium (2.5 M in hexane, 1.0 mL, 2.50 mmol, 1.4 eq.) was added and the mixture was stirred for 1 h at -78 °C. A solution of 4-fluorobenzophenone (400 mg, 2.00 mmol, 1.1 eq.) in THF (6.0 mL) was added. The reaction mixture was allowed to warm up to room temperature. After stirring for 3 h at room temperature, the mixture was poured into ice-cold water (20 mL). The mixture was extracted with CH_2Cl_2 (3 x 20 mL). The combined organic layers were dried (Na_2SO_4), filtered and concentrated *in vacuo*. The residue was purified by flash column chromatography (Ø = 8 cm, h = 30 cm, V = 10 mL, cyclohexane: ethyl acetate = 15:1), R_f = 0.49 (cyclohexane: ethyl acetate = 4:1). Colorless oil, yield 565 mg (58 %), $C_{35}H_{33}FO_2Si$ (M_r = 532.7). Purity (HPLC, method 2): 91.0 % (t_R = 22.1 min). HR-MS (APCI): (m/z) = 515.2310 (calcd. 515.2201 for $C_{35}H_{32}FOSi$ $[M-OH]^+$). ^1H NMR (400 MHz, $CDCl_3$): δ (ppm) = 1.10 (s, 9H, C(C*H*$_3$)$_3$), 2.67 (s, 1H, O*H*), 6.70 (d, *J* = 8.7 Hz, 2H, 3-*H*, 5-*H* $_{(OPh)}$), 6.91 – 6.98 (m, 4H, 2-*H*, 6-*H* $_{(OPh)}$, 3-*H*, 5-*H* $_{(FPh)}$), 7.15 – 7.22 (m, 4H, 2-*H*, 6-*H* $_{(FPh)}$, 2-*H*, 6-*H* $_{(Ph)}$), 7.25 – 7.31 (m, 3H, 3-*H*, 4-*H*, 5-*H* $_{(Ph)}$), 7.33 – 7.39 (m, 4H, 3-*H*, 5-*H* $_{(SiPh_2)}$), 7.40 – 7.45 (m, 2H, 4-*H* $_{(SiPh_2)}$), 7.68 – 7.74 (m, 4H, 2-*H*, 6-*H* $_{(SiPh_2)}$). ^{13}C NMR (151 MHz, $CDCl_3$): δ (ppm) = 19.6 (1C, *C*(CH$_3$)$_3$), 26.7 (3C, C(*C*H$_3$)$_3$), 81.5 (1C, *C*OH), 114.7 (d, *J* = 21.1 Hz, 2C, C-3, C-5 $_{(FPh)}$), 119.3 (2C, C-3, C-5 $_{(OPh)}$), 127.4 (1C, C-4 $_{(Ph)}$), 127.8 (2C, C-2, C-6 $_{(Ph)}$), 127.9 (4C, C-3, C-5 $_{(SiPh_2)}$), 128.0 (2C, C-3, C-5 $_{(Ph)}$), 129.1 (2C, C-2, C-6 $_{(OPh)}$), 129.7 (d, *J* = 8.0 Hz, 2C, C-2, C-6 $_{(FPh)}$), 130.1 (2C, C-4 $_{(SiPh_2)}$), 133.0 (2C, C-1 $_{(SiPh_2)}$), 135.7

(4C, C-2, C-6 (SiPh$_2$)), 139.5 (1C, C-1 (OPh)), 143.0 (d, *J* = 3.1 Hz, 1C, C-1 (FPh)), 147.0 (1C, C-1 (Ph)), 155.1 (1C, C-4 (OPh)), 162.0 (d, *J* = 246.2 Hz, 1C, C-4 (FPh)).

2-{4-[(*tert*-Butyldiphenylsilyl)oxy]phenyl}-2-(4-fluorophenyl)-2-phenylacetonitrile (6)

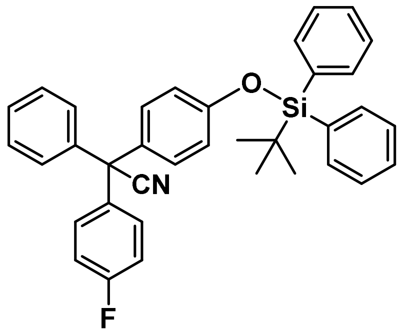

Under N_2 atmosphere, alcohol **5** (213 mg, 0.40 mmol, 1.0 eq.) and $InCl_3$ (17 mg, 0.08 mmol, 0.2 eq.) were dissolved in dry CH_2Cl_2 (4.0 mL). The mixture was stirred at room temperature for 30 min. Trimethylsilyl cyanide (0.1 mL, 0.80 mmol, 2.0 eq.) was added and the mixture was stirred for 1 h at 20 - 40 °C and for 1.5 h at 40 - 50°C. The mixture was poured into an ice-cold solution of KOH (450 mg, 7.98 mmol) and KNa-tartrate (450 mg, 1.60 mmol) in H_2O (5 mL). The mixture was stirred overnight. The layers were separated and the aqueous layer was extracted with CH_2Cl_2 (3 x 5 mL). The combined organic layers were dried (Na_2SO_4), filtered and concentrated *in vacuo*. The residue was purified by flash column chromatography (Ø = 4 cm, h = 20 cm, V = 20 mL, cyclohexane: ethyl acetate = 20:1), R_f = 0.38 (cyclohexane: ethyl acetate = 20:1). Colorless oil, yield 192.3 mg (89 %), $C_{36}H_{32}FNOSi$ (M_r = 541.7). Purity (HPLC, method 2): 95.1 % (t_R = 22.6 min). HR-MS (APCI): (m/z) = 542.2300 (calcd. 542.2310 for $C_{36}H_{33}FNOSi$ $[M+H]^+$). ^{1}H NMR (400 MHz, $CDCl_3$): δ (ppm) = 1.11 (s, 9H, C(C*H*$_3$)$_3$), 6.73 (d, *J* = 8.7 Hz, 2H, 3-*H*, 5-*H* (OPh)), 6.88 (d, *J* = 8.7 Hz, 2H, 2-*H*, 6-*H* (OPh)), 7.00 (t, *J* = 8.6 Hz, 2H, 3-*H*, 5-*H* (FPh)) 7.08 – 7.16 (m, 4H, 2-*H*, 6-*H* (FPh), 2-*H*, 6-*H* (Ph)), 7.29 – 7.34 (m, 3H, 3-*H*, 4-*H*, 5-*H* (Ph)), 7.34 – 7.39 (m, 4H, 3-*H*, 5-*H* (SiPh$_2$)) 7.41 – 7.46 (m, 2H, 4-*H* (SiPh$_2$)), 7.67 – 7.72 (m, 4H, 2-*H*, 6-*H* (SiPh$_2$)). ^{13}C NMR (151 MHz, $CDCl_3$): δ (ppm) = 19.6 (1C, *C*(CH$_3$)$_3$), 26.6 (3C, C(*C*H$_3$)$_3$), 56.27 (1C, *C*CN), 115.6 (d, *J* = 21.3 Hz, 2C, C-3, C-5 (FPh)), 120.1 (2C, C-3, C-5 (OPh)), 123.6 (1C, *C*N), 127.9 (2C, C-3, C-5 (SiPh$_2$)), 128.3 (1C, C-4 (Ph)), 128.7 (2C, C-2, C-6 (Ph)), 128.8 (2C, C-3, C-5 (Ph)), 129.8 (2C, C-2, C-6 (OPh)), 130.2 (2C, C-4, (SiPh$_2$)), 130.7 (d, *J* = 8.9 Hz, 2C, C-2, C-6 (FPh)), 132.5 (1C,C-1 (OPh)), 132.7 (2C, C-1 (SiPh$_2$)) , 135.7 (4C, C-2, C-6 (SiPh$_2$)), 136.5 (d, *J* =

3.4 Hz, 1C, C-1 (FPh)), 140.5 (1C, C-1 (Ph)), 155.8 (1C, C-4 (OPh)), 162.4 (d, *J* = 248.5 Hz, 1C, C-4 (FPh)).

2-(4-Fluorophenyl)-2-(4-hydroxyphenyl)-2-phenylacetonitrile (7)

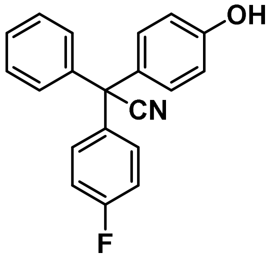

Silyl ether **6** (554 mg, 1.02 mmol, 1.0 eq.) was dissolved in 2-methylbutan-2-ol (5 mL) KOH (630 mg, 11.2 mmol, 11.0 eq.) and KNa-tartrate (620 mg, 2.20 mmol, 2.2 eq.) were added. The mixture was stirred at 100 °C for 5 h. The mixture was cooled down to room temperature. The pH value was adjusted to 6.5 - 7.5 with HCl (1 M) and CH_2Cl_2 (5 mL) was added. The organic layer was washed with water (3 x 5 mL). The organic layer was dried (Na_2SO_4), filtered and concentrated *in vacuo*. The residue was purified by flash column chromatography (Ø = 4 cm, h = 20 cm, V = 20 mL, cyclohexane: ethyl acetate = 4:1), R_f = 0.25 (cyclohexane: ethyl acetate = 4:1).
Colorless solid, mp = 120 °C, yield 272 mg (88 %), $C_{20}H_{14}FNO$ (M_r = 303.3). Purity (HPLC, method 1): 97.7 % (t_R = 22.3 min). HR-MS (APCI): (m/z) = 304.1136 (calcd. 304.1132 for $C_{20}H_{15}FNO$ $[M+H]^+$). ^1H NMR (400 MHz, $CDCl_3$): δ (ppm) = 5.07 (s, 1H, O*H*), 6.81 (d, *J* = 8.7 Hz, 2H, 3-*H*, 5-*H* (OPh)), 7.01 – 7.07 (m, 4H, 2-*H*, 6-*H* (OPh), 3-*H*, 5-*H* (FPh)), 7.16 – 7.22 (m, 4H, 2-*H*, 6-*H* (FPh), 2-*H*, 6-*H* (Ph)), 7.36 (m, 3H, 3-*H*, 4-*H*, 5-*H* (Ph)). ^{13}C NMR (151 MHz, $CDCl_3$): δ (ppm) = 56.3 (1C, *C*CN), 115.7 (2C, 3*C*, 5*C* (OPh)), 115.8 (d, *J* = 21.7 Hz, 2C, C-3, C-5 (FPh)), 123.6 (1C, *C*N), 128.4 (1C, C-4 (Ph)), 128.8 (2C, C-2, C-6 (Ph)), 128.9 (2C, C-3, C-5 (Ph)), 130.3 (2C, C-2, C-6 (OPh)), 130.7 (d, *J* = 7.6 Hz, 2C, C-2, C-6 (FPh)), 132.4 (1C, C-1 (OPh)), 136.5 (d, *J* = 3.5 Hz, 1C, C-1 (FPh)), 140.4 (1C, C-1 (Ph)), 155.6 (1C, C-4 (OPh)), 162.4 (d, *J* = 248.5 Hz, 1C, C-4 (FPh)).

2-(4-Fluorophenyl)-2-(4-hydroxyphenyl)-2-phenylacetamide (2)

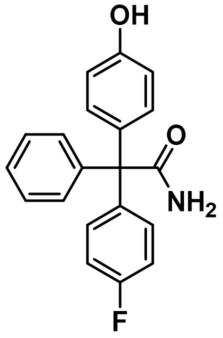

Nitrile **7** (500 mg, 1.65 mmol, 1.0 eq.) was dissolved in ethanol (1,5 mL). A solution of KOH (500 mg, 8.91 mmol, 5.4 eq.) in H_2O (1 mL) was added and the mixture was stirred at 90 °C for 48 h. The pH value was adjusted to 6.5 - 7.5 with HCl (1 M) and CH_2Cl_2 (5 mL) was added. The organic layer was washed with water (3 x 5 mL), dried (Na_2SO_4), filtered and concentrated *in vacuo*. The residue was purified by flash column chromatography (Ø = 4 cm, h = 30 cm, V = 20 mL, CH_2Cl_2: ethyl acetate: CH_3OH = 40:10:1), R_f = 0.36 (CH_2Cl_2: ethyl acetate: CH_3OH = 40:10:1).

Colorless solid, mp = 191 °C, yield 120.7 mg (23 %), $C_{20}H_{16}FNO_2$ (M_r = 321.4). Purity (HPLC, method 1): 98.1 % (t_R = 18.6 min). Exact mass (APCI): (m/z) = 322.1255 (calcd. 322.1238 for $C_{20}H_{17}FNO_2$ $[M+H]^+$). ^{1}H NMR (600 MHz, DMSO-*d*$_6$): δ (ppm) = 6.55 (bs, 1H, N*H*$_2$), 6.69 (d, *J* = 8.7 Hz, 2H, 3-*H*, 5-*H* $_{(OPh)}$), 6.96 (d, *J* = 8.7 Hz, 2H, 2-*H*, 6-*H* $_{(OPh)}$), 7.09 (t, 2H, *J* = 8.9 Hz, 3-*H*, 5-*H* $_{(FPh)}$), 7.15 – 7.24 (m, 5H, 2-*H*, 6-*H* $_{(FPh)}$, 2-*H*, 4-*H,* 6-*H* $_{(Ph)}$), 7.26 – 7.31 (m, 2H, 3-*H*, 5-*H* $_{(Ph)}$), 7.49 (bs, 1H, N*H*$_2$), 9.42 (s, 1H, O*H*). ^{13}C NMR (151 MHz, DMSO-*d*$_6$): δ (ppm) = 65.8 (1C, *C*CONH$_2$), 114.1 (d, *J* = 21.0 Hz, 2C, C-3, C-5 $_{(FPh)}$), 114.5 (2C, C-3, C-5 $_{(OPh)}$), 126.4 (1C, C-4 $_{(Ph)}$), 127.6 (2C, C-3, C-5 $_{(Ph)}$), 129.9 (2C, C-2, C-6 $_{(Ph)}$), 131.1 (2C, C-2, C-6 $_{(OPh)}$), 132.1 (d, *J* = 8.0 Hz, 2C, C-2, C-6 $_{(FPh)}$), 133.8 (1C, C-1 $_{(OPh)}$), 140.6 (d, *J* = 3.4 Hz, 1C, C-1 $_{(FPh)}$), 144.2 (1C, C-1 $_{(Ph)}$), 155.8 (1C, C-4 $_{(OPh)}$), 160.5 (d, *J* = 243.7 Hz, 1C, C-4 $_{(FPh)}$), 174.4 (1C, *C*ONH$_2$).

2-(4-Fluorophenyl)-2-phenyl-2-[4-(prop-2-yn-1-yloxy)phenyl]acetamide (8)

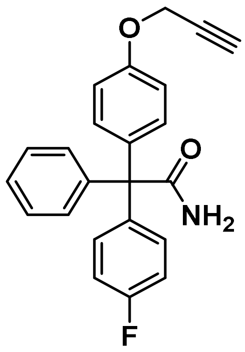

Under N_2 atmosphere, amide **2** (226 mg, 0.70 mmol, 1.0 eq.) and Cs_2CO_3 (343 mg, 1.05 mmol, 1.5 eq.) were dissolved in dry DMF (2 mL). The mixture was stirred at room temperature for 10 min. Propargyl bromide (80 % wt solution in toluene, 0.12 mL, 1.11 mmol, 1.6 eq.) was added dropwise. The mixture was stirred for 30 min. LiCl solution (5 % wt in H_2O, 5 mL) was added and the mixture was stirred for 15 min. The organic layer was separated and washed with LiCl solution (5 % wt in H_2O, 3 x 5 mL), dried (Na_2SO_4), filtered and concentrated *in vacuo*. The crude product was purified by flash column chromatography (Ø = 3 cm, h = 30 cm, V = 20 mL, CH_2Cl_2: CH_3OH = 20:1), R_f = 0.53 (CH_2Cl_2: CH_3OH = 20:1). Colorless solid, mp = 134 °C, yield 226 mg (90 %), $C_{23}H_{18}FNO_2$ (M_r = 359.4). Purity (HLPC, method 1): 99.6 % (t_R = 21.5 min). HR-MS: (APCI): (m/z) = 360.1487 (calcd. 360.1394 for $C_{23}H_{19}FNO_2$ $[M+H]^+$). ^1H NMR (400 MHz, DMSO-d_6): δ (ppm) = 3.56 (t, *J* = 2.3 Hz, 1H, $CH_2C{\equiv}CH$), 4.77 (d, *J* = 2.4 Hz, 2H, $CH_2C{\equiv}CH$), 6.64 (bs, 1H, N*H*2), 6.92 (d, *J* = 8.7 Hz, 2H, 3-*H*, 5-*H* (OPh)), 7.07 – 7.15 (m, 4H, 2-*H*, 6-*H* (OPh), 3-*H*, 5-*H* (FPh)), 7.16 – 7.27 (m, 5H, 2-*H*, 6-*H* (FPh), 2-*H*, 4-*H,* 6-*H* (Ph)), 7.27 – 7.34 (m, 2H, 3-*H*, 5-*H* (Ph)), 7.51 (bs, 1H, N*H*2). ^{13}C NMR (101 MHz, DMSO-d_6): δ (ppm) = 55.8 (1C, *C*H2), 66.4 (1C, *C*CONH2), 78.7 (1C, C≡*C*H), 79.7 (1C, *C*≡CH), 114.3 (2C, C-3, C-5 (OPh)), 114.7 (d, *J* = 21.1 Hz, 2C, C-3, C-5 (FPh)), 126.9 (1C, C-4 (Ph)), 128.2 (2C, C-3, C-5 (Ph)), 130.4 (2C, C-2, C-6 (Ph)), 131.6 (2C, C-2, C-6 (OPh)), 132.5 (d, *J* = 8.0 Hz, 2C, C-2, C-6 (FPh)), 136.8 (1C, C-1 (OPh)), 140.8 (d, *J* = 3.2 Hz, 1C, C-1 (FPh)), 144.4 (1C, C-1 (Ph)), 156.1 (1C, C-4 (OPh)), 161.0 (d, *J* = 243.4 Hz, 1C, C-4 (FPh)), 174.6 (*C*ONH2).

Synthesis of BODIPY dyes 15-20

General procedure

All BODIPY dyes were synthesized under oxygen, moisture and light free conditions. The respective pyrrole derivative **12-14** and the corresponding benzaldehyde **9-11** were dissolved in dry CH_2Cl_2 (150 mL/mmol) containing molecular sieves 3Å (0.5 g/mmol). Trifluoroacetic acid (2 drops) was added and the mixture was stirred at room temperature for 24 h. A solution of 2,3,5,6-tetrachloro-1,4-benzoquinone in CH_2Cl_2 (80 mL/mmol) was added and the mixture was stirred at room temperature for 30 min. Et_3N was added and the mixture was stirred at room temperature for 15 min. $BF_3 \cdot OEt_2$ was added dropwise and the mixture was stirred at room temperature for 12 h. The mixture was filtered, H_2O (100 mL/mmol) was added and the mixture was stirred at room temperature for 2 h. The organic layer was separated, washed with H_2O (3 x 100 mL/mmol), dried (Na_2SO_4), filtered and concentrated *in vacuo*. The crude product was purified as mentioned.

10,10-Difluoro-4,6-dimethyldithieno[2,3-b:3',2'-g]-4-bora-3a,4a-diaza-s-indacene (15)

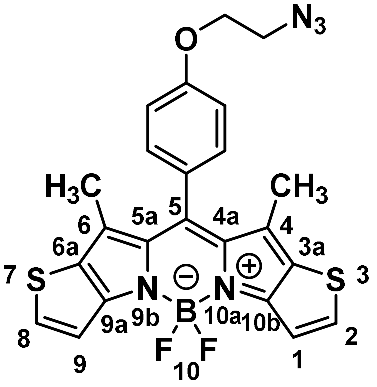

According to General Procedure A, 6-methylthieno[3,2-b]pyrrole (**12** 759 mg, 5.53 mmol, 2.0 eq.), 4-(2-azidoethoxy)benzaldehyde (**9**, 490 mg, 2.77 mmol, 1.0 eq.), 2,3,5,6-tetrachloro-1,4-benzoquinone (680 mg, 2.77 mmol, 1.0 eq.), Et_3N (2.20 mL, 15.8 mmol, 5.7 eq.) and $BF_3 \cdot OEt_2$ (2.10 mL, 16.6 mmol, 6 eq.) were reacted stepwise in CH_2Cl_2. The crude product was purified by flash column chromatography; column 1: (Ø 8 cm, h = 25 cm, V = 40 mL, CH_2Cl_2: CH_3OH = 99:1), column 2: (Ø 6 cm, h = 30 cm, V = 10 mL, cyclohexane: ethyl acetate = 4:1 → 2:1), R_f = 0.33 (cyclohexane: ethyl acetate = 4:1). Purple solid, mp = 85 °C, yield 94 mg (7 %), $C_{23}H_{18}BF_2N_5OS_2$ (M_r = 493.4). Purity (HLPC, method 2): 92.1 % (t_R = 19.7 min). HR-MS (APCI): (m/z) = 474.1061 (calcd. 474.1029 for $C_{23}H_{18}BFN_5OS_2$ $[M-F]^+$). ^1H NMR (600 MHz, $CDCl_3$): δ

(ppm) = 1.64 (s, 6H, 4-CH_3, 6-CH_3 (indacene)), 3.69 (t, J = 4.9 Hz, 2H, $OCH_2CH_2N_3$), 4.24 (t, J = 4.9 Hz, 2H, $OCH_2CH_2N_3$), 7.10 (d, J = 8.7 Hz, 2H, 3-H, 5-H (Ph)), 7.15 (d, J = 5.4 Hz, 2H, 2-H, 8-H (thieno)), 7.28 (d, J = 8.7 Hz, 2H, 2-H, 6-H (Ph)), 7.61 (d, J = 5.4 Hz, 2H, 1-H, 9-H (thieno)).^{13}C NMR (151 MHz, $CDCl_3$): δ (ppm) = 14.4 (2C, 4-CH_3, 6-CH_3 (indacene)), 50.4 (1C, $OCH_2CH_2N_3$), 67.2 (1C, $OCH_2CH_2N_3$), 114.3 (2C, 2-C, 8-C (thieno)), 115.5 (2C, C-3, C 5 (Ph)), 126.9 (1C, C-5 (indacene)), 129.8 (2C, C-2, C-6 (Ph)), 133.8 (2C, C-1, C-9 (thieno)), 134.1 (2C, C-4a, C-5a (indacene)), 138.0 (2C, C-4, C-6 (indacene)), 140.4 (1C, C-4 (Ph)), 145.7 (2C, C-3a, C-6a (indacene)), 157.1 (2C, C-9a, C-10b (indacene)), 159.4 (1C, C-1 (Ph)). ^{11}B NMR (128 MHz, $CDCl_3$): δ (ppm) = 0.75 (t, *J* = 32.7 Hz). ^{19}F NMR (376 MHz, $CDCl_3$): δ (ppm) = -146.28 (q, *J* = 32.3 Hz). UV absorption: (CH_3CN): λ_{max} = 553 nm, (CH_3Cl/CH_3OH): λ_{max} = 559 nm, Fluorescence emission: (CH_3CN): λ_{max} = 571 nm, (CH_3Cl/CH_3OH): λ_{max} = 574 nm, Quantum yield (CH_3Cl/CH_3OH): rt: 5 ± 2 %, 77K: 12 ± 2 %.

8-[4-(2-Azidoethoxy)phenyl]-2,6-diethyl-4,4-difluoro-1,3,5,7-tetramethyl-4-bora-3a,4a-diaza-s-indacene (16)

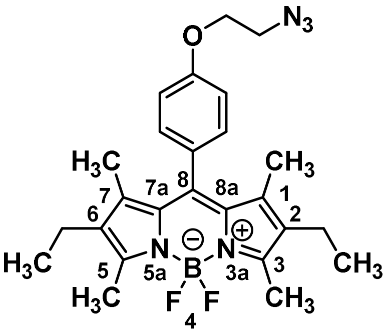

According to General Procedure A, 3-ethyl-2,4-dimethylpyrrole (**13**, 0.86 mL, 6.38 mmol, 2.0 eq.), 4-(2-azidoethoxy)benzaldehyde (**9**, 565 mg, 3.19 mmol, 1.0 eq.), 2,3,5,6-tetrachloro-1,4-benzoquinone (813 mg, 3.19 mmol, 1.0 eq.), Et_3N (2.43 mL, 17.5 mmol, 5.5 eq.) and $BF_3{\cdot}OEt_2$ (2.43 mL, 19.2 mmol, 6 eq.) were reacted stepwise in CH_2Cl_2. The crude product was purified by flash column chromatography (Ø 6 cm, h = 30 cm, V = 20 mL, CH_2Cl_2: cyclohexane = 5:1) and semi-preparative HPLC (method A), R_f = 0.27 (CH_2Cl_2: cyclohexane = 1:1). Red solid, mp = 157 °C, yield 213 mg (14 %), $C_{25}H_{30}BF_2N_5O$ (M_r = 465.4). Purity (HLPC, method 1): 99.5 % (t_R = 27.2 min). HR-MS (APCI): (m/z) = 466.2621 (calcd. 466.2589 for $C_{25}H_{31}BF_2N_5O$ $[M+H]^+$). ^{1}H NMR (600 MHz, $CDCl_3$): δ (ppm) = 0.98 (t, *J* = 7.6 Hz, 6H, 2-CH_2CH_3, 6-CH_2CH_3), 1.33 (s, 6H, 1-CH_3, 7-CH_3), 2.30 (q, *J* = 7.6 Hz, 4H, 2-CH_2CH_3, 6-CH_2CH_3), 2.53 (s, 6H, 3-CH_3, 5-CH_3), 3.67 (t, *J* = 5.0 Hz, 2H, $OCH_2CH_2N_3$), 4.22 (t, *J* = 5.0 Hz,

2H, OC*H*2CH2N3), 7.03 (d, *J* = 8.7 Hz, 2H, 3-*H*, 5-*H* (Ph)), 7.19 (d, *J* = 8.6 Hz, 2H, 2-*H*, 6-*H* (Ph)). ^{13}C NMR (151 MHz, $CDCl_3$): δ (ppm) = 12.0 (2C, 1-CH_3, 7-CH_3), 12.6 (2C, 3-CH_3, 5-CH_3), 14.8 (2C, 2-CH_2*C*H_3, 6-CH_2*C*H_3), 17.2 (2C, 2-*C*H_2CH_3, 6-*C*H_2CH_3), 50.4 (1C, O*C*$H_2CH_2N_3$), 67.1 (1C, OCH_2*C*H_2N_3), 115.4 (2C, C-3 (Ph), C-5 (Ph)), 128.8 (1C, C-8), 129.8 (2C, C-2, C-6 (Ph)), 131.3 (2C, C-7a, C-8a), 132.9 (2C, C-2, C-6), 138.5 (2C, C-1, C-7), 140.1 (1C, C-4 (Ph)), 153.8 (2C, C-3, C-5), 158.8 (1C, C-1 (Ph)). ^{11}B NMR (96 MHz, $CDCl_3$): δ (ppm) = 0.82 (t, *J* = 33.5 Hz). ^{19}F NMR (282 MHz, $CDCl_3$): δ (ppm) = -146.29 (q, *J* = 33.1 Hz). UV absorption: (CH_3CN): λ_{max} = 521 nm, (CH_3Cl/CH_3OH): λ_{max} = 524 nm, Fluorescence emission: (CH_3CN): λ_{max} = 533 nm, (CH_3Cl/CH_3OH): λ_{max} = 537 nm, Quantum yield (CH_3Cl/CH_3OH): rt: 73 ± 2 %, 77K: 100 ± 2 %.

8-[4-(12-Azido-1,4,7,10-tetraoxadodecyl)phenyl]-2,6-diethyl-4,4-difluoro-1,3,5,7-tetramethyl-4-bora-3a,4a-diaza-s-indacene (17)[13]

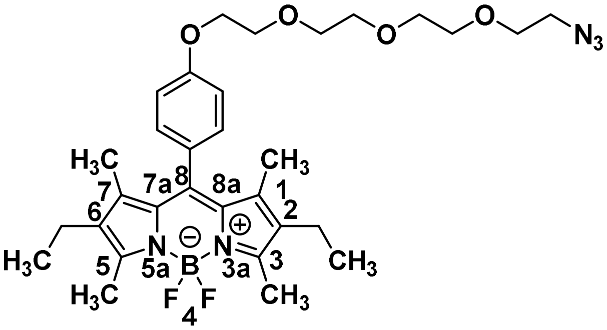

According to General Procedure A, 3-ethyl-2,4-dimethylpyrrole (**13**, 0.43 mL, 3.19 mmol, 2.0 eq.), 4-(12-azido-1,4,7,10-tetraoxadodecyl)benzaldehyde (**10**, 510 mg, 1.58 mmol, 1.0 eq.), 2,3,5,6-tetrachloro-1,4-benzoquinone (389 mg, 1.58 mmol, 1.0 eq.), Et_3N (1.20 mL, 8.66 mmol, 5.5 eq.) and $BF_3{\cdot}OEt_2$ (1.20 mL, 9.47 mmol, 6 eq.) were reacted stepwise in CH_2Cl_2. The crude product was purified by flash column chromatography; column 1: (Ø 8 cm, h = 35 cm, V = 60 mL, CH_2Cl_2: CH_3OH = 99:1); product containing fractions were combined, concentrated *in vacuo* and purified by column 2: (Ø 8 cm, h = 25 cm, V = 20 mL, cyclohexane: ethyl acetate = 3:2), R_f = 0.35 (cyclohexane: ethyl acetate = 3:2). Red solid, mp = 73 °C, yield 286 mg (30 %), $C_{31}H_{42}BF_2N_5O_4$ (M_r = 597.5). Purity (HLPC, method 1): 94.8 % (t_R = 26.7 min). HR-MS (ESI): (m/z) = 598.3399 (calcd. 598.3376 for $C_{31}H_{43}BF_2N_5O_4$ $[M+H]^+$). ^{1}H NMR (600 MHz, $CDCl_3$): δ (ppm) = 0.98 (t, *J* = 7.6 Hz, 6H, 2-CH_2C*H*3, 6-CH_2C*H*3 (indacene)), 1.32 (s, 6H, 1-C*H*3, 7-C*H*3 (indacene)), 2.30 (q, *J* = 7.6 Hz, 4H, 2-C*H*2CH3, 6-C*H*2CH3 (indacene)), 2.52 (s, 6H, 3-C*H*3, 5-C*H*3 (indacene)), 3.39 (t, *J* = 5.3 Hz, 2H, 12-C*H*2 (dodecyl)), 3.66 – 3.71

(m, 6H, 11-CH_2, 9-CH_2, 8-CH_2 (dodecyl)), 3.71 – 3.75 (m, 2H, 6-CH_2 (dodecyl)), 3.75 – 3.79 (m, 2H, 5-CH_2 (dodecyl)), 3.91 (t, J = 4.6 Hz, 2H, 3-CH_2 (dodecyl)), 4.19 (t, J = 4.6 Hz, 2H, 2-CH_2 (dodecyl)), 7.01 (d, J = 8.6 Hz, 2H), 7.15 (d, J = 8.6 Hz, 2H). ^{13}C NMR (151 MHz, $CDCl_3$): δ (ppm) = 12.0 (2C, 1-CH_3, 7-CH_3 (indacene)), 12.6 (2C, 3-CH_3, 5-CH_3 (indacene)), 14.8 (2C, 2-CH_2CH_3, 6-CH_2CH_3 (indacene)), 17.2 (2C, 2-CH_2CH_3, 6-CH_2CH_3 (indacene)), 50.9 (1C, C-12 (dodecyl)), 67.6 (1C, C-2 (dodecyl)), 69.9 (1C, C-3 (dodecyl)), 70.2, 70.8, 70.9, 71.1 (5C, C-5, C-6, C-8, C-9, C-11 (dodecyl)), 115.2 (2C, C-3, C-5 (Ph)), 128.2 (1C, C-8 (indacene)), 129.6 (2C, C-2, C-6 (Ph)), 131.3 (2C, C-7a, C-8a (indacene)), 132.8 (2C, C-2, C-6 (indacene)), 138.6 (2C, C-1, C-7 (indacene)), 140.4 (1C, C-4 (Ph)), 153.6 (2C, C-3, C-5 (indacene)), 159.3 (1C, C-1 (Ph)). ^{11}B NMR (96 MHz, $CDCl_3$): δ (ppm) = 0.82 (t, J = 33.7 Hz). ^{19}F NMR (282 MHz, $CDCl_3$): δ (ppm) = -146.32 (q, J = 32.9 Hz). UV absorption: (CH_3CN): λ_{max} = 521 nm, (CH_3Cl/CH_3OH): λ_{max} = 524 nm, Fluorescence emission: (CH_3CN): λ_{max} = 533 nm, (CH_3Cl/CH_3OH): λ_{max} = 537 nm, Quantum yield (CH_3Cl/CH_3OH): rt: 63 ± 2 %, 77K: 100 ± 2 %.

8-[4-(12-Azidododecyloxy)phenyl]-2,6-diethyl-4,4-difluoro-1,3,5,7-tetramethyl-4-bora-3a,4a-diaza-s-indacene (18)[13]

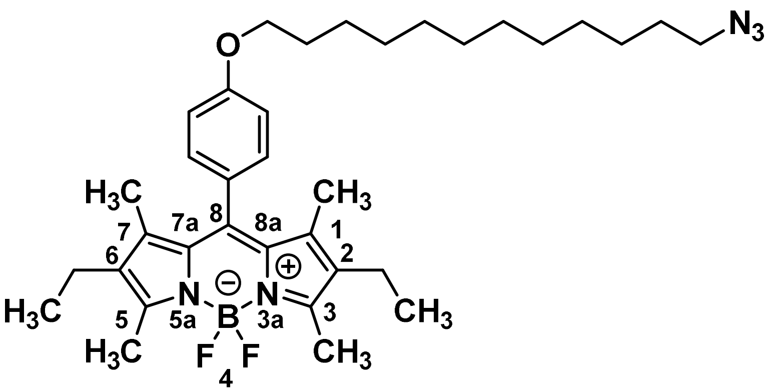

According to General Procedure A, 3-ethyl-2,4-dimethylpyrrole (**13**, 0.62 mL, 4.59 mmol, 2.0 eq.), 4-[(12-azidododecyl)oxy]benzaldehyde (**11**, 760 mg, 2.29 mmol, 1.0 eq.), 2,3,5,6-tetrachloro-1,4-benzoquinone (563 mg, 2.29 mmol, 1.0 eq.), triethylamine (1.75 mL, 12.6 mmol, 5.5 eq.) and $BF_3 \cdot OEt_2$ (1.75 mL, 13.8 mmol, 6 eq.) were reacted in CH_2Cl_2. The crude product was purified by flash column chromatography; column 1: (Ø 8 cm, h = 35 cm, V = 60 mL, cyclohexane: ethyl acetate = 4:1 column 2: (Ø 6 cm, h = 30 cm, V = 20 mL, cyclohexane: ethyl acetate = 4:1); column 3: MP Alumina N, Act.I (MP Biomedicals) was used as stationary phase (Ø 3 cm, h = 25 cm, V = 10 mL, cyclohexane: ethyl acetate = 20:1), column 4: MP Alumina

N, Act.I (MP Biomedicals) was used as stationary phase (Ø 4 cm, h = 15 cm, V = 10 mL, cyclohexane: ethyl acetate = 20:1); automatic flash column chromatography 1: (cartridge: SNAP C18, 30g (Biotage®), 80% → 100% CH_3CN in water, 25 mL/min); automatic flash column chromatography2 : (cartridge: SNAP C18, 30g (Biotage®), 80% → 100% CH_3CN in water, 25 mL/min), R_f = 0.48 (cyclohexane: ethyl acetate = 6:1). Red solid, mp = 76 °C, yield 304 mg (22 %), $C_{35}H_{50}BF_2N_5O$ (M_r = 605.6). Purity (HLPC, method 2): 97.3 % (t_R = 34.1 min). HR-MS (ESI): (m/z) = 606.4196 (calcd. 606.4155 for $C_{35}H_{51}BF_2N_5O$ $[M+H]^+$). ^{1}H NMR (600 MHz, $CDCl_3$): δ (ppm) = 0.98 (t, *J* = 7.5 Hz, 6H, 2-CH_2CH_3, 6-CH_2CH_3 (indacene)), 1.28 – 1.32 (m, 8H, 4-C*H*2, 5-C*H*2, 6-C*H*2, 7-C*H*2 (dodecyl)), 1.34 (s, 6H, 1-C*H*3, 7-C*H*3 (indacene)), 1.35 – 1.41 (m, 4H, 8-C*H*2, 9-C*H*2 (dodecyl)), 1.46 – 1.52 (quint, *J* = 7.7 Hz, 2H, 3-C*H*2 (dodecyl)), 1.58 – 1.63 (m, 4H, 10-C*H*2, 11-C*H*2 (dodecyl)), 1.79 – 1.86 (quint, *J* = 7.7 Hz, 2H, 2-C*H*2 (dodecyl)), 2.30 (quint, *J* = 7.7 Hz, 4H, 2-C*H*2CH3, 6-C*H*2CH3 (indacene)), 2.52 (s, 6H, 3-C*H*3, 5-C*H*3 (indacene)), 3.26 (t, *J* = 7.0 Hz, 2H, 12-C*H*2 (dodecyl)), 4.01 (t, *J* = 6.6 Hz, 2H, 1-C*H*2 (dodecyl)), 6.99 (d, *J* = 8.5 Hz, 2H, 3-*H*, 5-*H* (Ph)), 7.14 (d, *J* = 8.5 Hz, 2H, 2-*H*, 6-*H* (Ph)). ^{13}C NMR (151 MHz, $CDCl_3$): δ (ppm) = 12.0 (2C, 1-CH_3, 7-CH_3 (indacene)), 12.6 (2C, 3-CH_3, 5-CH_3 (indacene)), 14.8 (2C, 2-CH2*C*H3, 6-CH2*C*H3 (indacene)), 17.2 (2C, 2-*C*H2CH3, 6-*C*H2CH3 (indacene)), 26.2, 26.9, 29.0, 29.3, 29.4, 29.6, 29.6, 29.7, 29.7, 29.9 (10C, C-2, C-3, C-4, C-5, C-6, C-7, C-8, C-9, C-10, C-11 (dodecyl)) , 51.6 (1C, C-12 (dodecyl)), 68.3 (1C, C-2 (dodecyl)), 115.1 (2C, C-3, C-5 (Ph)), 127.8 (1C, C-8 (indacene)), 129.6 (2C, C-2, C-6 (Ph)), 131.3 (2C, C-1a, C-7a (indacene)), 132.7 (2C, C-2, C-6 (indacene)), 138.6 (2C, C-1, C-7 (indacene)), 140.5 (1C, C-4 (Ph)), 153.6, (2C, C-3, C-5 (indacene)), 159.7 (1C, C-1 (Ph)). ^{11}B NMR (96 MHz, $CDCl_3$): δ (ppm) = 0.83 (t, *J* = 33.7 Hz). ^{19}F NMR (282 MHz, $CDCl_3$): δ (ppm) = -146.32 (q, *J* = 33.0 Hz). UV absorption: (CH_3CN): λ_{max} = 521 nm, (CH_3Cl/CH_3OH): λ_{max} = 524 nm, Fluorescence emission: (CH_3CN): λ_{max} = 532 nm, (CH_3Cl/CH_3OH): λ_{max} = 535 nm, Quantum yield (CH_3Cl/CH_3OH): rt: 64 ± 2 %, 77K: 100 ± 2 %.

8-[4-(2-Azidoethoxy)phenyl]-4,4-difluoro-1,3,5,7-tetramethyl-4-bora-3a,4a-diaza-s-indacene (19)[14]

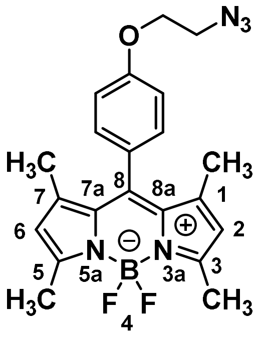

According to General Procedure A, 2,4-dimethylpyrrole (**14**, 0.75 mL, 7.28 mmol, 2.0 eq.), 4- (2-azidoethoxy)benzaldehyde (**9**, 645 mg, 3.64 mmol, 1.0 eq.), 2,3,5,6-tetrachloro-1,4-benzoquinone (895 mg, 3.64 mmol, 1.0 eq.), Et_3N (2.78 mL, 20.0 mmol, 5.5 eq.) and BF_3·OEt_2 (2.77 mL, 21.86 mmol, 6 eq.) were reacted stepwise in CH_2Cl_2. The crude product was purified by flash column chromatography; column 1: (Ø = 8 cm, h = 20 cm, V = 20 mL, CH_2Cl_2), column 2: (Ø = 6 cm, h = 10 cm, V = 10 mL, CH_2Cl_2), R_f = 0.45 (CH_2Cl_2). Orange solid, mp = 140 °C, yield 239 mg (16 %), $C_{21}H_{22}BF_2N_5O$ (M_r = 409.3). Purity (HLPC, method 1): 90.4 % (t_R = 24.3 min). HR-MS (ESI): (m/z) = 410.1947 (calcd. 410.1962 for $C_{21}H_{23}BF_2N_5O$ $[M+H]^+$). ^1H NMR (400 MHz, DMSO-d_6): δ (ppm) = 1.40 (s, 6H, 1-CH_3, 7-CH_3), 2.44 (s, 6H, 3-CH_3, 5-CH_3), 3.70 (t, *J* = 4.9 Hz, 2H, $OCH_2CH_2N_3$), 4.25 (t, *J* = 4.6 Hz, 2H, $OCH_2CH_2N_3$), 6.17 (s, 2H, 2-C*H*, 6-C*H*), 7.13 (d, *J* = 8.7 Hz, 2H, 3-*H*, 5-*H* (Ph)), 7.28 (d, *J* = 8.7 Hz, 2H, 2-*H*, 6-*H* (Ph)). ^{13}C NMR (101 MHz, DMSO-d_6): δ (ppm) = 14.2 (4C, 1-CH_3, 3-CH_3, 5-CH_3, 7-CH_3), 49.6 (1C, $OCH_2CH_2N_3$), 66.8 (1C, $OCH_2CH_2N_3$), 115.2 (2C, C-3, C-5 (Ph)), 121.3 (2C, C-2, C-6), 126.4 (1C, C-8), 129.2 (2C, C-2, C-6 (Ph)), 131.1 (2C, C-7a, C-8a), 142.0 (1C, C-1 (Ph)), 142.7 (2C, C-1, C-7), 154.7 (2C, C-3, C-5), 158.6 (1C, C-4 (Ph)). ^{11}B NMR (128 MHz, $CDCl_3$): δ (ppm) = 0.78 (t, *J* = 33.3 Hz). ^{19}F NMR (376 MHz, $CDCl_3$): δ (ppm) = -146.22 (q, *J* = 32.6 Hz). UV absorption: (CH_3CN): λ_{max} = 497 nm, (CH_3Cl/CH_3OH): λ_{max} = 500 nm, Fluorescence emission: (CH_3CN): λ_{max} = 507 nm, (CH_3Cl/CH_3OH): λ_{max} = 510 nm, Quantum yield (CH_3Cl/CH_3OH): rt: 41 ± 2 %, 77K: 100 ± 2 %.

8-[4-(12-Azido-1,4,7,10-tetraoxadodecyl)phenyl]-4,4-difluoro-1,3,5,7-tetramethyl-4-bora-3a,4a-diaza-s-indacene (20)

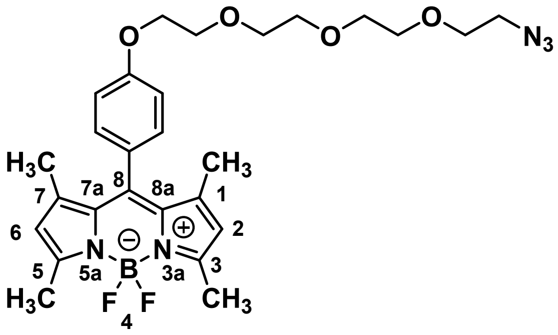

According to General Procedure A, 2,4-dimethylpyrrole (**14**, 0.34 mL, 3.30 mmol, 2.0 eq.), 4-(12-Azido-1,4,7,10-tetraoxadodecyl)benzaldehyde (**10**, 529 mg, 1.63 mmol, 1.0 eq.), 2,3,5,6-tetrachloro-1,4-benzoquinone (402 mg, 1.63 mmol, 1.0 eq.), Et_3N (1.26 mL, 9.03 mmol, 5.5 eq.) and $BF_3 \cdot OEt_2$ (1.24 mL, 9.79 mmol, 6 eq.) were reacted stepwise in CH_2Cl_2. The crude product was purified by flash column chromatography; column 1: (Ø 8 cm, h = 35 cm, V = 60 mL, CH_2Cl_2: CH_3OH = 97:3); column 2: (Ø 8 cm, h = 35 cm, V = 20 mL, CH_2Cl_2: CH_3OH = 99:1), column 3: (Ø 8 cm, h = 5 cm, V = 20 mL, CH_2Cl_2: CH_3OH = 99:1, without compressed air), automatic flash column chromatography (cartridge: SNAP C18, 30g (Biotage®), 50% → 100% CH_3CN in H_2O, 25 mL/min, V = 20 mL), R_f = 0.46 (cyclohexane: ethyl acetate = 1:4). Orange oli, yield 236 mg (27 %), $C_{27}H_{34}BF_2N_5O_4$ (M_r = 541.4. Purity (HLPC, method 1): 94.6 % (t_R = 24.0 min). HR-MS (ESI): (m/z) = 542.2742 (calcd. 542.2750 for $C_{27}H_{35}BF_2N_5O_4$ $[M+H]^+$). ^1H NMR (600 MHz, $CDCl_3$): δ (ppm) = 1.42 (s, 6H, 1-C*H*3, 7-C*H*3 (indacene)), 2.55 (s, 6H, 3-C*H*3, 5-C*H*3 (indacene)), 3.39 (t, *J* = 5.0 Hz, 2H, 12-C*H*2 (dodecyl)), 3.67 – 3.71 (m, 6H, 11-C*H*2, 9-C*H*2, 8-C*H*2 (dodecyl)), 3.71 – 3.74 (m, 2H, 6-C*H*2 (dodecyl)), 3.74 – 3.78 (m, 2H, 5-C*H*2 (dodecyl)), 3.91 (t, *J* = 4.9 Hz, 2H, 3-C*H*2 (dodecyl)), 4.19 (t, *J* = 5.0 Hz, 2H, 2-C*H*2 (dodecyl)), 5.97 (s, 2H, 2-C*H*, 6-C*H* (indacene)), 7.02 (d, *J* = 8.4 Hz, 2H, 3-*H*, 5-*H* (Ph)), 7.15 (d, *J* = 8.4 Hz, 2H, 2-*H*, 6-*H* (Ph)). ^{13}C NMR (151 MHz, $CDCl_3$): δ (ppm) = 14.7 (4C, 1-CH_3, 3-CH_3, 5-CH_3, 7-CH_3 (indacene)), 50.8 (1C, C-12 (dodecyl)), 67.6 1C, C-2 (dodecyl)), 69.9 (1C, C-3 (dodecyl)), 70.2, 70.8, 70.9, 71.05 (5C, C-5, C-6, C-8, C-9, C-11 (dodecyl)), 115.3 (2C, C-3, C-5 (Ph)), 121.2 (2C, C-2, C-6 (indacene)), 127.4 (1C, C-8 (indacene)), 129.3 (2C, C-2, C-6 (Ph)), 132.0 (2C, C-7a, C-8a (indacene)), 142.0 (1C, C-4 (Ph)), 143.3 (2C, C-1, C-7 (indacene)), 155.4 (2C, C-3, C-5 (indacene)), 159.5 (1C, C-1 (Ph)). ^{11}B NMR (128 MHz, $CDCl_3$) δ (ppm) = 0.62 (t, *J* = 30.1 Hz). ^{19}F NMR (376 MHz, $CDCl_3$): δ (ppm) = -148.47 (q, *J* = 29.7 Hz). UV absorption: (CH_3CN): λ_{max} = 497 nm, (CH_3Cl/CH_3OH):

λ_{max} = 500 nm, Fluorescence emission: (CH_3CN): λ_{max} = 508 nm, (CH_3Cl/CH_3OH): λ_{max} = 510 nm, Quantum yield (CH_3Cl/CH_3OH): rt: 5 ± 2 %, 77K: 12 ± 2 %.

Synthesis of BODIPY-labeled ligands 21-26

2-{4-[(1-{2-[4-(10,10-Difluoro-4,6-dimethyldithieno[2,3-b:3',2'-g]-4-bora-3a,4a-diaza-s-indacen-8-yl)phenoxy]ethyl}-1,2,3-triazol-4-yl)methoxy]phenyl}-2-(4-flluorophenyl)-2-phenylacetamide (21)

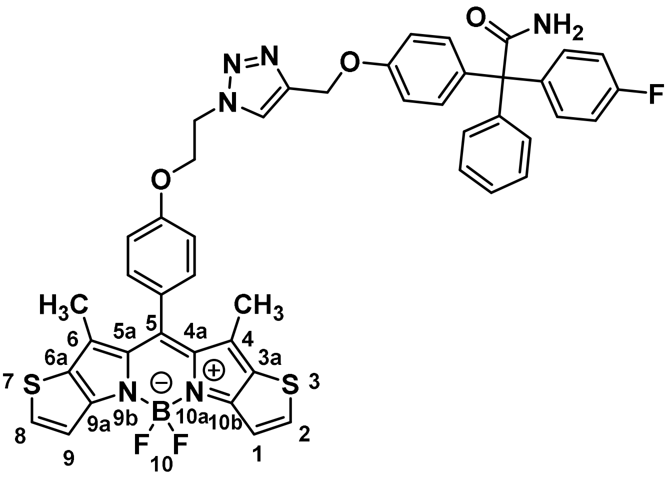

BODIPY dye **15** (37.3 mg, 0.08 mmol, 1.0 eq.) and propargyl ether **8** (29.9 mg, 0.08 mmol, 1.0 eq.) were dissolved in DMF (2.5 mL) and H_2O (2.5 mL). Sodium ascorbate (98.8 mg, 0.50 mmol, 6.6 eq.) and $CuSO_4$ (78.4 mg, 0.49 mmol, 6.5 eq.) were added. The mixture was stirred at room temperature for 24 h. LiCl solution (5 % wt in H_2O, 5 mL) and CH_2Cl_2 (5 mL) were added. The organic layer was separated and washed with LiCl solution (5 % wt in H_2O, 3 x 5 mL). The organic layer was dried (Na_2SO_4), filtered and concentrated *in vacuo*. The crude product was purified by flash column chromatography; column 1: (Ø = 4 cm, h = 30 cm, V = 10 mL, cyclohexane: ethyl acetate = 2:1 → ethyl acetate), column 2: (Ø = 2 cm, h = 35 cm, V = 5 mL, cyclohexane: ethyl acetate = 2:1 → ethyl acetate), R_f = 0.35 (cyclohexane: ethyl acetate = 2:1). Purple solid, mp = 114 °C, yield 37.1 mg (58 %), $C_{46}H_{36}BF_3N_6O_3S_2$ (M_r = 852.8). Purity (HLPC, method 2): 95.6 % (t_R = 19.1 min). HR-MS (ESI): (m/z) = 833.2345 (calcd. 833.2354 for $C_{46}H_{36}BF_2N_6O_3S_2$ $[M-F]^+$). ^1H NMR (600 MHz, $CDCl_3$): δ (ppm) = 1.60 (s, 6H, 4-CH_3, 6-CH_3), 4.48 (t, *J* = 5.0 Hz, 2H, OCH$_2$C*H*$_2$N), 4.85 (t, *J* = 5.0 Hz, 2H, OC*H*$_2$CH$_2$N), 5.24 (s, 2H, arylC*H*$_2$O), 5.75 (bs, 1H, N*H*$_2$), 5.86 (bs, 1H, N*H*$_2$), 6.93 – 7.00 (m, 4H, 3-*H*, 5-*H* $_{(FPh)}$, 3-*H*, 5-*H* $_{(OPh)}$), 7.04 (d, *J* = 8.7 Hz, 2H, 3-*H*, 5-*H* $_{(dye\text{-}Ph)}$), 7.15 (d, *J* = 5.3 Hz, 2H, 2-*H*, 8-*H* $_{(thieno)}$), 7.20 (d,

J = 8.9 Hz, 2H, 2-H, 6-H (OPh)), 7.22 – 7.25 (m, 2H, 2-H, 6-H (Ph)), 7.26 – 7.33 (m, 7H, 2-H, 6-H (FPh), 3-H, 4-H, 5-H (Ph), 2-H, 6-H (dye-Ph)), 7.60 (d, J = 5.3 Hz, 2H, 1-H, 9-H (thieno)), 7.87 (s, 1H, CH (triazole)). ^{13}C NMR (151 MHz, $CDCl_3$): δ (ppm) = 14.5 (2C, 4-CH_3, 6-CH_3), 50.0 (1C, OCH_2CH_2N), 62.2 (1C, arylCH_2O), 66.5 (1C, OCH_2CH_2N), 66.5 (1C, CCONH$_2$), 114.3 (2C, C-3, C-5 (OPh)), 114.3 (2C, C-2, C-8 (thieno)), 114.9 (d, J = 21.0 Hz, 2C, C-3, C-5 (FPh)), 115.5 (2C, C-3, C-5 (dye-Ph)), 124.3 (1C, C-5 (triazole)), 127.3 (1C, C-5 (indacene)), 127.5 (1C, C-4 (Ph)), 128.3 (2C, C-3, C-5 (Ph)), 130.0 (2C, C-2, C-6 (dye-Ph)), 130.3 (2C, C-2,C-6 (Ph)), 131.7 (2C, C-2, C-6 (OPh)), 132.2 (d, J = 7.6 Hz, 2C, C-2, C-6 (FPh)), 133.9 (2C, C-1, C-9 (thieno)), 134.0 (2C, C-4a, C-5a (indacene)), 136.0 (1C, C-1 (OPh)), 138.0 (2C, C-4, C-6 (indacene)), 139.2 (d, J = 3.4 Hz, 1C, C-1 (FPh)), 140.5 (1C, C-1 (Ph)), 143.4 (1C, C-1 (Ph)), 144.3 (1C, C-4 (triazole)), 145.4 (2C, C-4a, C-6a (indacene)), 157.2 (2C, C-9a, C-10b (indacene)), 157.4 (1C, C-4 (OPh)), 158.9 (1C, C-4 (dye-Ph)), 161.8 (d, J = 247.3 Hz, 1C, C-4 (FPh)), 175.9 (1C, CONH$_2$). UV absorption (CH_3CN): λ_{max} = 554, Fluorescence emission (CH_3CN): λ_{max} = 568 nm.

2-{4-[(1-{2-[4-(2,6-Diethyl-4,4-difluoro-1,3,5,7-tetramethyl-4-bora-3a,4a-diaza-s-indacen-8-yl)phenoxy]ethyl}-1,2,3-triazol-4-yl)methoxy]phenyl}-2-(4-flluorophenyl)-2-phenylacetamide (22)

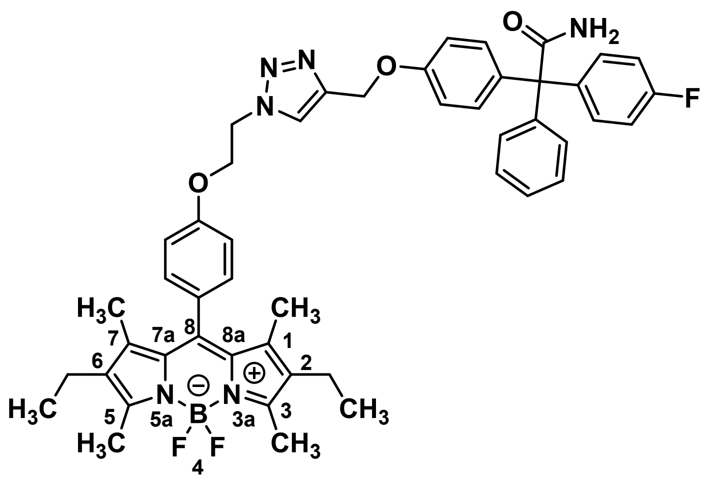

BODIPY dye **16** (100 mg, 0.22 mmol, 1.0 eq.) and propargyl ether **8** (78.0 mg, 0.22 mmol, 1.0 eq.) were dissolved in DMF (17 mL) and H_2O (20 mL). Sodium ascorbate (281 mg, 1.42 mmol, 6.6 eq.) and $CuSO_4$ (223 mg, 1.39 mmol, 6.5 eq.) were added. The mixture was stirred at room temperature for 24 h. LiCl solution (5 % wt in H_2O, 30 mL) and CH_2Cl_2 (35 mL) were added. The organic layer was separated and washed with LiCl solution (5 % wt in H_2O, 3 x 30 mL). The organic layer was dried

(Na_2SO_4), filtered and concentrated *in vacuo*. The crude product was purified by flash column chromatography (Ø = 8 cm, h = 30 cm, V = 20 mL, cyclohexane: ethyl acetate = 2:8), automatic flash column chromatography 1: (cartridge: SNAP C18, 30g (Biotage®), 70% → 100% CH_3CN in water, 25 mL/min), automatic flash column chromatography 2: (cartridge: SNAP C18, 30g (Biotage®), 80 % → 100 % CH_3CN in water, 25 mL/min), and semi- preparative HPLC (method C), R_f = 0.26 (cyclohexane: ethyl acetate 2:8). Orange solid, mp = 127 °C (lyophilisate), yield 100 mg (57 %), $C_{48}H_{48}BF_3N_6O_3$ (M_r = 824.8). Purity (HLPC, method 2): 98.7 % (t_R = 20.0 min). HR-MS (ESI): (m/z) = 805.3907 (calcd. 805.3852 for $C_{48}H_{48}BF_2N_6O_3$ $[M-F]^+$). ^{1}H NMR (600 MHz, CD_2Cl_2): δ (ppm) = 0.96 (t, *J* = 7.6 Hz, 6H, 2-CH_2CH_3, 6-CH_2CH_3), 1.31 (s, 6H, 1-CH_3, 7-CH_3), 2.30 (q, *J* = 7.6 Hz, 4H, 2-CH_2CH_3, 6-CH_2CH_3), 2.47 (s, 6H, 3-CH_3, 5-CH_3), 4.44 (t, *J* = 5.1 Hz, 2H, OCH_2CH_2N), 4.80 (t, *J* = 5.0 Hz, 2H, OCH_2CH_2N), 5.18 (s, 2H, arylCH_2O), 5.72 (bs, 1H, NH_2), 5.95 (bs, 1H, NH_2), 6.92 – 7.02 (m, 6H, 3-*H*, 5-*H* (FPh), 3-*H*, 5-*H* (OPh), 3-*H*, 5-*H* (dye-Ph)), 7.14 – 7.20 (m, 4H, 2-*H*, 6-*H* (OPh), 2-*H*, 6-*H* (dye-Ph)), 7.21 – 7.24 (m, 2H, 2-*H*, 6-*H* (Ph)), 7.25 – 7.32 (m, 5H, 2-*H*, 6-*H* (FPh), 3-*H*, 4-*H*, 5-*H* (Ph)), 7.88 (s, 1H, C*H* (triazole)). ^{13}C NMR (151 MHz, CD_2Cl_2): δ (ppm) = 12.3 (2C, 1-CH_3, 7-CH_3), 12.8 (2C, 3-CH_3, 5-CH_3), 15.0 (2C, 2-CH_2CH_3, 6-CH_2CH_3), 17.5 (2C, 2-CH_2CH_3, 6-CH_2CH_3), 50.4 (1C, OCH_2CH_2N), 62.5 (1C, arylCH_2O), 66.8 (1C, *C*$CONH_2$), 67.0 (1C, OCH_2CH_2N), 114.6 (2C, C-3, C-5 (OPh)), 115.0 (d, *J* = 21.4 Hz, C-3, C-5 (FPh)), 115.6 (2C, C-3, C-5 (dye-Ph)), 124.7 (1C, C-5 (triazole)), 127.7 (1C, C-4 (Ph)), 128.6 (2C, C-3, C-5 (Ph)), 129.2 (1C, C-8 (indacene)), 130.3 (2C, C-2, C-6 (dye-Ph)), 130.8 (2C, C-2,C-6 (Ph)), 131.5 (2C, C-7a, C-8a (indacene)), 132.1 (2C, C-2, C-6 (OPh)), 132.7 (d, *J* = 8.0 Hz, 2C, C-2, C-6 (FPh)), 133.4 (2C, C-2, C-6 (indacene)), 136.4 (1C, C-1 (OPh)), 139.1 (2C, C-1, C-7 (indacene)), 140.0 (d, *J* = 3.5 Hz, 1C, C-1 (FPh)), 140.6 (1C, C-1 (dye-Ph)), 144.0 (1C, C-1 (Ph)), 144.4 (1C, C-4 (triazole)), 154.0 (2C, C-3, C-5 (indacene)), 157.9 (1C, C-4 (OPh)), 159.0 (1C, C-4 (dye-Ph)), 162.2 (d, *J* = 246.3 Hz, 1C, C-4 (FPh)), 175.9 (1C, *C*ONH_2). UV absorption (CH_3CN): λ_{max} = 521 nm, Fluorescence emission (CH_3CN): λ_{max} = 534 nm.

2-{4-[(1-{11-[4-(2,6-Diethyl-4,4-difluoro-1,3,5,7-tetramethyl-4-bora-3a,4a-diaza-s-indacen-8-yl)phenoxy]-3,6,9-trioxaundecyl}-1,2,3-triazol-4-yl)methoxy]phenyl}-2-(4-fluorophenyl)-2-phenylacetamide (23)

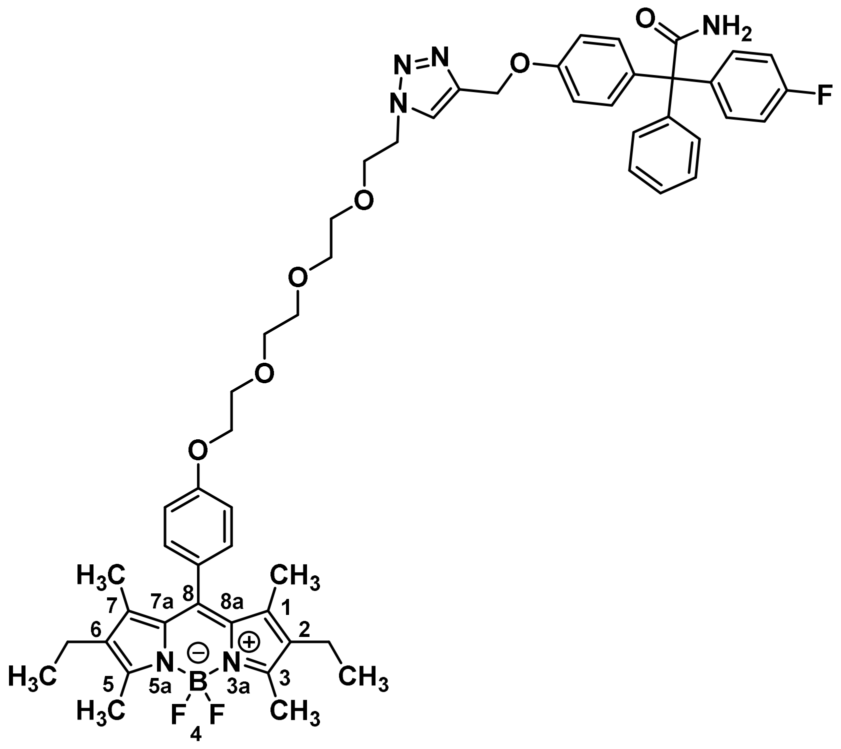

BODIPY dye **17** (100 mg, 0.17 mmol, 1.0 eq.) and propargyl ether **8** (60.0 mg, 0.17 mmol, 1.0 eq.) were dissolved in DMF (10 mL) and H_2O (20 mL). Sodium ascorbate (218 mg, 1.10 mmol, 6.6 eq.) and $CuSO_4$ (173 mg, 1.09 mmol, 6.5 eq.) were added. The mixture was stirred at room temperature for 24 h. LiCl solution (5 % wt in H_2O, 30 mL) and CH_2Cl_2 (30 mL) were added. The organic layer was separated and washed with LiCl solution (5 % wt in H_2O, 3 x 30 mL). The organic layer was dried (Na_2SO_4), filtered and concentrated *in vacuo*. The crude product was purified by flash column chromatography; column 1: (Ø = 8 cm, h = 25 cm, V = 20 mL, CH_2Cl_2: CH_3OH = 9:1), column 2: (Ø = 6 cm, h = 35 cm, V = 15 mL, CH_2Cl_2: CH_3OH = 20:1), automatic flash column chromatography: (cartridge: SNAP C18, 30g (Biotage®), 70 % → 100 % CH_3CN in water, 25 mL/min) and semi-preparative HPLC (method B), R_f = 0.37 (CH_2Cl_2: CH_3OH = 20:1). Orange solid, mp = 76 °C (lyophilisate), yield 97.5 mg (61 %), $C_{54}H_{60}BF_3N_6O_6$ (M_r = 956.9). Purity (HLPC, method 2): 99.4 % (t_R = 20.1 min). HR-MS (ESI): (m/z) = 957.4749 (calcd. 957.4701 for $C_{54}H_{61}BF_3N_6O_6$ $[M+H]^+$). ^1H NMR (600 MHz, CD_2Cl_2): δ (ppm) = 0.99 (t, J = 7.6 Hz, 6H, 2-CH_2CH_3, 6-CH_2CH_3 (indacene)), 1.35 (s, 6H, 1-CH_3, 7-CH_3 (indacene)), 2.32 (q, J = 7.6 Hz, 4H, 2-CH_2CH_3, 6-CH_2CH_3 (indacene)), 2.48 (s, 6H, 3-CH_3, 5-CH_3 (indacene)), 3.56 – 3.65 (m, 6H, 1-CH_2, 2-CH_2, 4-

C*H*$_2$ (dodecyl)), 3.65 – 3.70 (m, 2H, 5-C*H*$_2$ (dodecyl)), 3.82 (t, *J* = 4.9 Hz, 2H, 7-C*H*$_2$ (dodecyl)), 3.88 (t, *J* = 4.6 Hz, 2H, 10-C*H*$_2$ (dodecyl)), 4.14 (t, *J* = 4.5 Hz, 2H, 8-C*H*$_2$ (dodecyl)), 4.55 (t, *J* = 4.9 Hz, 2H, 11-C*H*$_2$ (dodecyl)), 5.17 (s, 2H, arylC*H*$_2$O), 5.73 (bs, 1H, N*H*$_2$), 6.00 (bs, 1H, N*H*$_2$), 6.94 – 7.04 (m, 6H, 3-*H*, 5-*H* (FPh), 3-*H*, 5-*H* (OPh), 3-*H*, 5-*H* (dye-Ph)), 7.15 – 7.20 (m, 4H, 2-*H*, 6-*H* (OPh), 2-*H*, 6-*H* (dye-Ph)), 7.23 – 7.26 (m, 2H, 2-*H*, 6-*H* (Ph)), 7.26 – 7.33 (m, 5H, 2-*H*, 6-*H* (FPh), 3-*H*, 4-*H*, 5-*H* (Ph)), 7.87 (s, 1H, C*H* (triazole)). ^{13}C NMR (151 MHz, CD_2Cl_2): δ (ppm) = 12.2 (2C, 1-CH_3, 7-CH_3 (indacene)), 12.8 (2C, 3-CH_3, 5-CH_3 (indacene)), 15.0 (2C, 2-CH_2*C*H_3, 6-CH_2*C*H_3 (indacene)), 17.5 (2C, 2-*C*H_2CH_3, 6-*C*H_2CH_3 (indacene)), 50.9 (1C, C-11 (dodecyl)), 62.5 1C, aryl*C*H_2O), 66.8 (1C, *C*CONH_2), 68.1 (1C, C-8 (dodecyl)), 69.9 (1C, C-10 (dodecyl)), 70.2 (1C, C-7 (dodecyl)), 71.0 (1C, C-4 (dodecyl)), 71.1, 71.1 (2C, C-1, C-2 (dodecyl)), 71.3 (1C, C-5 (dodecyl)), 114.7 (2C, C-3, C-5 (OPh)), 115.0 (d, *J* = 20.9 Hz, C-3, C-5 (FPh)), 115.5 (2C, C-3, C-5 (dye-Ph)), 124.7 (1C, C-5 (triazole)), 127.7 (1C, C-4 (Ph)), 128.4 (2C, C-3, C-5 (Ph)), 128.6 (1C, C-8 (indacene)), 130.1 (2C, C-2, C-6 (dye-Ph)), 130.8 (2C, C-2,C-6 (Ph)), 131.6 (2C, C-1a, C-7a (indacene)), 132.1 (2C, C-2, C-6 (OPh)), 132.7 (d, *J* = 8.1 Hz, C-2, C-6 (FPh)), 133.4 (2C, C-2, C-6 (indacene)), 136.3 (1C, C-1 (OPh)) , 139.2 (2C, C-1, C-7 (indacene)), 140.1 (d, *J* = 3.3 Hz, C-1 (FPh)), 141.0 (1C, C-1 (dye-Ph)), 144.0 (1C, C-1 (Ph)), 144.1 (1C, C-4 (triazole)), 154.0 (2C, C-3, C-5 (indacene)), 158.0 (1C, C-4 (OPh)), 159.8 (1C, C-4 (dye-Ph)), 162.2 (d, *J* = 246.2 Hz, C-4 (FPh)), 175.9 (1C, *C*ONH_2). UV absorption (CH_3CN): λ_{max} = 521, Fluorescence emission (CH_3CN): λ_{max} = 534 nm.

2-{4-[(1-{12-[4-(2,6-Diethyl-4,4-difluoro-1,3,5,7-tetramethyl-4-bora-3a,4a-diaza-s-indacen-8-yl)phenoxy]dodecyl}-1,2,3-triazol-4-yl)methoxy]phenyl}-2-(4-fluorophenyl)-2-phenylacetamide (24)

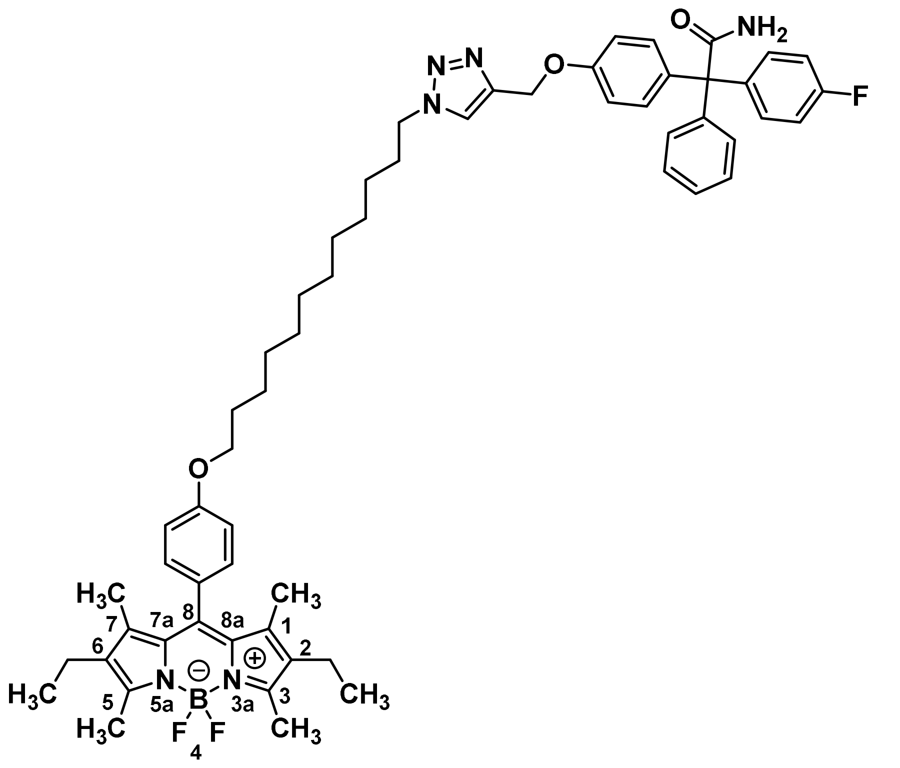

BODIPY dye **18** (100 mg, 0.17 mmol, 1.0 eq.) and propargyl ether **8** (60.0 mg, 0.17 mmol, 1.0 eq.) were dissolved in DMF (15 mL) and H_2O (20 mL). Sodium ascorbate (216 mg, 1.09 mmol, 6.6 eq.) and $CuSO_4$ (171 mg, 1.07 mmol, 6.5 eq.) were added. The mixture was stirred at room temperature for 24 h. LiCl solution (5 % wt in H_2O, 35 mL) and CH_2Cl_2 (35 mL) were added. The organic layer was separated and washed with LiCl solution (5 % wt in H_2O, 3 x 35 mL). The organic layer was dried (Na_2SO_4), filtered and concentrated *in vacuo*. The crude product was purified by flash column chromatography (Ø = 8 cm, h = 20 cm, V = 20 mL, cyclohexane: ethyl acetate = 4:1 → ethyl acetate), R_f = 0.48 (cyclohexane: ethyl acetate = 6:1). Orange oil, yield 3.3 mg (2 %), $C_{58}H_{68}BF_3N_6O_3$ (M_r = 965.0). Purity (HLPC, method 2): % (t_R = 98.2 min). HR-MS (ESI): (m/z) = (calcd. for $C_{58}H_{69}BF_3N_6O_3$ $[M+H]^+$). ^1H NMR (600 MHz, CD_3Cl): δ (ppm) = 0.98 (t, *J* = 7.5 Hz, 4H, 2-$CH_2C\mathit{H}_3$, 6-$CH_2C\mathit{H}_3$ (indacene))*, 1.27 – 1.29 (m, 8H, 4-$C\mathit{H}_2$, 5-$C\mathit{H}_2$, 6-$C\mathit{H}_2$, 7-$C\mathit{H}_2$ (dodecyl)), 1.33 (s, 6H, 1-$C\mathit{H}_3$, 7-$C\mathit{H}_3$ (indacene)), 1.46 – 1.51 (m, 2H, 3-$C\mathit{H}_2$ (dodecyl)), 1.53 – 1.58 (m, 2H, 8-$C\mathit{H}_2$ (dodecyl)), 1.65 – 1.69 (m, 4H, 9-$C\mathit{H}_2$, 10-$C\mathit{H}_2$ (dodecyl)) 1.82 (quint., *J* = 6.7 Hz, 2H, 2-$C\mathit{H}_2$ (dodecyl), 1.92 (quint, *J* = 6.8 Hz, 2H, 11-$C\mathit{H}_2$ (dodecyl)), 2.27 – 2.34 (m, 4H, 2-$C\mathit{H}_2CH_3$, 6-$C\mathit{H}_2CH_3$ (indacene)), 2.52 (s, 4H, 3-$C\mathit{H}_3$,

5-CH_3 (indacene))*, 3.96 – 4.00 (m, 2H, 1-CH_2 (dodecyl)), 4.36 (t, *J* = 7.3 Hz, 2H, 12-CH_2 (dodecyl)), 5.21 (s, 2H, arylCH_2O), 5.74 (bs, 2H, NH_2), 6.94 (d, *J* = 8.9 Hz, 2H, 3-*H*, 5-*H* (OPh)), 6.96 – 7.02 (m, 4H, 3-*H*, 5-*H* (FPh), 3-*H*, 5-*H* (dye-Ph)), 7.11 – 7.16 (m, 2H, 2-*H*, 6-*H* (dye-Ph)), 7.18 (d, *J* = 8.9 Hz, 2H, 2-*H*, 6-*H* (OPh)), 7.22 – 7.25 (m, 2H, 2-*H*, 6-*H* (Ph)), 7.26 – 7.34 (m, 5H, 2-*H*, 6-*H* (FPh), 3-*H*, 4-*H*, 5-*H* (Ph)), 7.60 (s, 1H, C*H* (triazole)), * two protons cannot be seen in this signal, ^{13}C NMR (151 MHz, CD_3Cl): δ (ppm) = 11.1 (2C, 1-CH_3, 7-CH_3 (indacene)), 12.0 (2C, 3-CH_3, 5-CH_3 (indacene)), 14.8 (2C, 2-CH_2*C*H_3, 6-CH_2*C*H_3 (indacene)), 17.23 (2C, 2-*C*H_2CH_3, 6-*C*H_2CH_3 (indacene)), 23.9, 24.6, 26.2, 26.7, 29.1, 29.2, 29.4, 29.5, 29.6, 29.7 (10C, C-12 (dodecyl)), 50.7 (1C, C-12 (dodecyl)), 62.2 (1C, aryl*C*H_2O), 67.0 (1C, *C*CONH_2), 68.3 (1C, C-1 (dodecyl)), 114.4 (2C, C-3, C-5 (OPh)), 114.9 (d, *J* = 21.0 Hz, 2C, C-3, C-5 (FPh)), 115.1 (2C, C-3, C-5 (dye-Ph)), 127.5 (1C, C-4 (Ph)), 127.8 (1C, C-5 (triazole)), 128.3 (2C, C-3, C-5 (Ph)), 129.4 (1C, C-8 (indacene)), 129.6 (2C, C-2, C-6 (dye-Ph)), 130.3 (2C, C-2,C-6 (Ph)), 131.4 (2C, C-1a, C-7a (indacene)), 131.7 (2C, C-2, C-6 (OPh)), 132.2 (d, *J* = 7.9 Hz, 2C, C-2, C-6 (FPh)), , 132.8 (2C, C-2, C-6 (indacene)), 135.9 (1C, C-1 (OPh)), 138.6 (2C, C-1, C-7 (indacene)), 139.3 (d, *J* = 3.0 Hz, 1C, C-1 (FPh)), 140.5 (1C, C-1 (dye-Ph)), 143.4 (2C, C-1 (Ph), C-4 (triazole)), 153.6 (2C, C-3, C-5 (indacene)), 157.4 (1C, C-4 (OPh)), 159.7 (1C, C-4 (dye-Ph)), 161.8 (d, *J* = 246.9 Hz, 1C, C-4 (FPh)), 173.7 (1C, *C*ONH_2). UV absorption (CH_3CN): λ_{max} = 521 nm, Fluorescence emission (CH_3CN): λ_{max} = 533 nm.

2-{4-[(1-{2-[4-(4,4-Difluoro-1,3,5,7-tetramethyl-4-bora-3a,4a-diaza-s-indacen-8-yl)phenoxy]ethyl}-1,2,3-triazol-4-yl)methoxy]phenyl}-2-(4-flluorophenyl)-2-phenylacetamide (25)

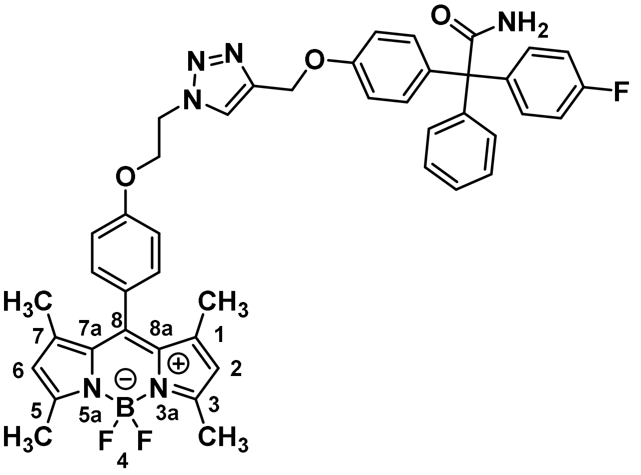

BODIPY dye **19** (167 mg, 0.40 mmol,1.0 eq.) and propargyl ether **8** (144 mg, 0.40 mmol, 1.0 eq.) were dissolved in DMF (15 mL) and H_2O (15 mL). Sodium ascorbate (526 mg, 2.65 mmol, 6.6 eq.) and $CuSO_4$ (417 mg, 2.61 mmol, 6.5 eq.) were added. The mixture was stirred at room temperature for 24 h. LiCl solution (5 % wt in H_2O, 20 mL) and CH_2Cl_2 (10 mL) were added. The organic layer was separated and washed with LiCl solution (5 % wt in H_2O, 3 x 20 mL). The organic layer was dried (Na_2SO_4), filtered and concentrated *in vacuo*. The crude product was purified by flash column chromatography (Ø = 6 cm, h = 30 cm, V = 20 mL, cyclohexane: ethyl acetate = 2:8 → ethyl acetate), R_f = 0.36 (cyclohexane: ethyl acetate = 3:8) and semi-preparative HPLC (method D and E). Orange solid, mp = 116 °C (lyophilisate), yield 59.3 mg (19 %), $C_{44}H_{40}BF_3N_6O_3$ (M_r = 768.7). Purity (HLPC, method 2): 97.7 % (t_R = 18.9 min). HR-MS (ESI): (m/z) = 749.3223 (calcd. 749.3225 for $C_{44}H_{40}BF2N_6O_3$ [M-F]$^+$). ^{1}H NMR (600 MHz, $CDCl_3$): δ (ppm) = 1.38 (s, 6H, 1-C*H*3, 7-C*H*3), 2.53 (s, 6H, 3-C*H*3, 5-C*H*3), 4.43 (t, *J* = 5.0 Hz, 2H, OCH_2CH_2N), 4.83 (t, *J* = 5.0 Hz, 2H, OCH_2CH_2N), 5.23 (s, 2H, arylC*H*2O), 5.74 (bs, 1H, N*H*2), 5.80 (bs, 1H, N*H*2), 5.96 (s, 2H, 2-C*H*, 6-C*H* (indacene)), 6.86 – 7.05 (m, 6H, 3-*H*, 5-*H* (FPh), 3-*H*, 5-*H* (OPh), 3-*H*, 5-*H* (dye-Ph)), 7.09 – 7.20 (m, 4H, 2-*H*, 6-*H* (OPh), 2-*H*, 6-*H* (dye-Ph)), 7.20 – 7.25 (m, 2H, 2-*H*, 6-*H* (Ph)), 7.25 – 7.34 (m, 5H, 2-*H*, 6-*H* (FPh), 3-*H*, 4-*H*, 5-*H* (Ph)), 7.86 (s, 1H, C*H* (triazole)). ^{13}C NMR (151 MHz, $CDCl_3$): δ (ppm) = 14.8 (4C, 1-CH3, 3-CH3, 5-CH3, 7-CH3), 50.1 (1C, $OCH_2CH_2N_3$), 62.0 (1C, arylCH2O), 66.4 (1C, $OCH_2CH_2N_3$), 66.5 (1C, *C*CONH2), 114.4 (2C, C-3, C-5 (OPh)), 114.9 (d, *J* = 21.3 Hz, 2C, C-3, C-5 (FPh)), 115.2 (2C, C-3,

C-5 (dye-Ph)), 121.4 (2C, C-2, C-6 (indacene)), 124.3 (1C, C-5 (triazole)), 127.5 (1C, C-4 (Ph)), 128.4 (2C, C-3, C-5 (Ph)), 128.5 (1C, C-8 (indacene)), 129.7 (2C, C-2, C-6 (dye-Ph)), 130.3 (2C, C-2,C-6 (Ph)), 131.7 (2C, C-2, C-6 (OPh)), 131.9 (2C, C-7a, C-8a (indacene)) , 132.2 (d, J = 8.0 Hz, 2C, C-2, C-6 (FPh)), 136.0 (1C, C-1 (OPh)), 139.3 (d, J = 3.4 Hz, 1C, C-1 (FPh)), 141.3 (1C, C-1 (dye-Ph)), 143.1 (2C, C-1, C-7 (indacene)), 143.4 (1C, C-1 (Ph)), 144.2 (1C, C-4 (triazole)), 155.6 (2C, C-3, C-5 (indacene)), 157.3 (1C, C-4 (OPh)), 158.4 (1C, C-4 (dye-Ph)), 161.8 (d, J = 246.9 Hz, 1C, C-4 (FPh)), 175.9 (1C, *C*ONH2). UV absorption (CH_3CN): λ_{max} = 497 nm, Fluorescence emission (CH_3CN): λ_{max} = 508 nm.

2-{4-[(1-{11-[4-(4,4-Difluoro-1,3,5,7-tetramethyl-4-bora-3a,4a-diaza-s-indacen-8-yl)phenoxy]3,6,9-trioxaundecyl}-1,2,3-triazol-4-yl)methoxy]phenyl}-2-(4-flluorophenyl)-2-phenylacetamide (26)

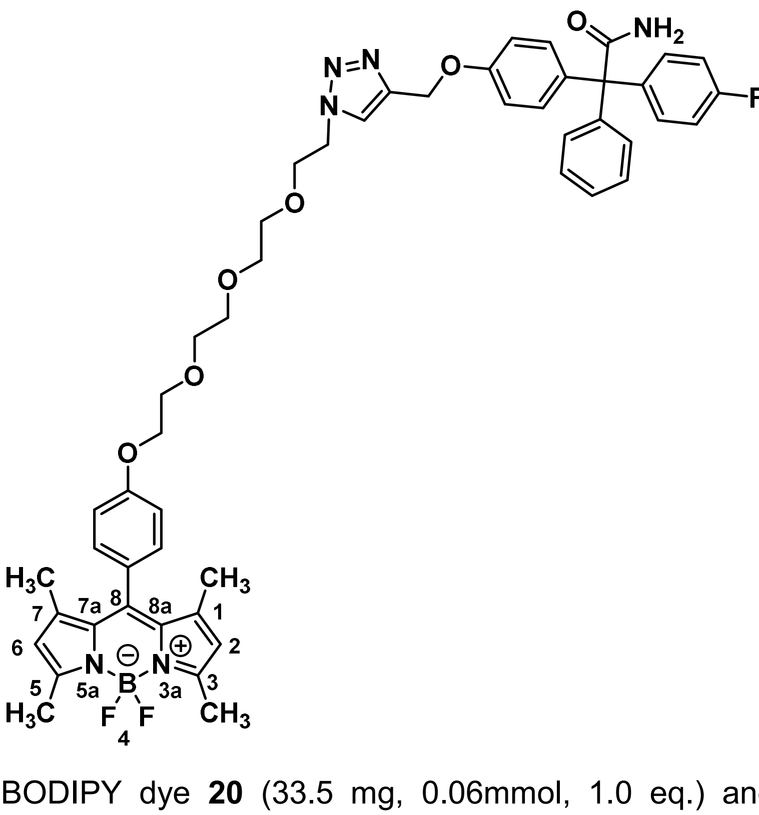

BODIPY dye **20** (33.5 mg, 0.06mmol, 1.0 eq.) and propargyl ether **8** (22.2 mg, 0.06 mmol, 1.0 eq.) were dissolved in DMF (1.0 mL) and H_2O (0.8 mL). Sodium ascorbate (80.9 mg, 0.41 mmol, 6.6 eq.) and $CuSO_4$ (64.2 mg, 0.40 mmol, 6.5 eq.) were added. The mixture was stirred at room temperature for 24 h. LiCl solution (5 % wt in H_2O, 5 mL) and CH_2Cl_2 (5 mL) were added. The organic layer was separated and washed with LiCl solution (5 % wt in H_2O, 3 x 5 mL). The organic layer was dried (Na_2SO_4), filtered and concentrated *in vacuo*. The crude product was purified by flash column chromatography (Ø = 5 cm, h = 20 cm, V = 5 mL, ethyl acetate → ethyl acetate:

CH_3OH = 10:1), automatic flash column chromatography 1: (cartridge: SNAP C18, 30g (Biotage®), 33% → 100% CH_3CN in water, 25 mL/min), automatic flash column chromatography 2: (cartridge: SNAP C18, 30g (Biotage®), 40% → 100% CH_3CN in water, 25 mL/min), automatic flash column chromatography 3: (cartridge: SNAP C18, 30g (Biotage®), 40% → 100% CH_3CN in water, 25 mL/min), automatic flash column chromatography 4: (cartridge: SNAP C18, 30g (Biotage®), 40% → 100% CH_3CN in water, 25 mL/min), R_f = 0.17 (ethyl acetate). Orange oil, yield 37.8 mg (68 %), $C_{50}H_{52}BF_3N_6O_6$ (M_r = 900.8). Purity (HLPC, method 1): 94.8 % (t_R = 24.4 min). HR-MS (ESI): (m/z) = 901.4061 (calcd. 901.4075 for $C_{50}H_{53}BF_3N_6O_6$ $[M+H]^+$). ^1H NMR (600 MHz, $CDCl_3$): δ (ppm) = 1.40 (s, 6H, 1-C*H*3, 7-C*H*3 (indacene)), 2.54 (s, 6H, 3-C*H*3, 5-C*H*3 (indacene)), 3.62 (s, 4H, 1-C*H*2, 2-C*H*2 (dodecyl)), 3.63 – 3.67 (m, 2H, 4-C*H*2 (dodecyl)), 3.70 – 3.75 (m, 2H, 5-C*H*2 (dodecyl)), 3.85 (t, *J* = 4.8 Hz, 2H, 7-C*H*2 (dodecyl)), 3.89 (t, *J* = 4.9 Hz, 2H, 10-C*H*2 (dodecyl)), 4.14 (t, *J* = 4.7 Hz, 2H, 8-C*H*2 (dodecyl)), 4.55 (t, *J* = 4.8 Hz, 2H, 11-C*H*2 (dodecyl)), 5.19 (s, 2H, arylC*H*2O), 5.74 (bs, 1H, N*H*2), 5.96 (s, 2H, 2-C*H*, 6-C*H* (indacene)), 5.99 (bs, 1H, N*H*2), 6.90 – 7.01 (m, 6H, 3-*H*, 5-*H* (FPh), 3-*H*, 5-*H* (OPh), 3-*H*, 5-*H* (dye-Ph)), 7.13 (d, *J* = 8.8 Hz, 2H, 2-*H*, 6-*H* (dye-Ph)), 7.17 (d, *J* = 8.8 Hz, 2H, 2-*H*, 6-*H* (OPh)), 7.21 – 7.24 (m, 2H, 2-*H*, 6-*H* (Ph)), 7.25 – 7.31 (m, 5H, 2-*H*, 6-*H* (FPh), 3-*H*, 4-*H*, 5-*H* (Ph)), 7.84 (s, 1H, C*H* (triazole)). ^{13}C NMR (151 MHz, $CDCl_3$): δ (ppm) = 14.7 (4C, 1-CH3, 3-CH3, 5-CH3, 7-CH3 (indacene)), 50.5 (1C, C-11 (dodecyl)), 62.1 (1C, aryl*C*H2O), 66.4 (1C, *C*CONH2), 67.6 (1C, C-8 (dodecyl)), 69.5 (1C, C-10 (dodecyl)), 69.8 (1C, C-7 (dodecyl)), 70.6 (1C, C-4 (dodecyl)), 70.7, 70.7 (2C, C-1, C-2 (dodecyl)), 71.0 (1C, C-5 (dodecyl)), 114.3 (2C, C-3, C-5 (OPh)), 114.8 (d, *J* = 21.0 Hz, 2C, C-3, C-5 (FPh)), 115.3 (2C, C-3, C-5 (dye-Ph)), 121.2 (2C, C-2, C-6 (indacene)), 124.2 (1C, C-5 (triazole)), 127.4 (1C, C-4 (Ph)), 128.3 (2C, C-3, C-5 (Ph)), 128.7 (1C, C-8 (indacene)), 129.3 (2C, C-2, C-6 (dye-Ph)), 130.3 (2C, C-2, C-6 (Ph)), 131.6 (2C, C-2, C-6 (OPh)), 131.9 (2C, C-7a, C-8a (indacene)), 132.2 (d, *J* = 7.6 Hz, 2C, C-2, C-6 (FPh)), 135.8 (1C, C-1 (OPh)), 139.3 (d, *J* = 3.4 Hz, 1C, C-1 (FPh)), 141.9 (1C, C-1 (dye-Ph)), 143.2 (2C, C-1, C-7 (indacene)), 143.4 (1C, C-1 (Ph)), 143.9 (1C, C-4 (triazole)), 155.4 (2C, C-3, C-5 (indacene)), 157.4 (1C, C-4 (OPh)), 159.4 (1C, C-4 (dye-Ph)), 161.7 (d, *J* = 247.3 Hz, 1C, C-4 (FPh)), 175.9 (1C, *C*ONH2). UV absorption (CH_3CN): λ_{max} = 498 nm, Fluorescence emission (CH_3CN): λ_{max} = 507 nm.

5.3. in vitro studies

Cell culture

Cells were cultivated and prepared for the experiments in cooperation with the Institute of Physiology II by Sandra Schimmelpfennig and Sarah Sargin. The cells of both cell lines (A549-3R and HEK293) were cultured in Dulbecco's Modified Eagle's Medium (DMEM) with 4.5 g/L glucose and supplemented with 10 % fetal calf serum (FCS Superior) at 37 ° C and 5 % carbon dioxide (CO_2) in cell culture dishes (Ø = 10 cm).

For experiments with fixed cells, cover slips were coated with 0.1 % poly-L-lysine (30 min, room temperature) and washed with Dulbecco's Phosphate Buffered Saline (PBS). One cover slip was placed in each well of a 12-well-plate. DMEM (1 mL) was added to each well and approximately 100.000 cells (as suspension in DMEM) were added. The cells adhered to the cover slips and were incubated overnight at 37 °C and 5 % CO_2 for the experiments. For experiments with living cells, the cells were seeded on glass bottom dishes instead of cover slips and 2 mL DMEM was added to each dish.

Preparation of fluorescent probes

Low mg amounts of compounds were weighted in a glass vial on a XP26 Delta Range® (Mettler Toledo) scales. The amount of DMSO added to the vial was adjusted according to the desired final concentration of the DMSO stock solutions.
The staining solutions for each fluorescently labeled ligand (10 µM) were obtained by dilution of the corresponding stock solution with PBS (1:1000) in a microcentrifuge tube (Eppendorf®) and the precipitate was dissolved by using a Vortex (Scientific®) for 5 min. The senicapoc solution (30 µM) for the blocking experiments was obtained by dilution of a 30 mM stock solution with DMSO (1:1000) in a microcentrifuge tube (Eppendorf®).

Staining protocols 1-3

For each experiment, the medium was removed and the cells were washed three times with PBS. The cells were fixed with 3.5 % PFA in PBS for 30 min at room temperature. Thereafter, the cells were washed three times with PBS at room temperature and kept for 10 min in PBS supplemented with 100 mmol/L glycine and washed again three times. The cover slip with the adherent cells was carefully placed

upside down onto a drop of the respective solution on Parafilm and incubated in a dark chamber. The incubation times are given in parenthesis for each experiment. After washing five times with PBS, the cover slip was placed upside down onto an object slide for microscopy.

Protocol 1: Staining with fluorescently labeled ligands (time needed: 40 min)
According to the general staining procedure, a 30 µL drop of staining solution of the respective labeled ligand was used (Incubation: 10 min).

Protocol 2: Staining with fluorescently labeled ligands after preincubation with Senicapoc solution (time needed: 45 min)
According to the general staining procedure, a 30 µL drop of the senicapoc solution was used (Incubation: 5 min). After washing with PBS three times, the cover slip was placed on another 30 µL drop, containing 15 µL of the staining solution of the respective labeled ligand and 15 µL of the senicapoc solution (Incubation: 10 min). The cover slip was prepared for microscopy as mentioned in the general procedure.

Protocol 3: Antibody-based indirect immunofluorescence assay (time needed: 260 min)
The cells were incubated with Triton^{TM} X-100 (0.25 % Octoxinol 9 in 1 % SDS/PBS) in PBS for 10 min at room temperature. Thereafter, the cells were washed five times with PBS at room temperature. The cover slip was carefully placed upside down onto a 30 µL drop of Normal Goat Serum (10 % NGS, Na_2HPO_4 (0,01 M) und NaCl (0,15 M) in Ampuwa^{TM}) on Paraflim and incubated in a moist, dark chamber for 30 min to block the unspecific binding of the antibodies to structures of the investigated biomaterial.[21] Thereafter, the cover slips were placed upside down on a 30 µL drop of primary antibodies in Normal Goat Serum (1:300; Anti-KCNN4 antibody produced in rabbit (Sigma Aldrich, AV35098) on Parafilm and incubated for 2 h. The cells were then washed five times with PBS and incubated with Cy3-conjugated secondary antibodies in Normal Goat Serum (1:500; goat anti-rabbit IgG, 30µL drop on Parafilm) for 1 h at room temperature. At the same time, negative controls were incubated with only Cy3-conjugated secondary antibodies (1:500; goat anti-rabbit IgG). After washing five times with PBS, the cells were fixed with 3.5 % PFA in PBS for 10 min at room

temperature and washed three times with PBS. The cover slip was prepared for microscopy as mentioned in the general procedure.

Protocol 4: Staining with fluorescently labeled ligands in living cells (time needed: 10 min)

For each experiment, the medium was removed and the cells were washed three times with PBS. Thereafter, 2 mL of staining solution of the respective labeled ligand were carefully pipetted into the glas bottom dishes and incubated at room temperature for 10 min. The staining solution was removed and the cells were washed with PBS five times. 2 mL PBS were added and the glas bottom dishes were used for microscopy.

Microscopy

Microscopy was performed with an inverted microscope (Axiovert 200, Zeiss AG, Oberkochen, Germany) equipped with a x100 oil immersion objective and a digital camera (SPOT RT SE from Diagnostic Instruments Inc.). Filters were used to irradiate the fluorescent dyes with light according to their absorption maximum and to filter desired wavelengths of the emitted light. The following filter sets were used: FITC (Carl Zeiss filter set 10, bandwidth excitation filter 450 - 490, color divider 510, bandpass emission filter 515 - 565) and TRITC (Carl Zeiss Filter Set 15, Bandpass Filter 546/12, color divider 580, emission filter Long Pass 590). Signals detected in the FITC and TRITC channels are denoted as “green” and “red” fluorescence respectively. Data acquisition and analysis were performed with Metavue software (version 6.3r6 from Molecular Devices LLC, Visitron).

For the evaluation of the density of the $K_{Ca}3.1$ channel, ten squares with a side length of 50 pixels, corresponding to an area of 9 μm^2, were randomly scanned in one cell. The experiments were repeated at three different days ($n = 3$) and for each day $n = 5$ cells were analyzed. Signals with a *Full Width at Half Maximum* ≤ 5 pixels (~ 300 nm) were counted as one $K_{Ca}3.1$ channel.

6. ^{1}H and ^{13}C NMR spectra

^{1}H and ^{13}C NMR spectra of (4-Bromophenoxy)-(*tert*-butyl)diphenylsilane **(4)**[1] in $CDCl_3$.

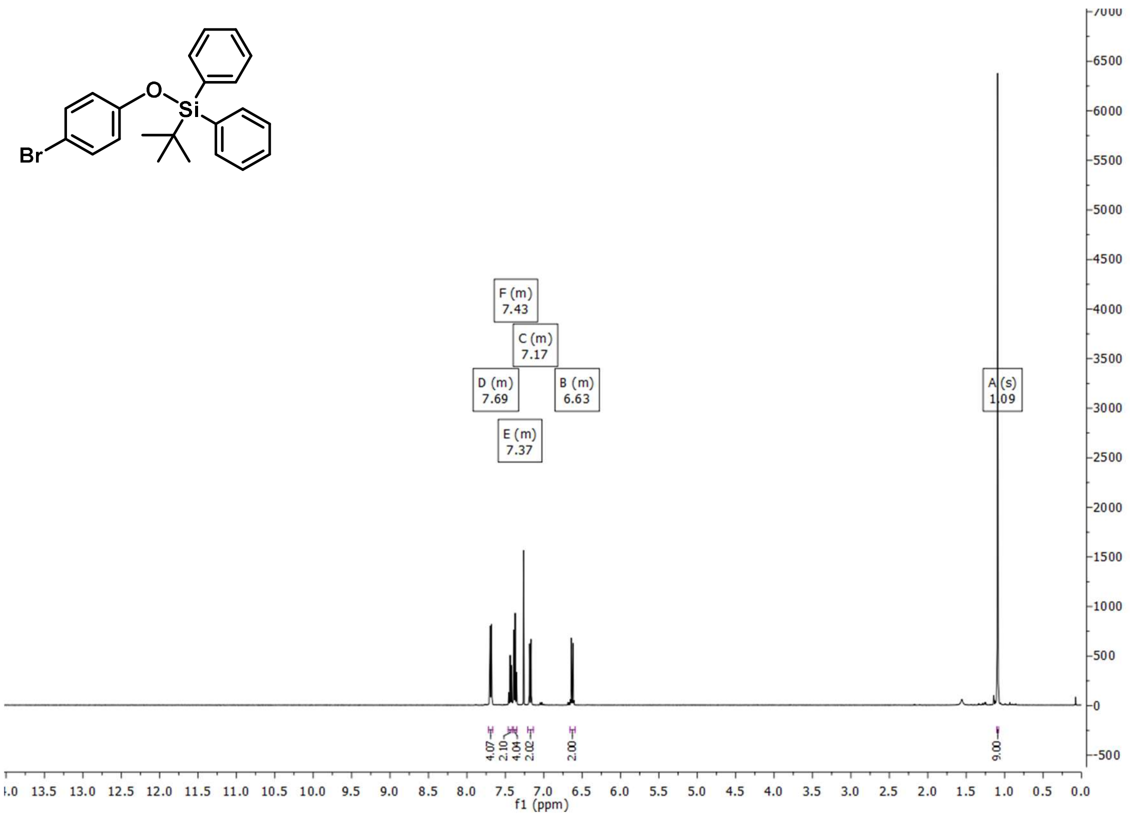

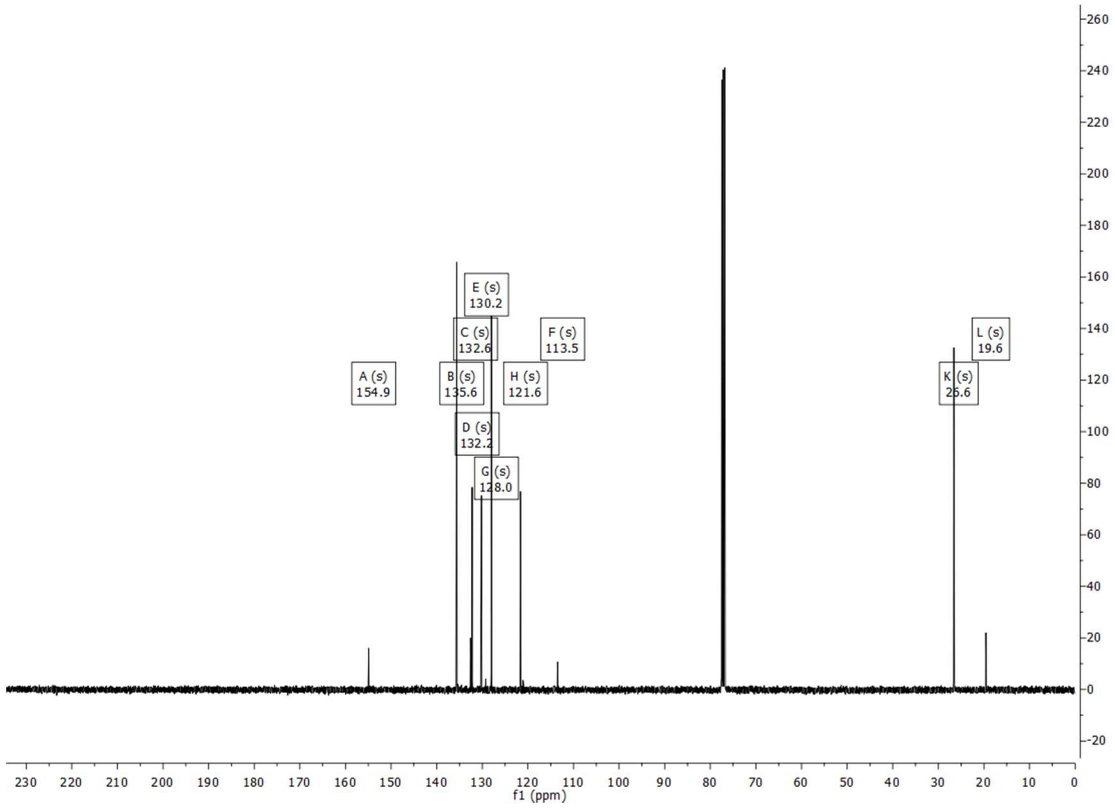

^{1}H and ^{13}C NMR spectra of {4-[(*tert*-Butyldiphenylsilyl)oxy]phenyl}(4-fluorophenyl)(phenyl)methanol **(5)** in $CDCl_3$.

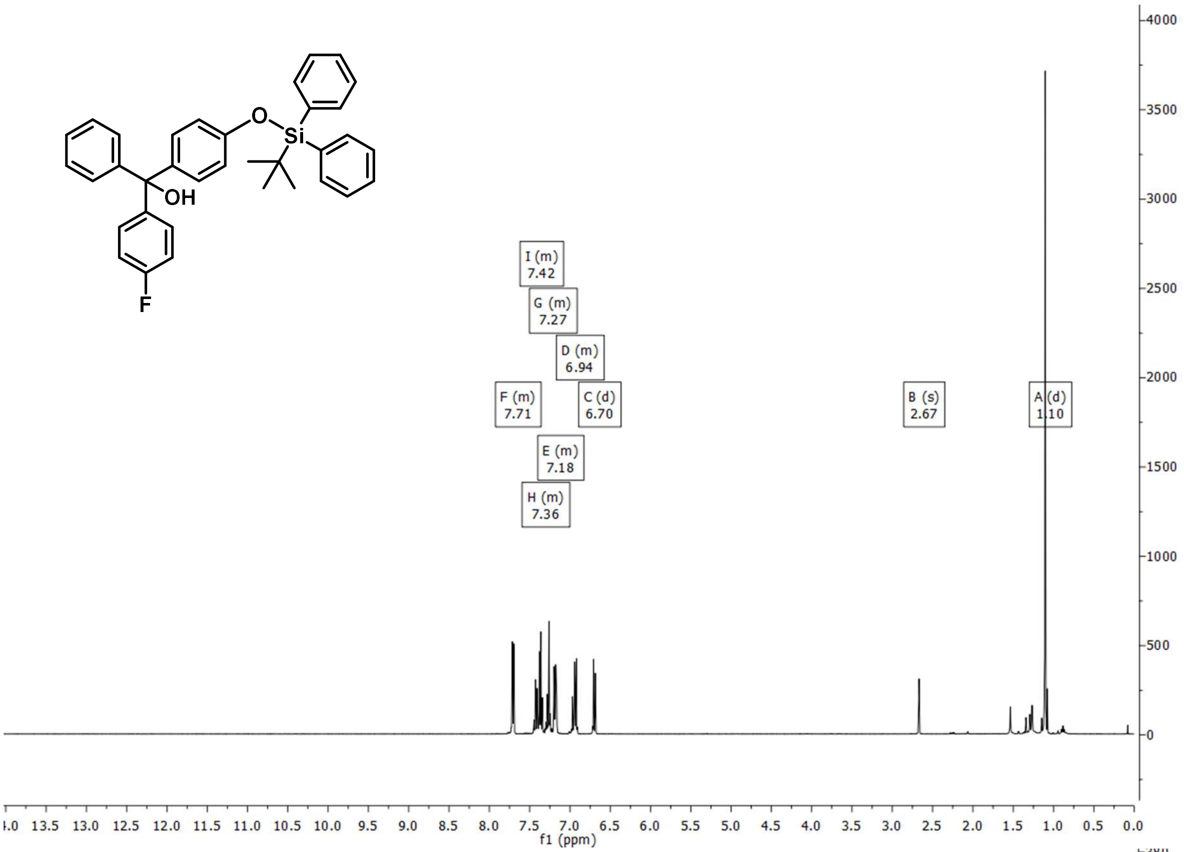

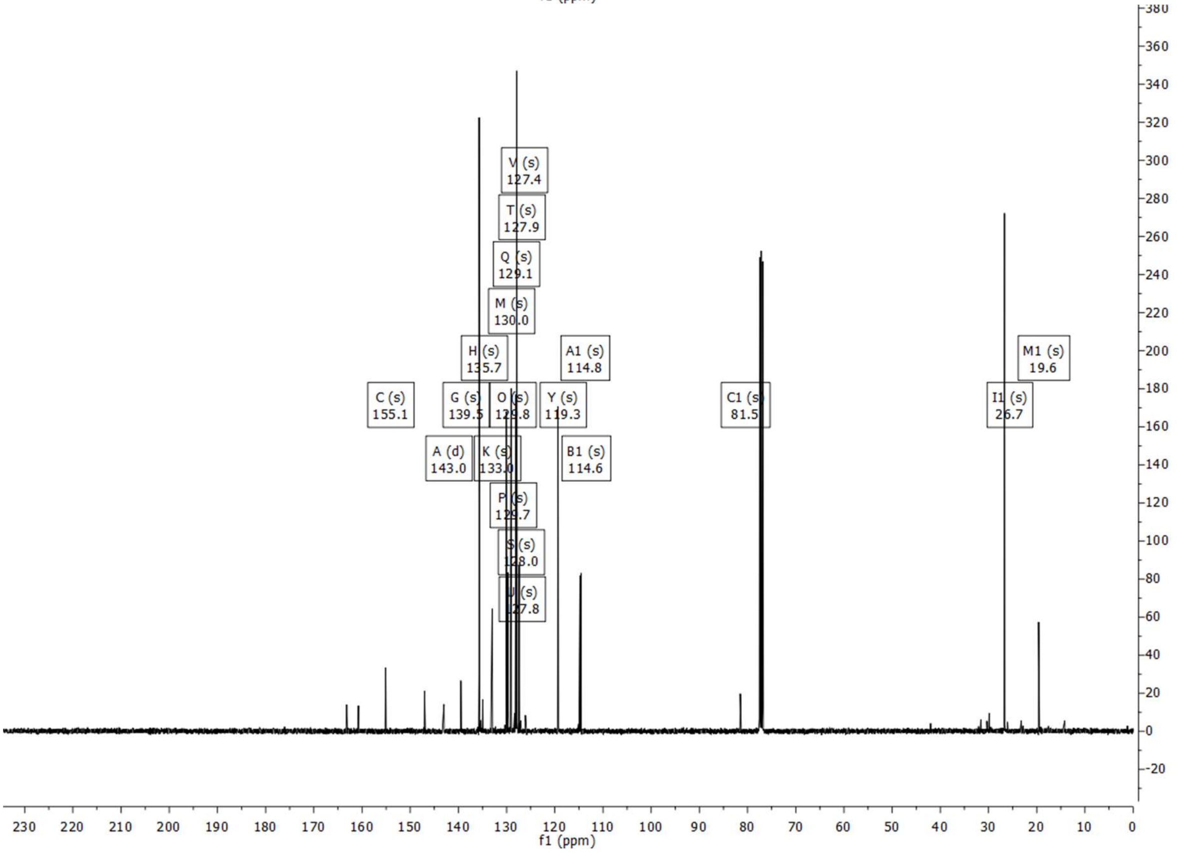

^{1}H and ^{13}C NMR spectra of 2-{4-[(*tert*-Butyldiphenylsilyl)oxy]phenyl}-2-(4-fluorophenyl)-2-phenylacetonitrile **(6)** in $CDCl_3$.

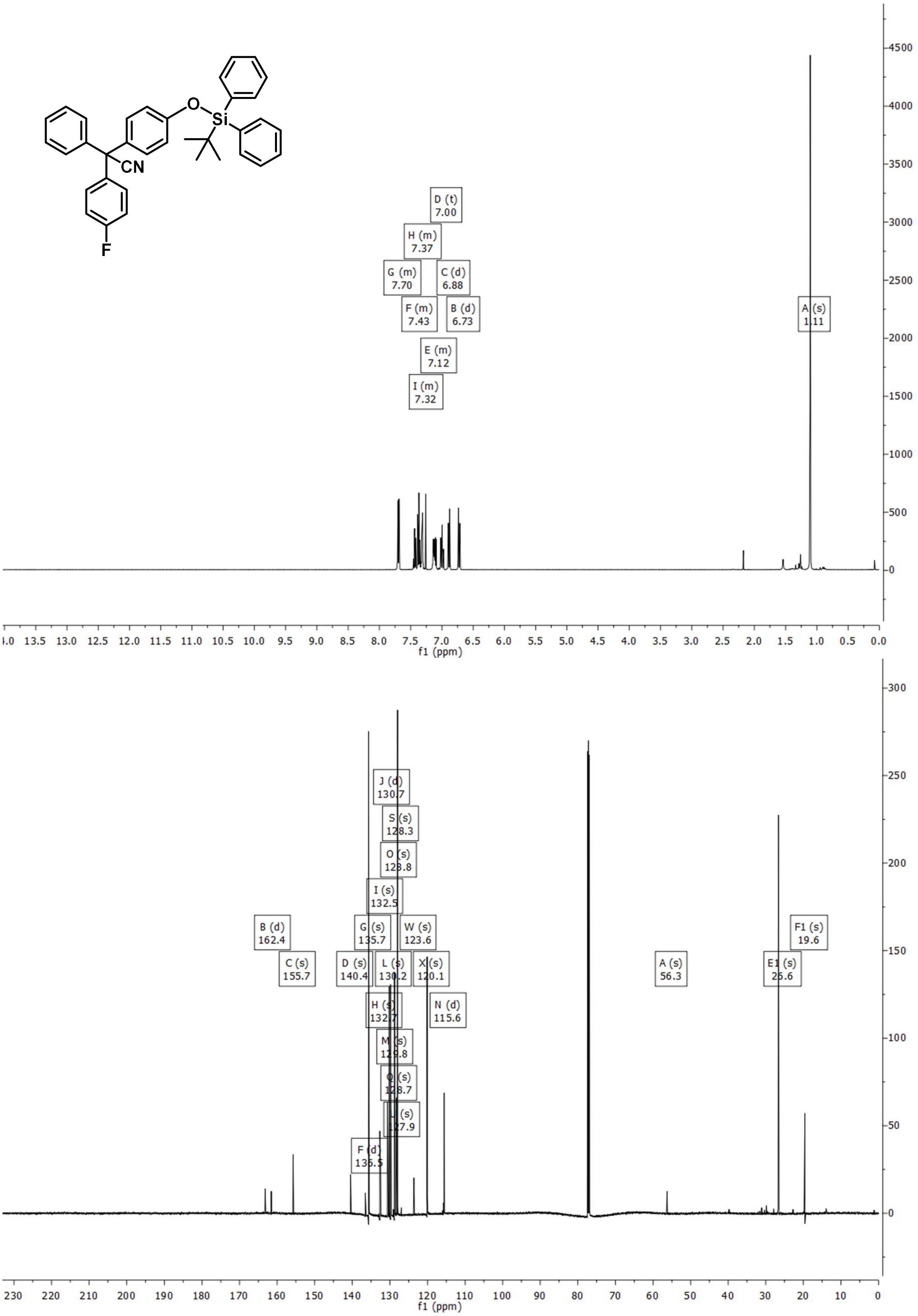

^{1}H and ^{13}C NMR spectra of 2-(4-Fluorophenyl)-2-(4-hydroxyphenyl)-2-phenylacetonitrile **(7)** in $CDCl_3$.

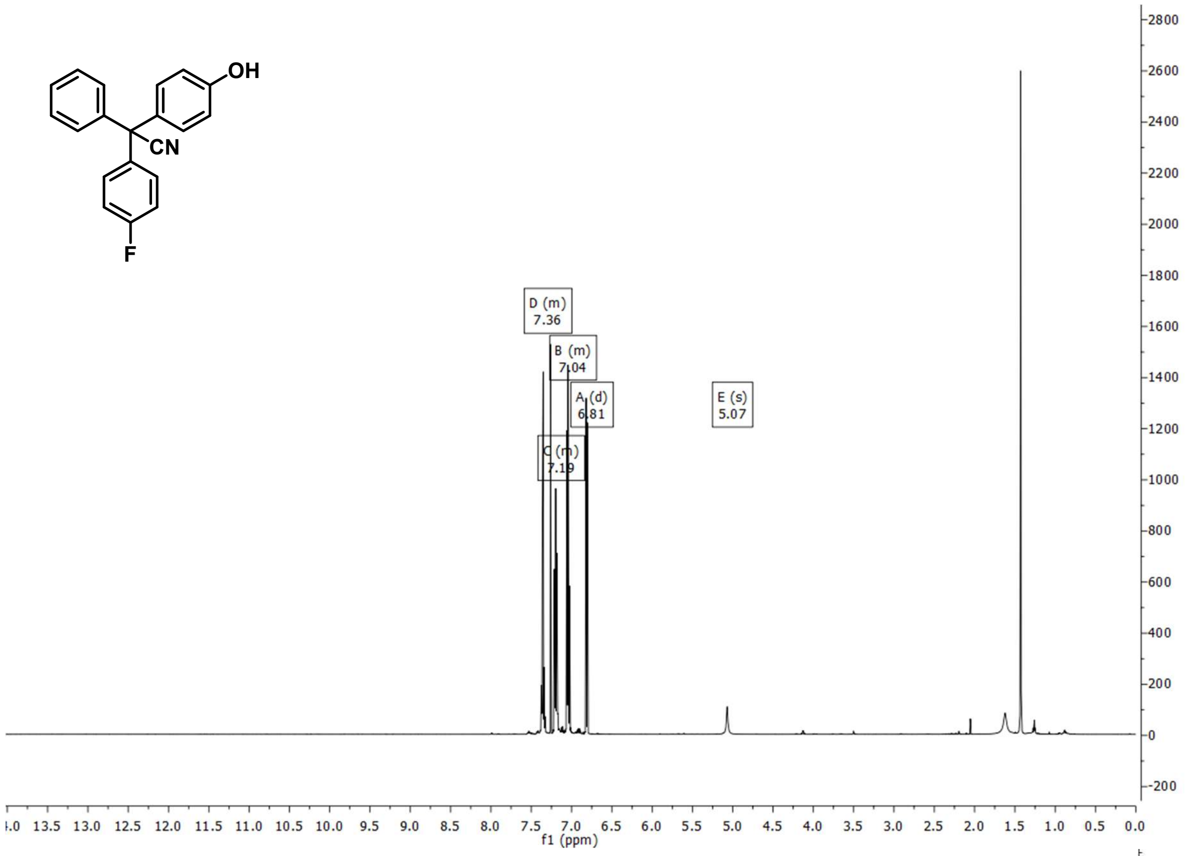

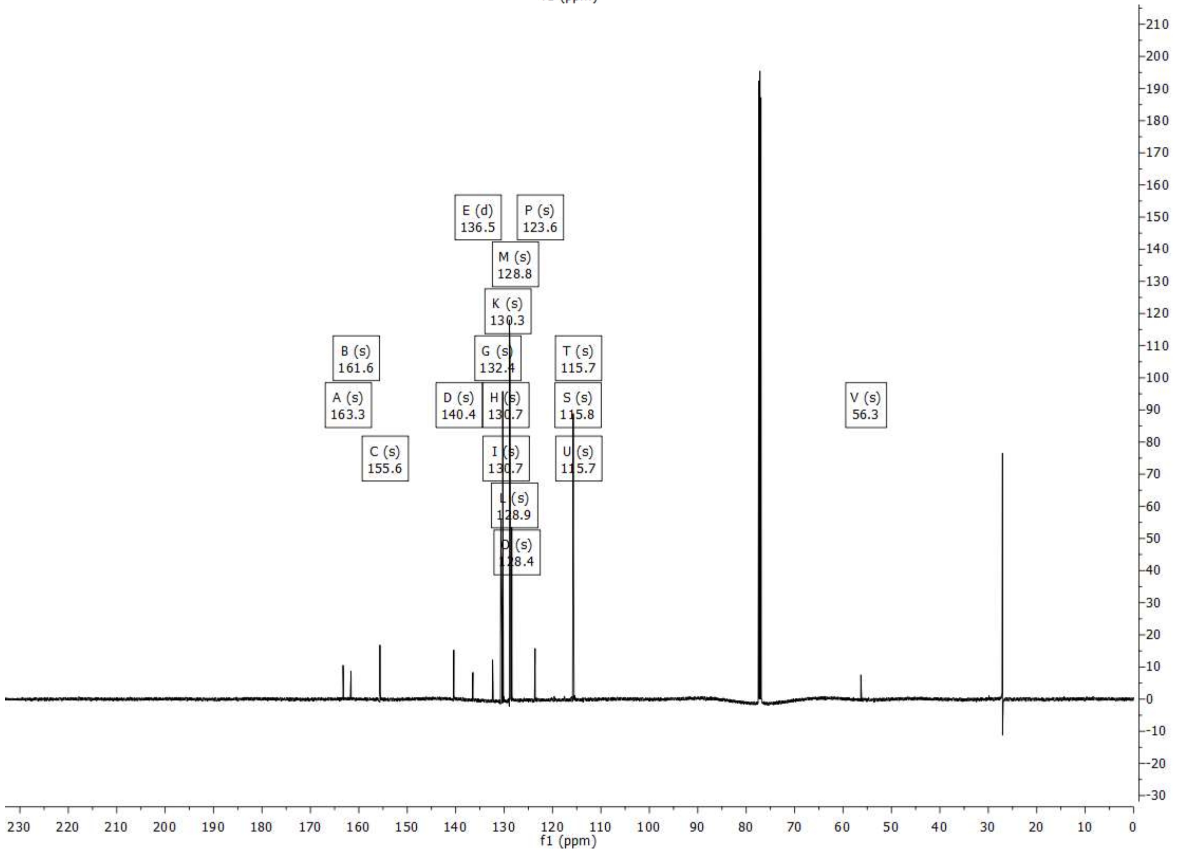

^{1}H and ^{13}C NMR spectra of 2-(4-Fluorophenyl)-2-(4-hydroxyphenyl)-2-phenylacetamide **(2)** in DMSO-d_6.

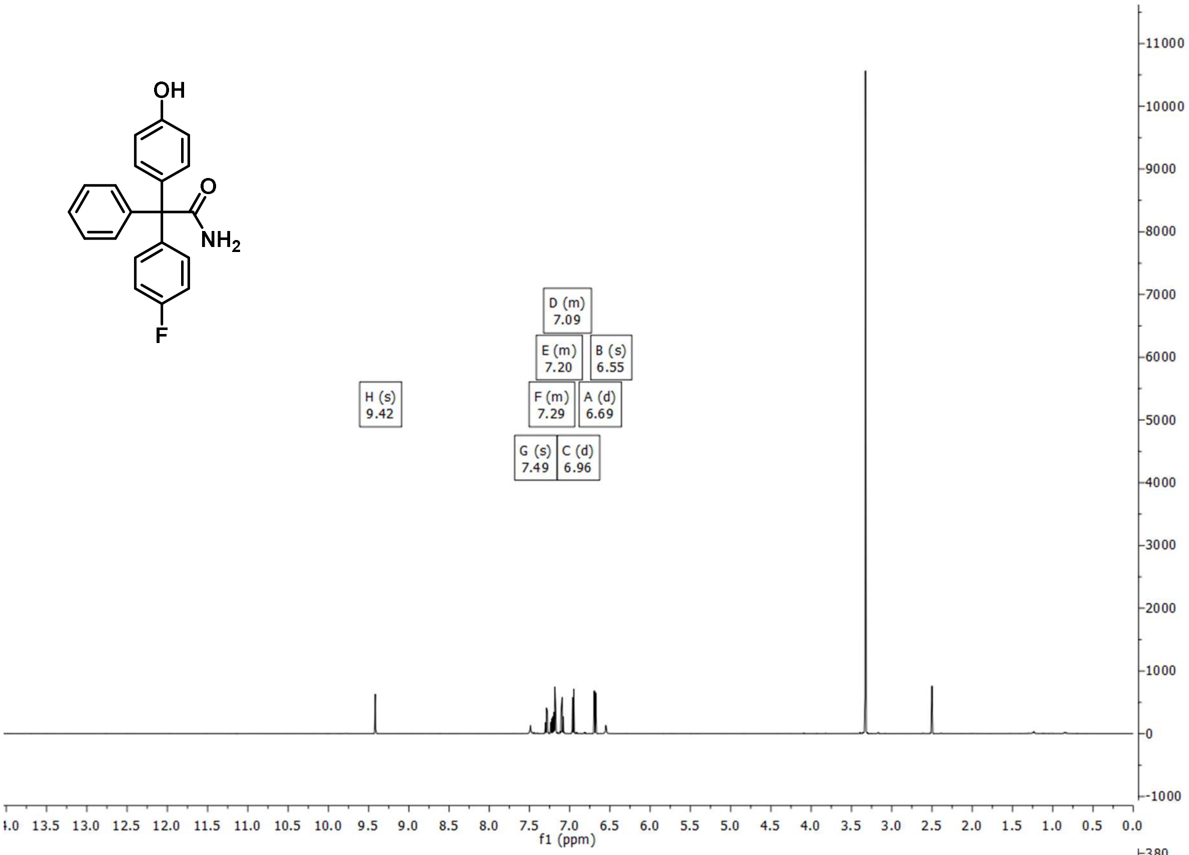

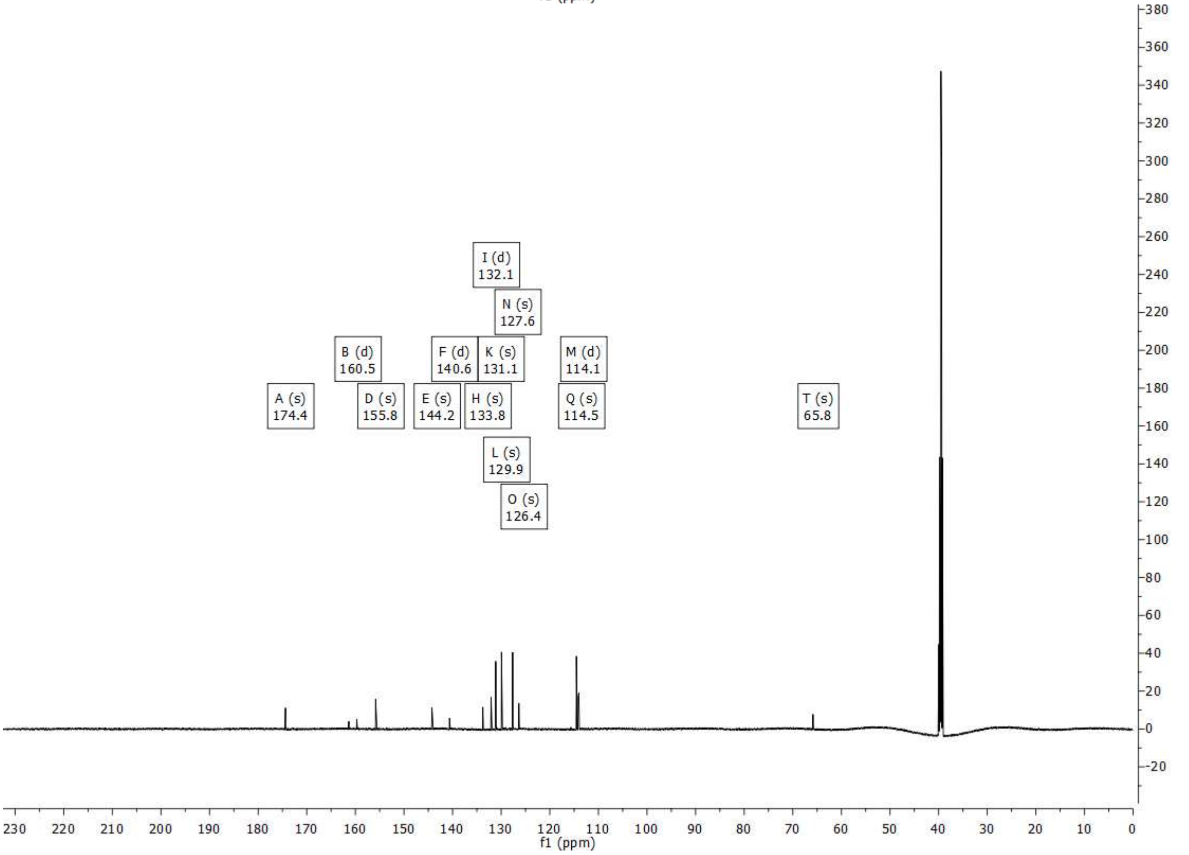

^{1}H and ^{13}C NMR spectra of 2-(4-Fluorophenyl)-2-phenyl-2-[4-(prop-2-yn-1-yloxy)phenyl]acetamide **(8)** in DMSO-d_6.

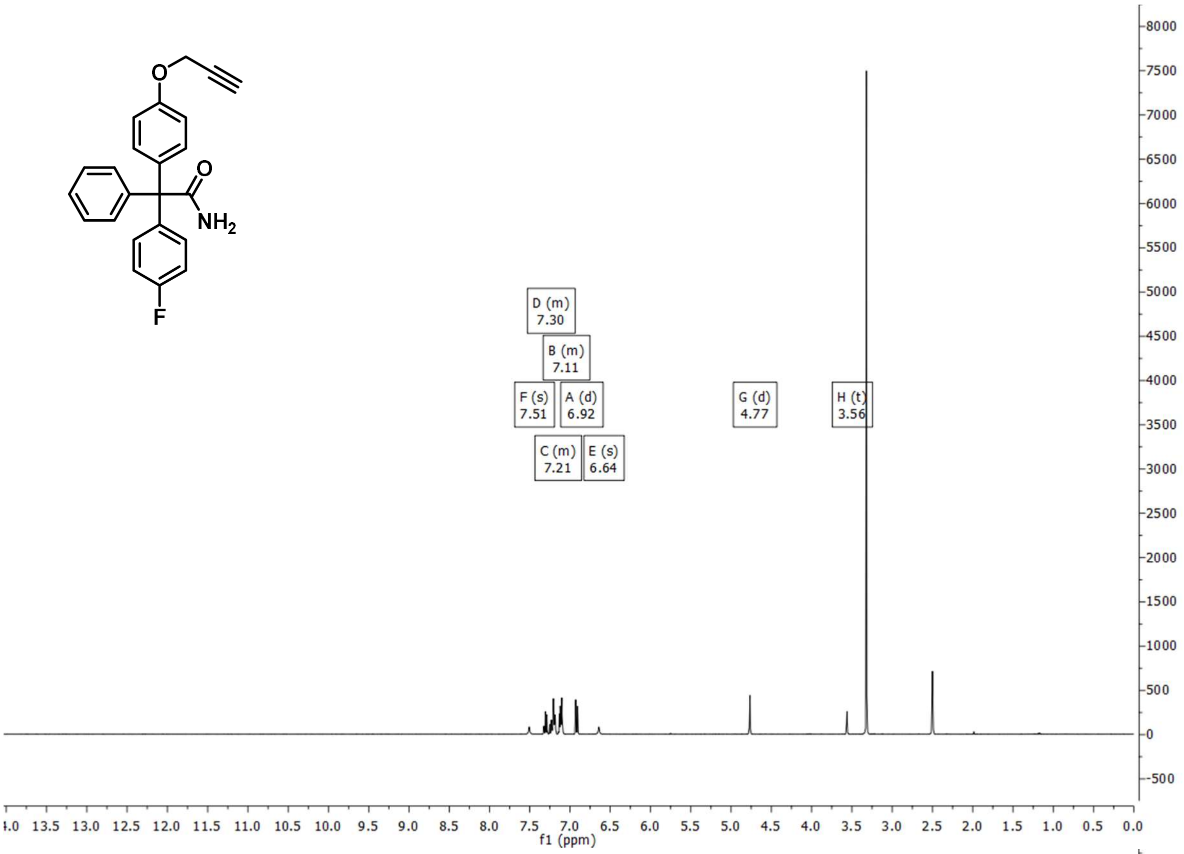

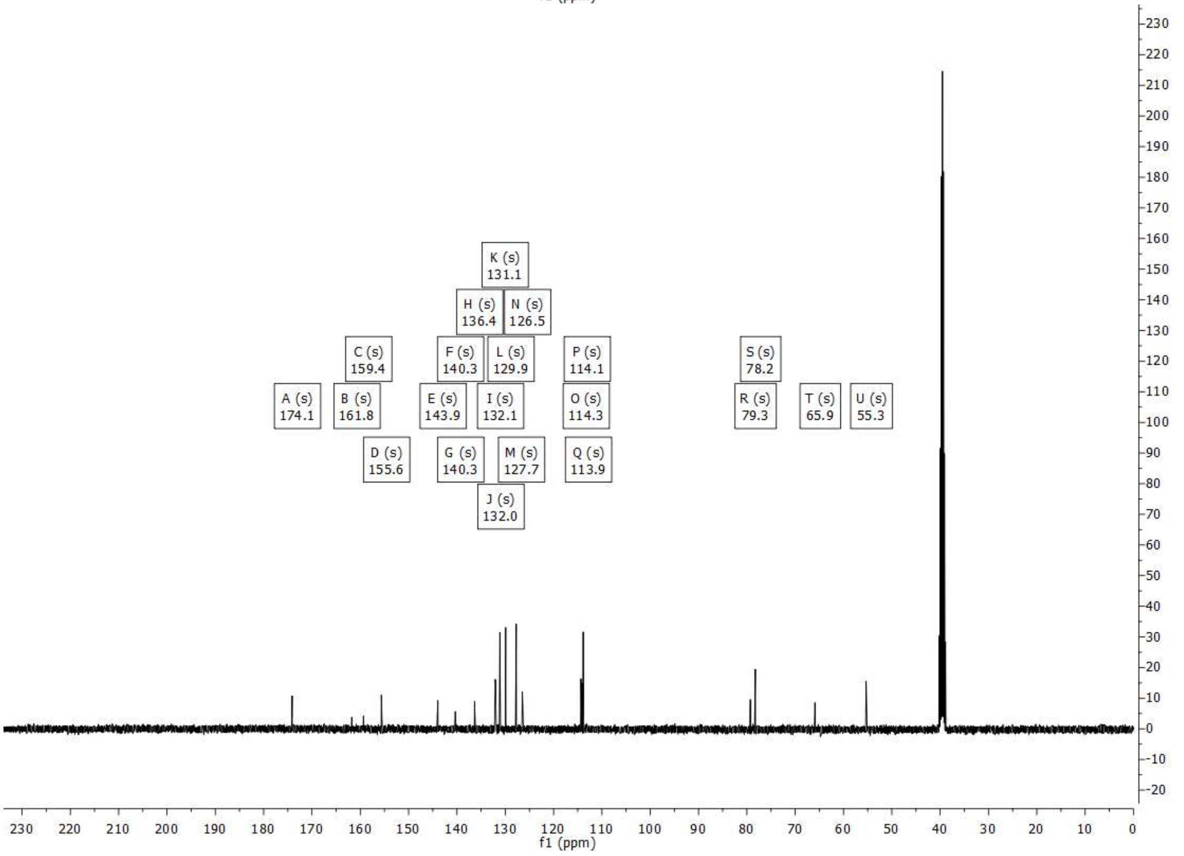

^{1}H and ^{13}C NMR spectra of 10,10-Difluoro-4,6-dimethyldithieno[2,3-b:3',2'-g]-4-bora-3a,4a-diaza-s-indacene **(15)** in $CDCl_3$.

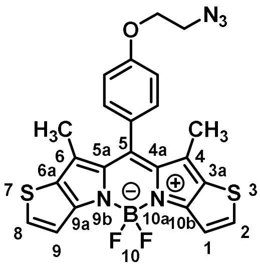

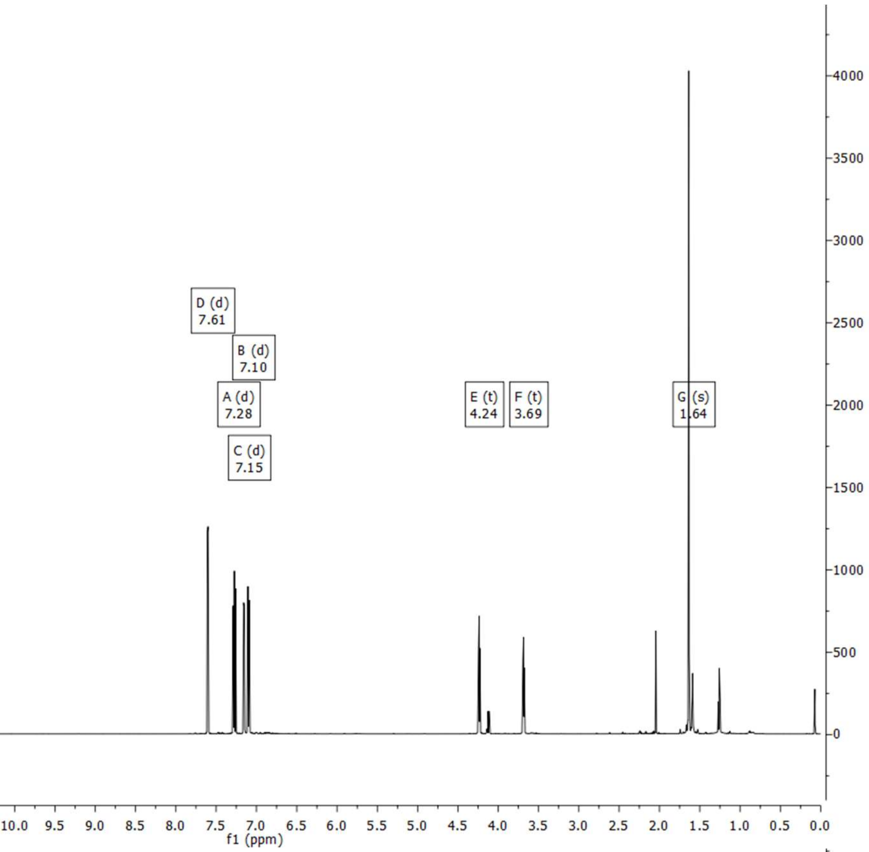

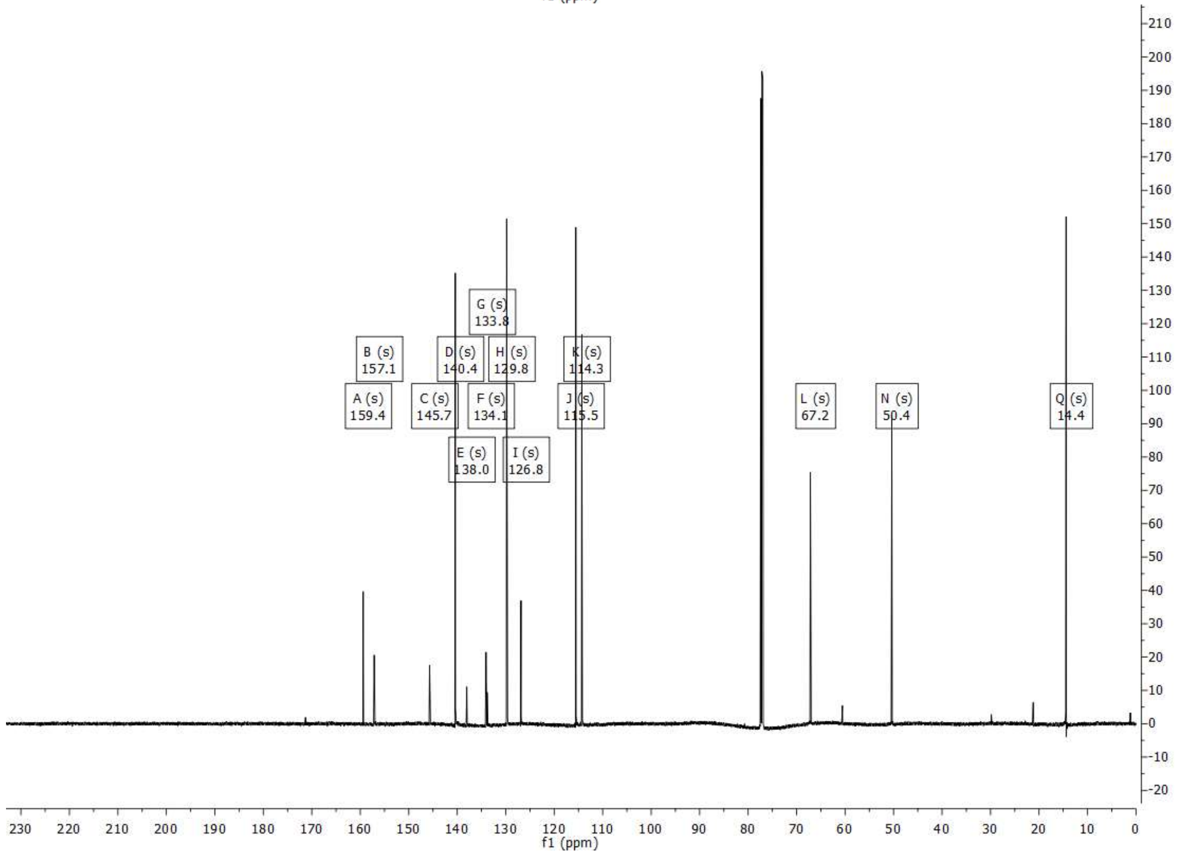

^{1}H and ^{13}C NMR spectra of 8-[4-(2-Azidoethoxy)phenyl]-2,6-diethyl-4,4-difluoro-1,3,5,7-tetramethyl-4-bora-3a,4a-diaza-s-indacene **(16)** in $CDCl_3$.

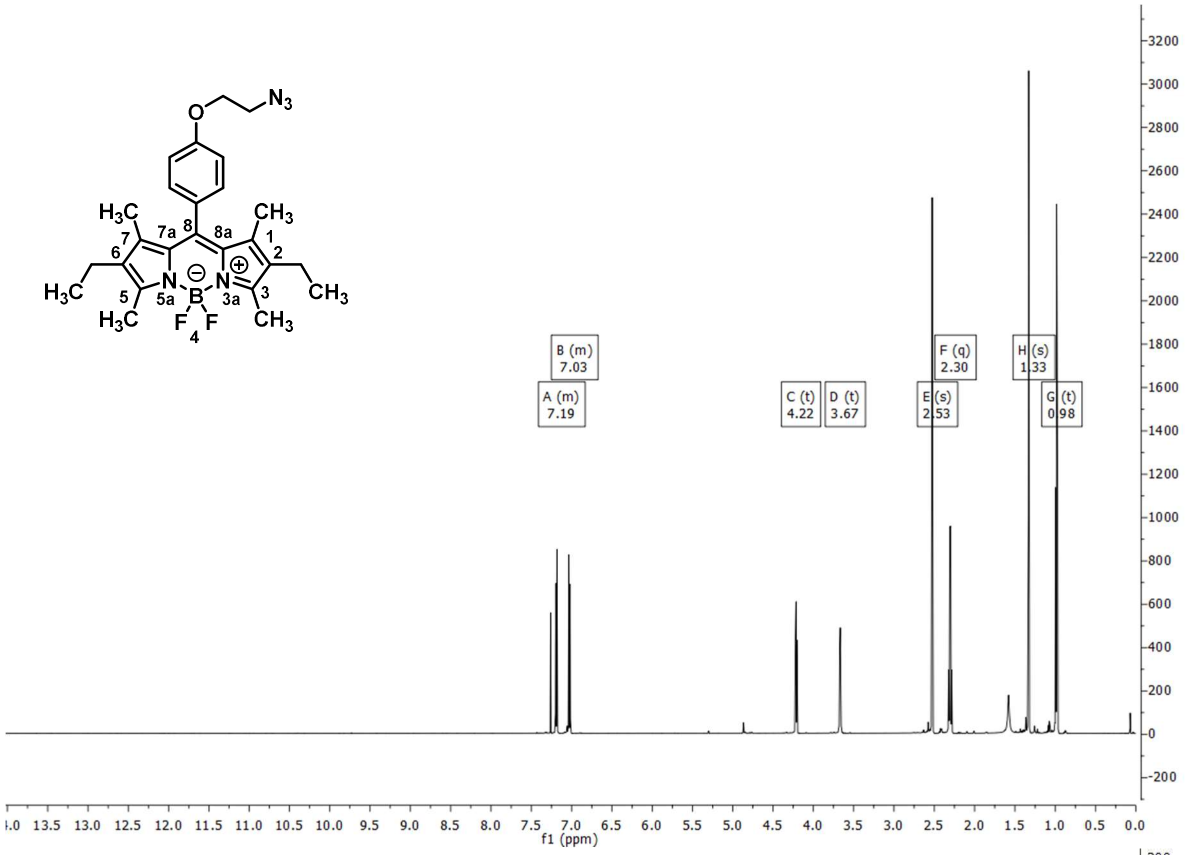

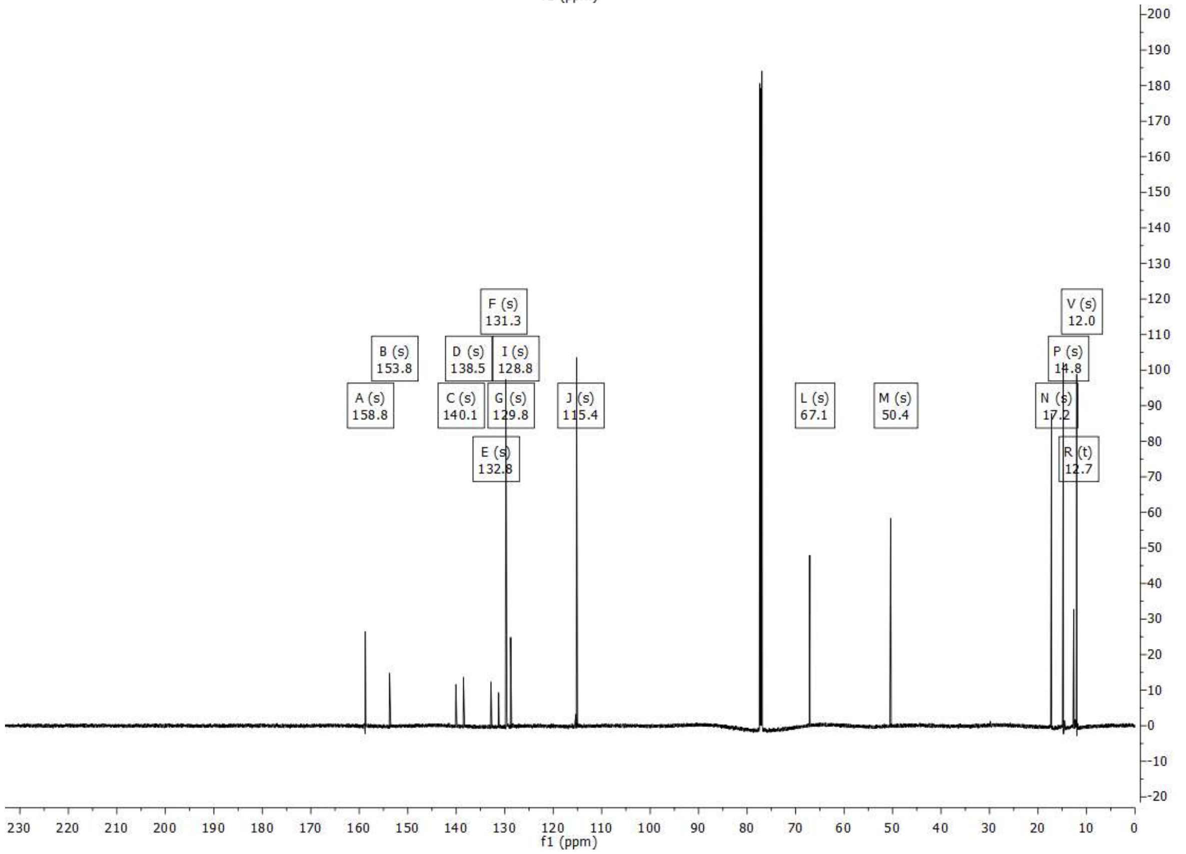

^{1}H and ^{13}C NMR spectra of 8-[4-(12-Azido-1,4,7,10-tetraoxadodecyl)phenyl]-2,6-diethyl-4,4-difluoro-1,3,5,7- tetramethyl-4-bora-3a,4a-diaza-s-indacene **(17)[2]** in $CDCl_3$.

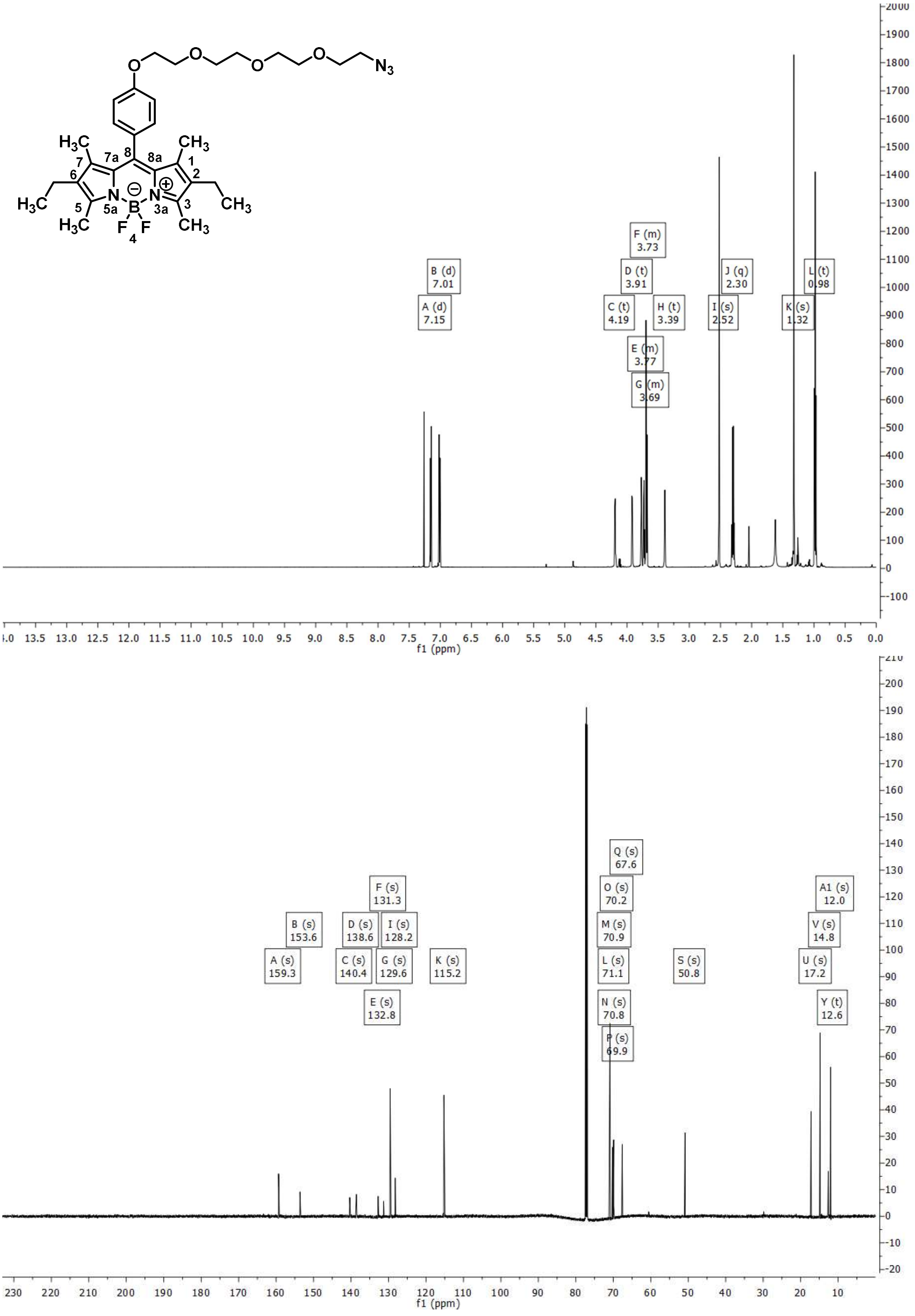

^{1}H and ^{13}C NMR spectra of 8-[4-(12-Azidododecyloxy)phenyl]-2,6-diethyl-4,4-difluoro-1,3,5,7-tetramethyl-4-bora-3a,4a-diaza-s-indacene **(18)[2]** in $CDCl_3$.

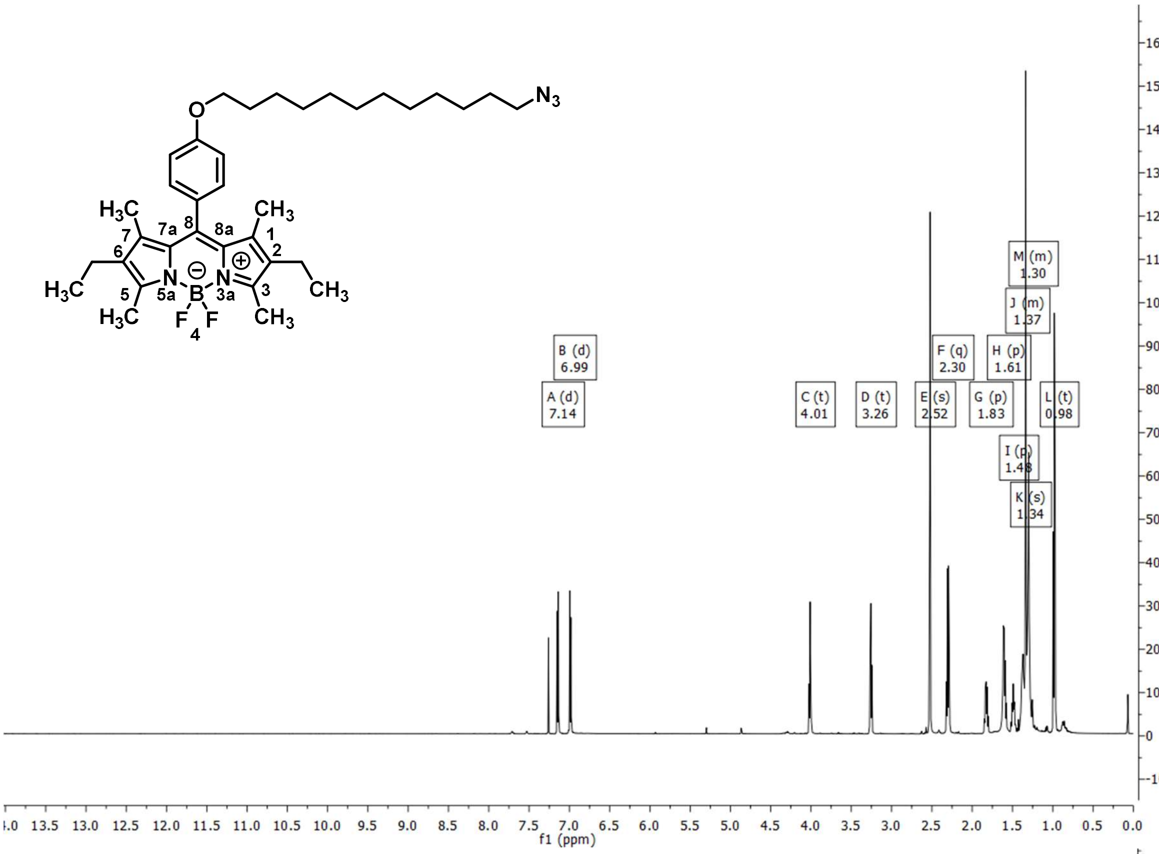

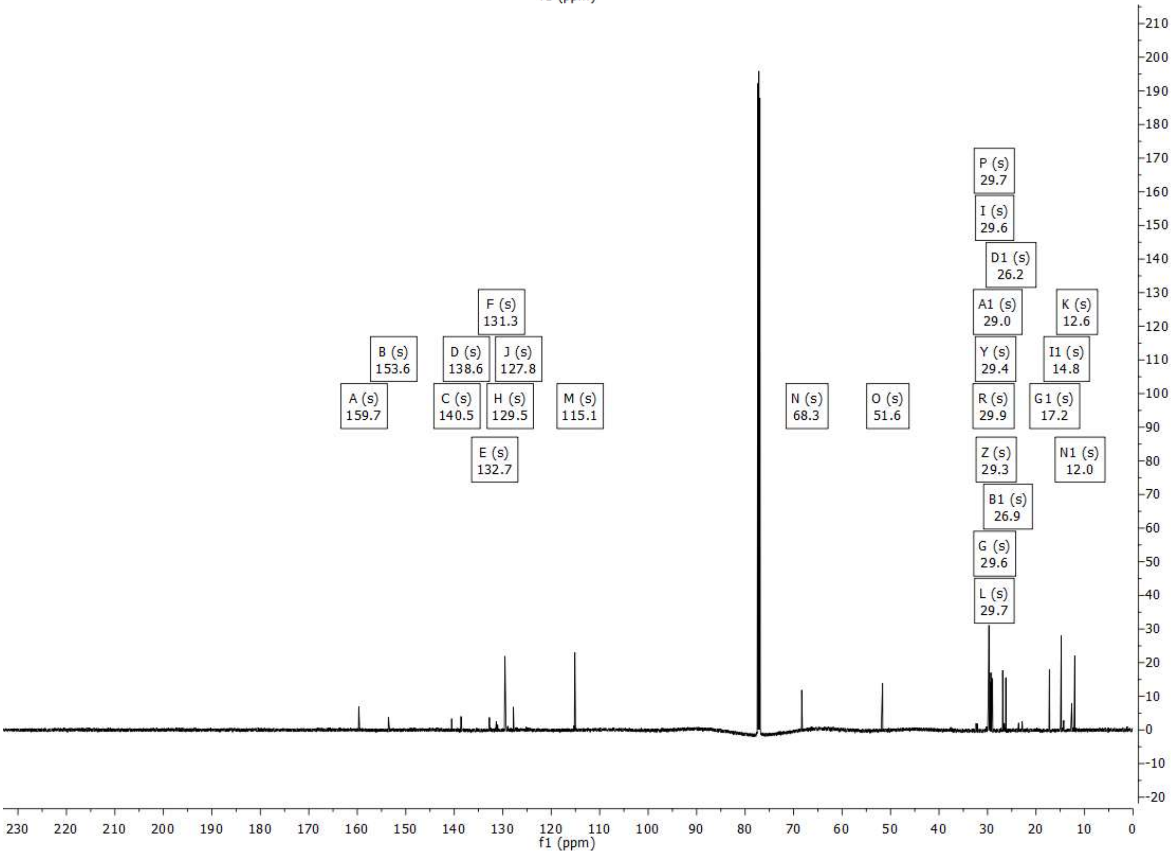

^{1}H and ^{13}C NMR spectra of 8-[4-(2-Azidoethoxy)phenyl]-4,4-difluoro-1,3,5,7-tetramethyl-4-bora-3a,4a-diaza-s-indacene **(19)**[3] in DMSO-d_6.

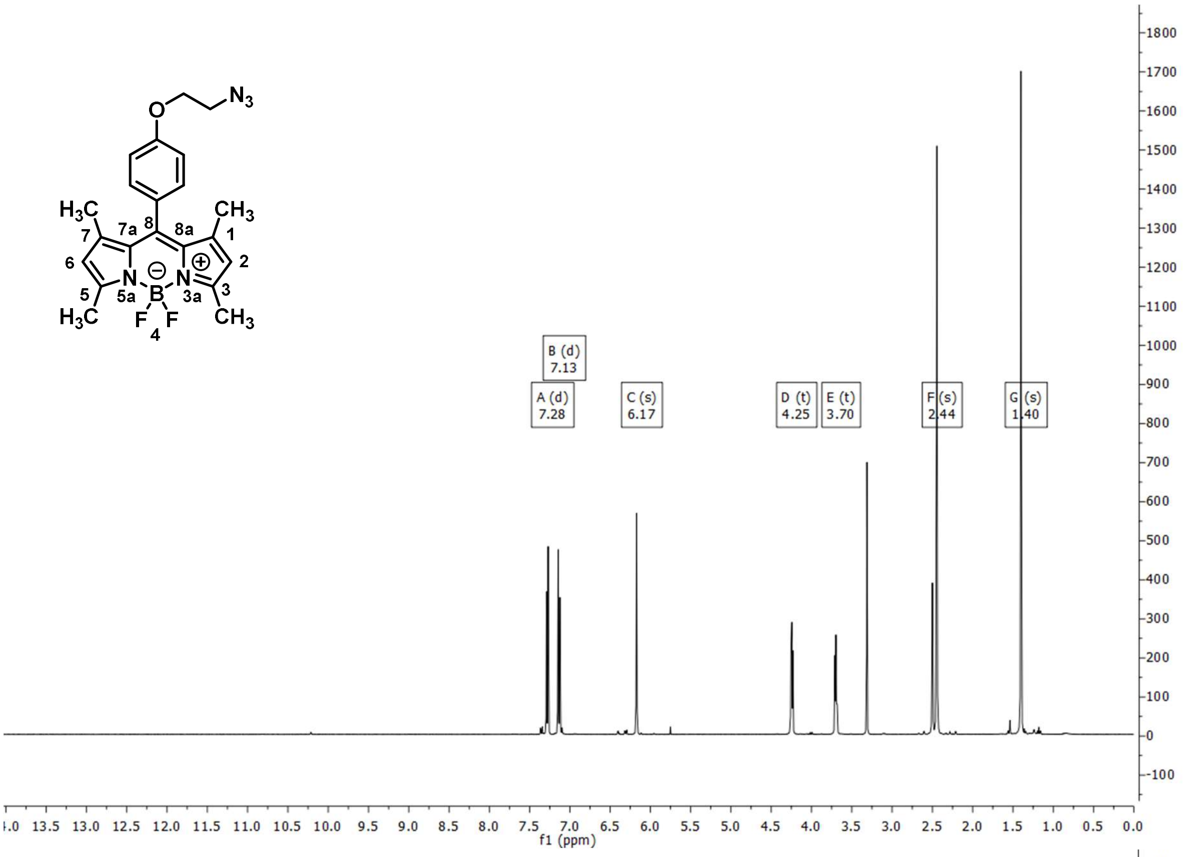

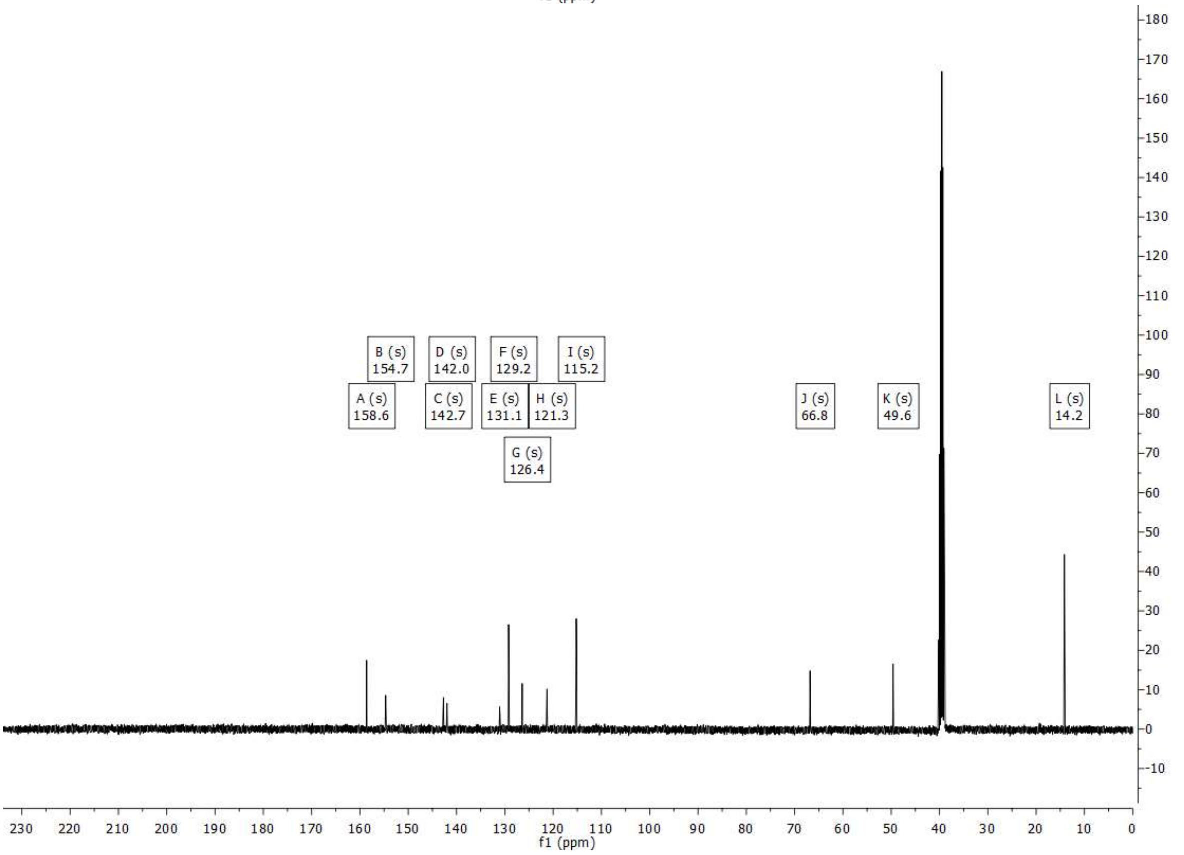

^{1}H and ^{13}C NMR spectra of 8-[4-(12-Azido-1,4,7,10-tetraoxadodecyl)phenyl]-4,4-difluoro-1,3,5,7-tetramethyl-4-bora-3a,4a-diaza-s-indacene **(20)** in $CDCl_3$.

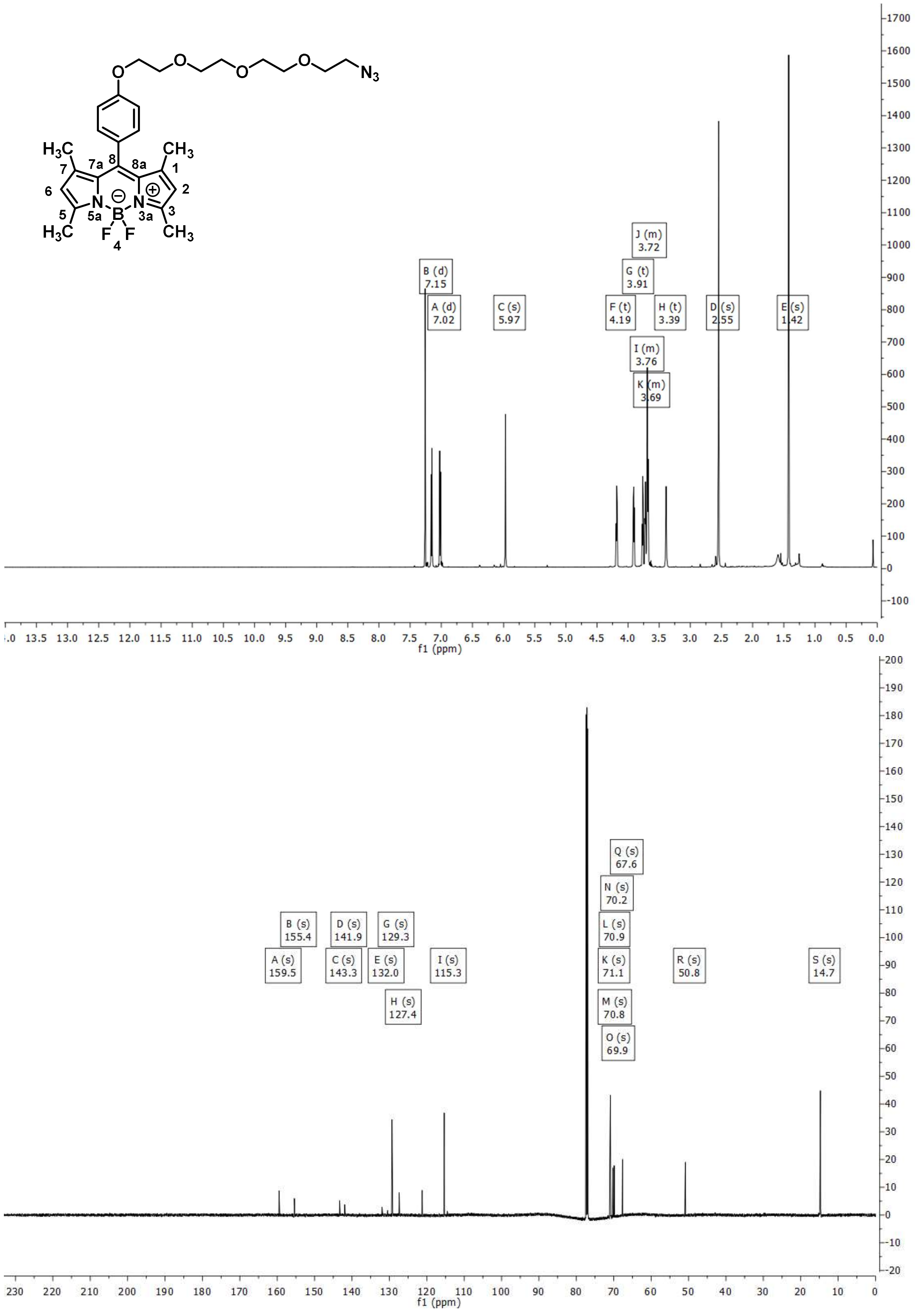

^{1}H and ^{13}C NMR spectra of 2-{4-[(1-{2-[4-(10,10-Difluoro-4,6-dimethyldithieno [2,3-b:3’,2’-g]-4-bora-3a,4a-diaza-s-indacen-8-yl)phenoxy]ethyl}-1,2,3-triazol-4-yl)methoxy]phenyl}-2-(4-flluorophenyl)-2-phenylacetamide **(21)** in $CDCl_3$.

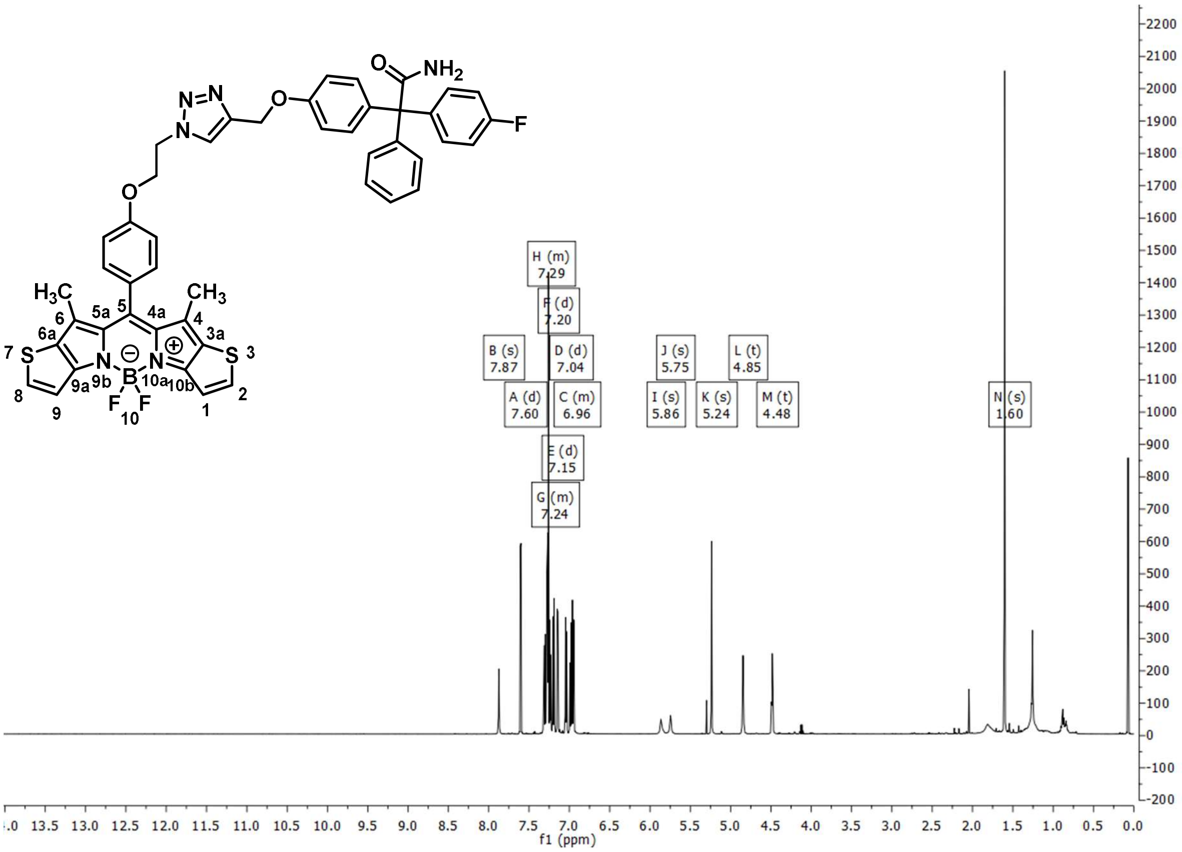

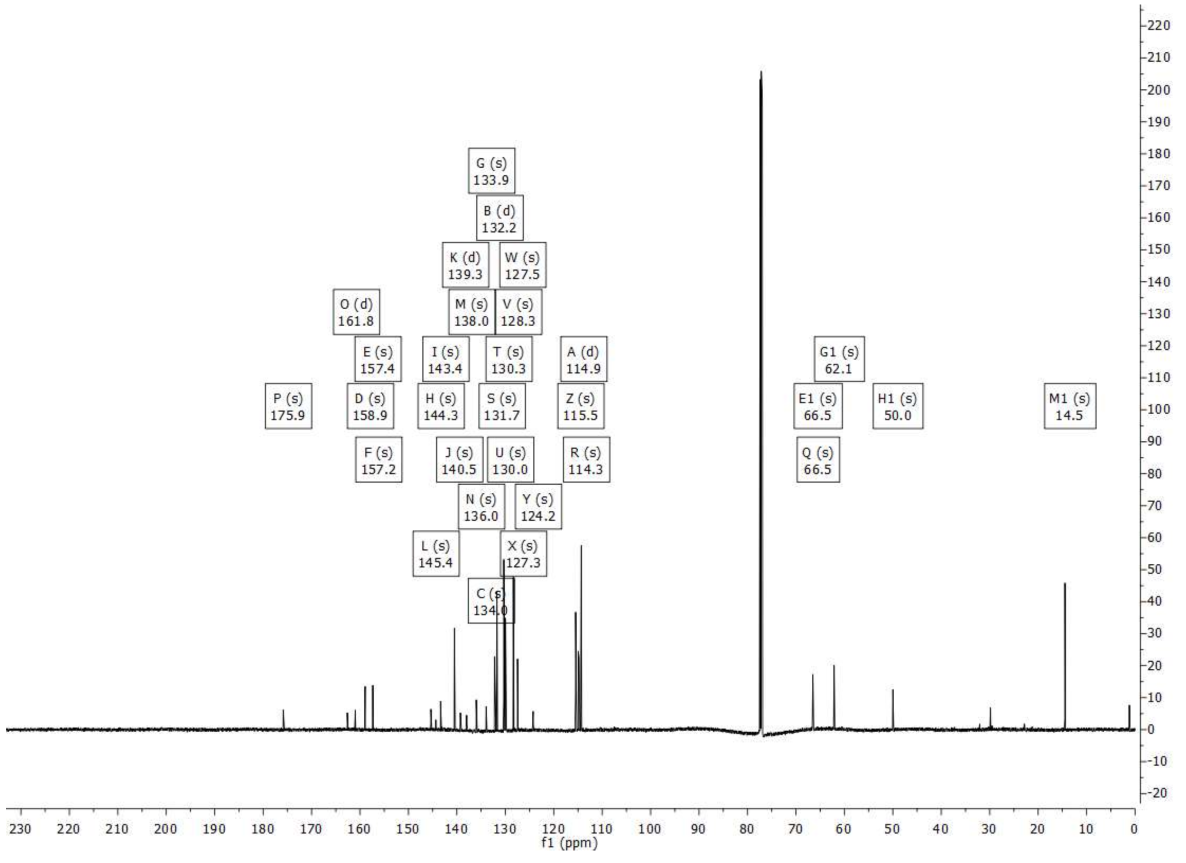

^{1}H and ^{13}C NMR spectra of 2-{4-[(1-{2-[4-(2,6-Diethyl-4,4-difluoro-1,3,5,7-tetramethyl-4-bora-3a,4a-diaza-s-indacen-8-yl)phenoxy]ethyl}-1,2,3-triazol-4-yl)methoxy]phenyl}-2-(4-flluorophenyl)-2-phenylacetamide **(22)** in CD_2Cl_2.

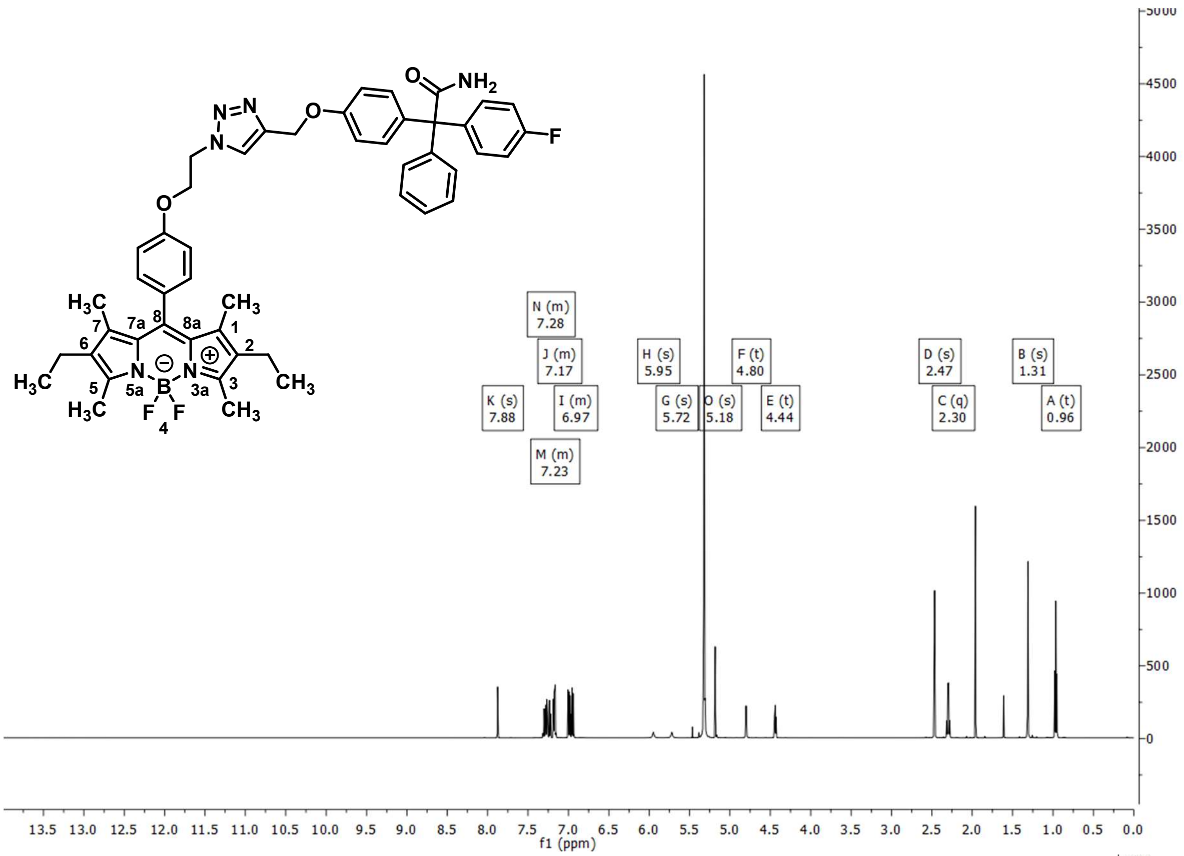

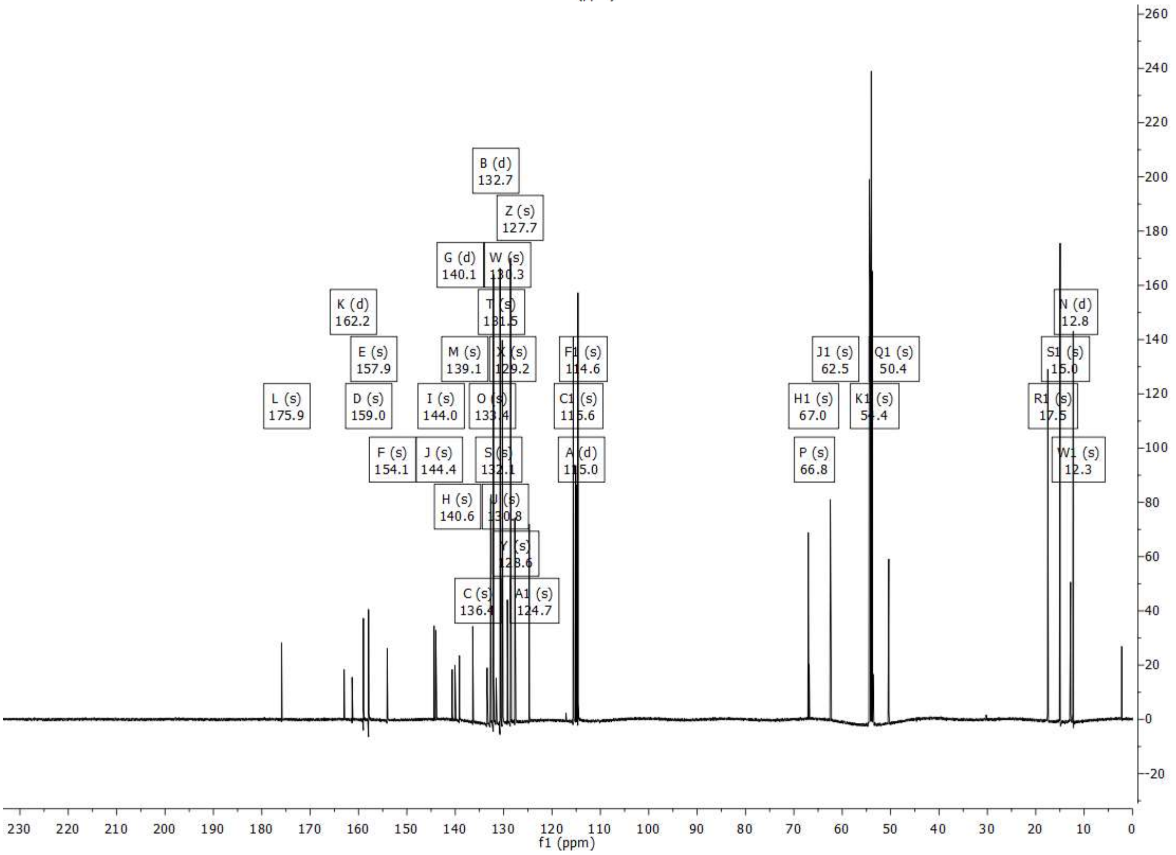

^{1}H and ^{13}C NMR spectra of 2-{4-[(1-{11-[4-(2,6-Diethyl-4,4-difluoro-1,3,5,7-tetramethyl-4-bora-3a,4a-diaza-s-indacen-8-yl)phenoxy]-3,6,9-trioxaundecyl}-1,2,3-triazol-4-yl)methoxy]phenyl}-2-(4-fluorophenyl)-2-phenylacetamide **(23)** in CD_2Cl_2.

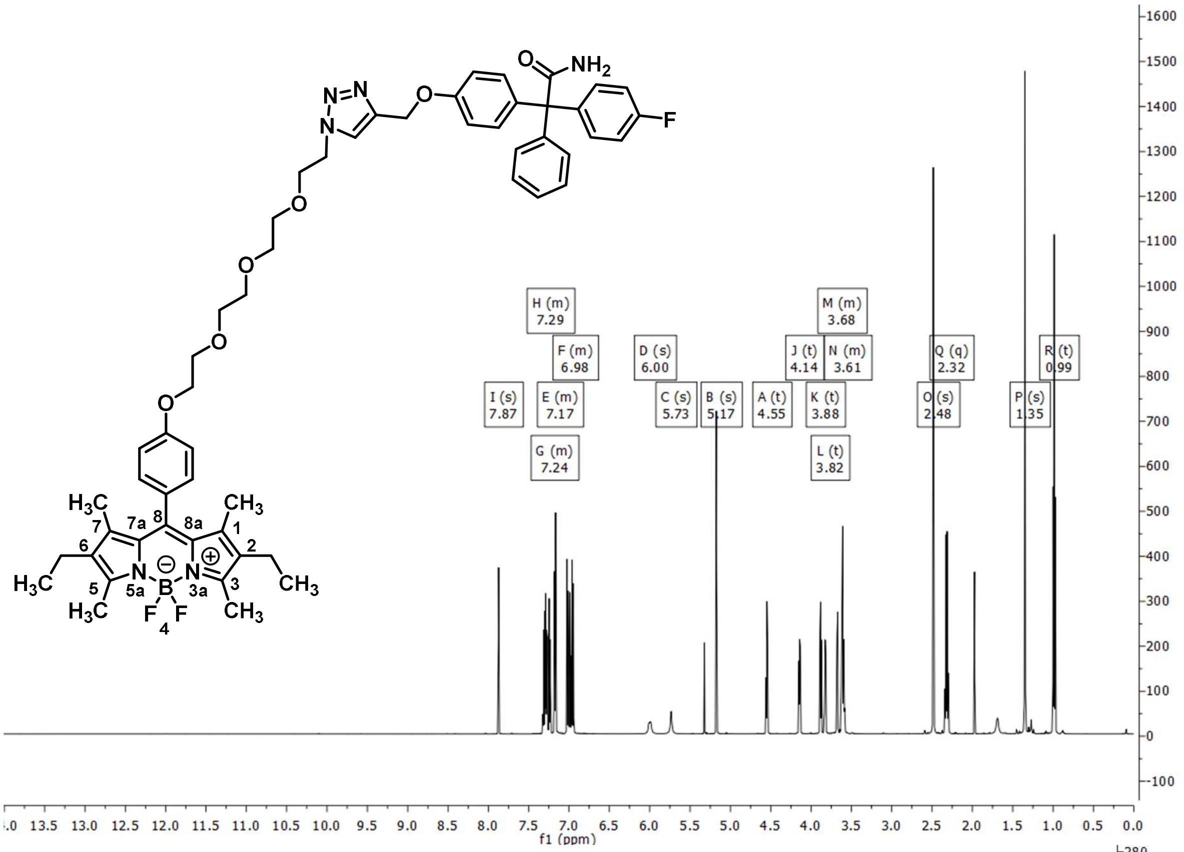

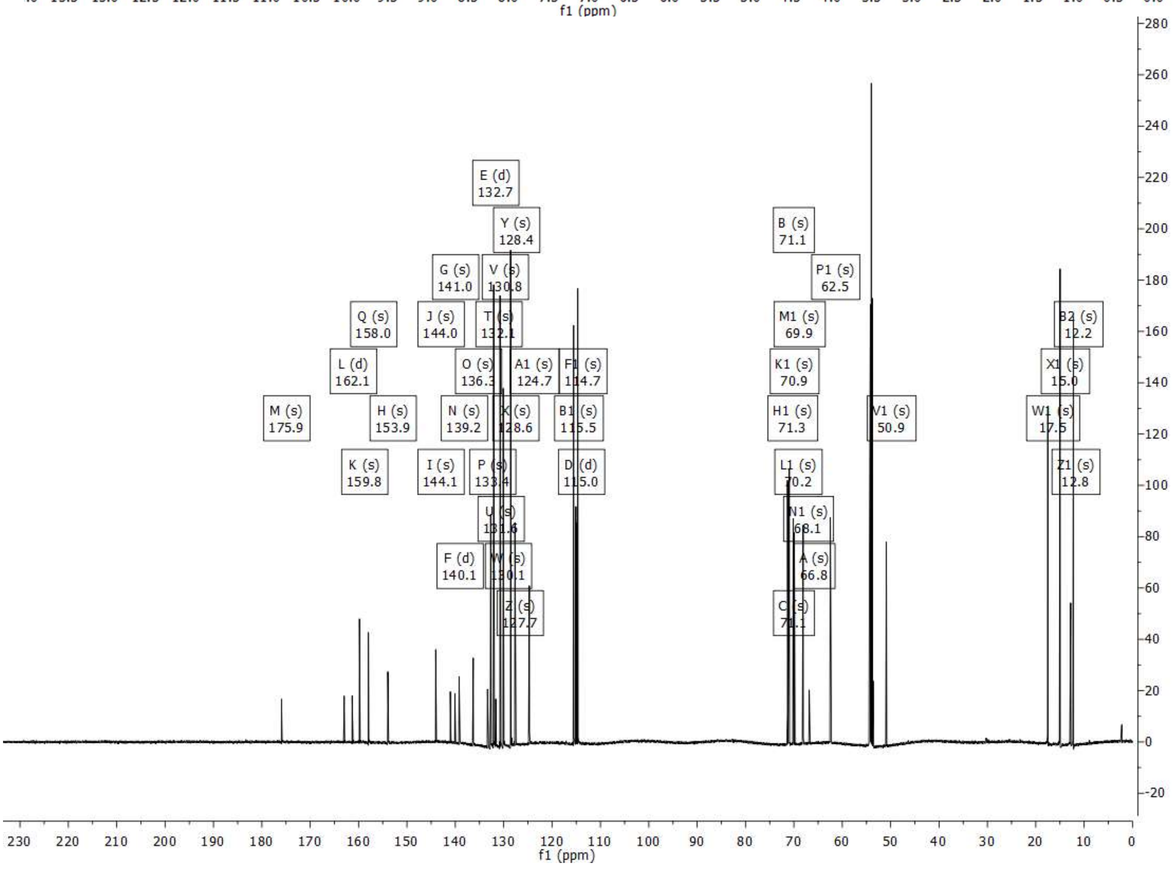

^{1}H and ^{13}C NMR spectra of 2-{4-[(1-{12-[4-(2,6-Diethyl-4,4-difluoro-1,3,5,7-tetramethyl-4-bora-3a,4a-diaza-s-indacen-8-yl)phenoxy]dodecyl}-1,2,3-triazol-4-yl)methoxy]phenyl}-2-(4-fluorophenyl)-2-phenylacetamide **(24)** in $CDCl_3$.

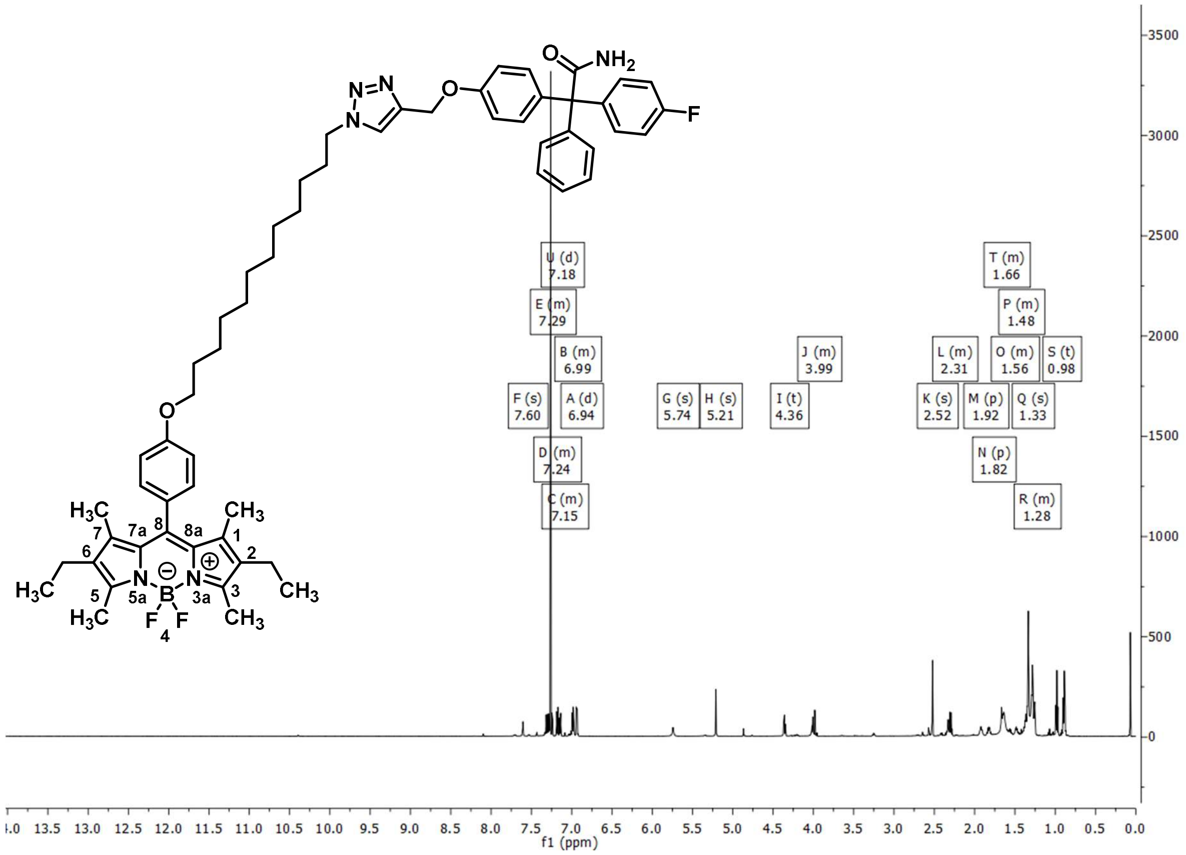

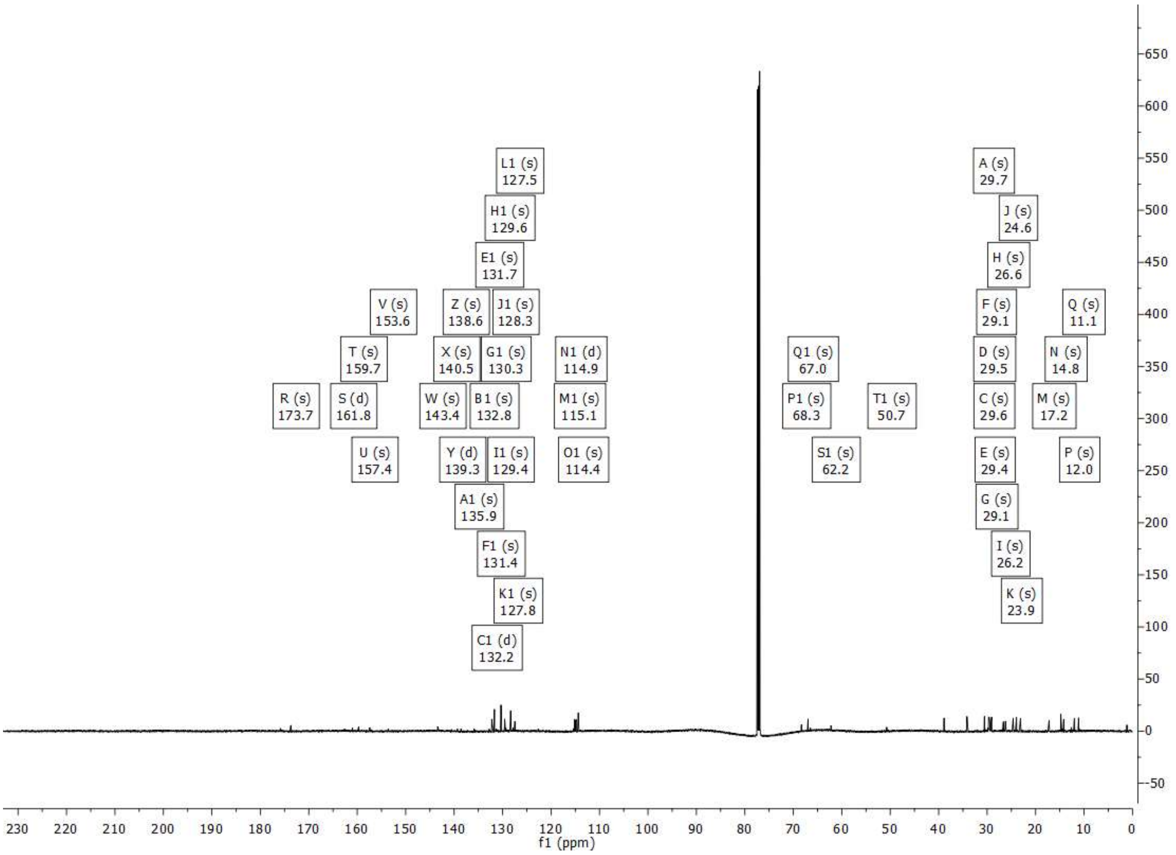

^{1}H and ^{13}C NMR spectra of 2-{4-[(1-{2-[4-(4,4-Difluoro-1,3,5,7-tetramethyl-4-bora-3a,4a-diaza-s-indacen-8-yl)phenoxy]ethyl}-1,2,3-triazol-4-yl)methoxy]phenyl}-2-(4-flluorophenyl)-2-phenylacetamide **(25)** in $CDCl_3$.

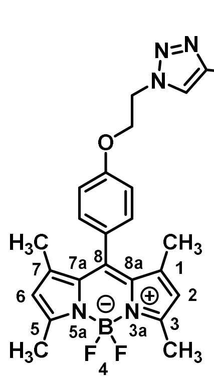

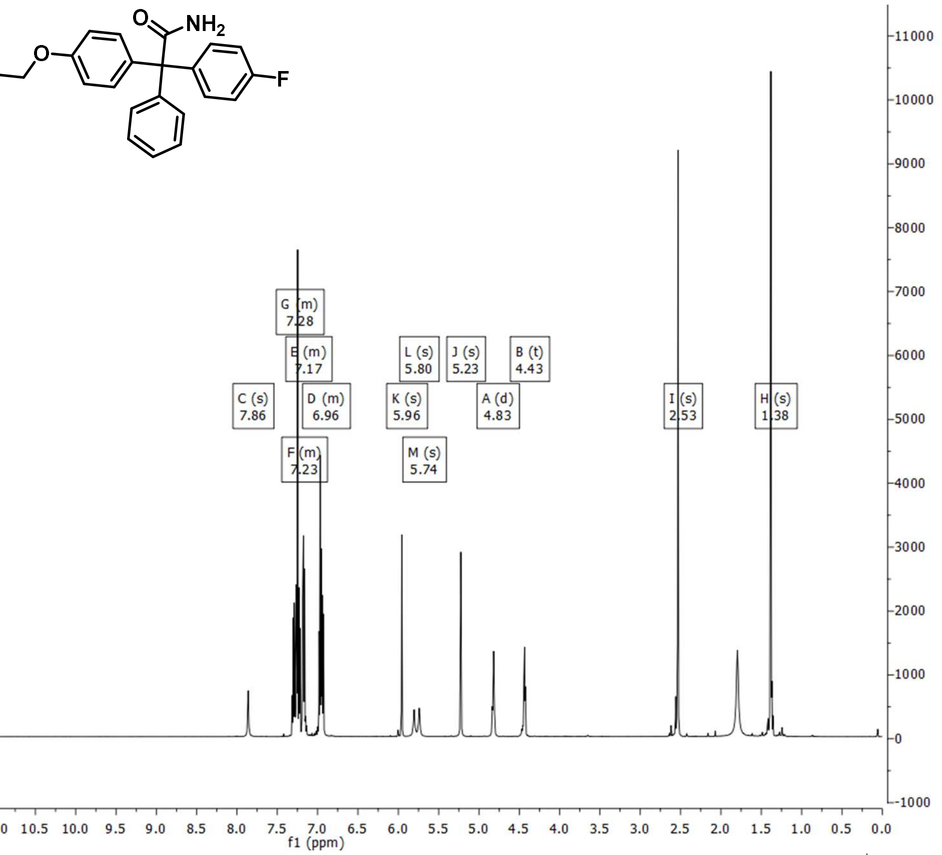

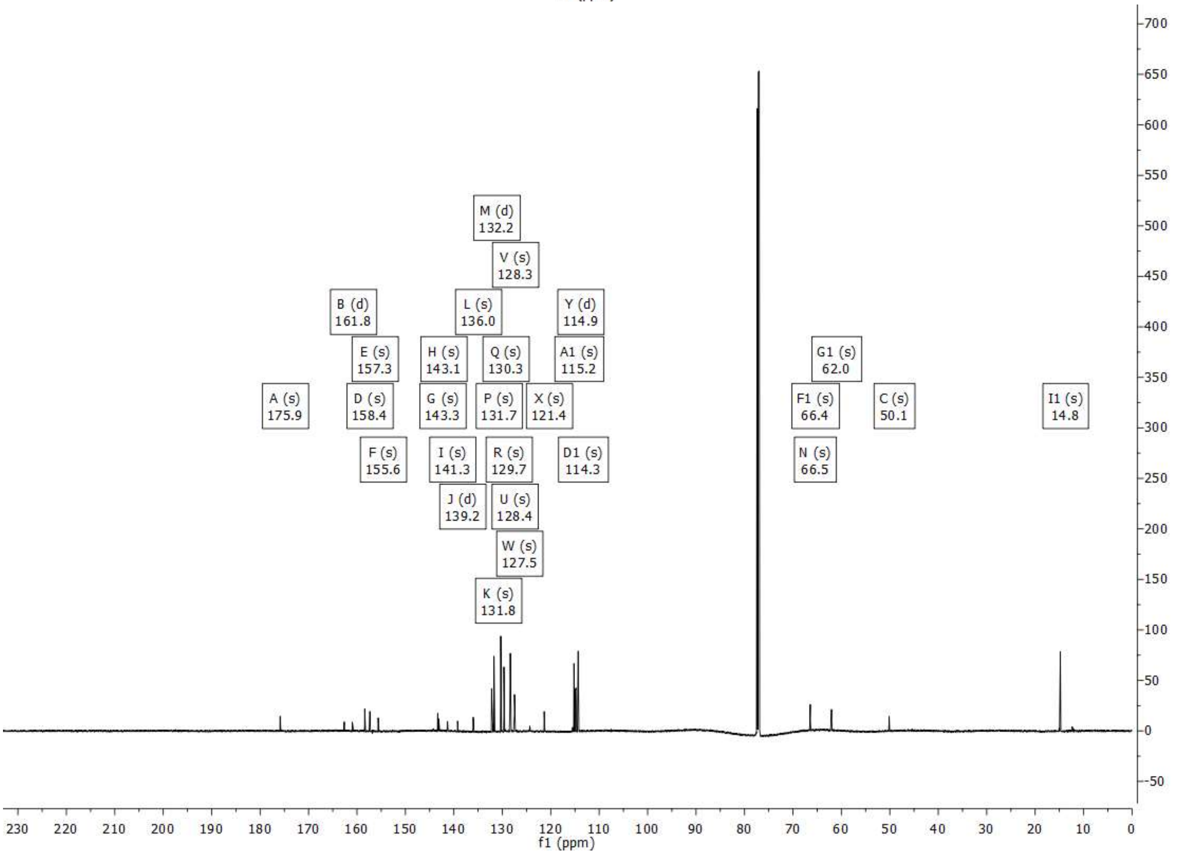

^{1}H and ^{13}C NMR spectra of 2-{4-[(1-{11-[4-(4,4-Difluoro-1,3,5,7-tetramethyl-4-bora-3a,4a-diaza-s-indacen-8-yl)phenoxy]3,6,9-trioxaundecyl}-1,2,3-triazol-4-yl)methoxy]phenyl}-2-(4-flluorophenyl)-2-phenylacetamide **(26)** in $CDCl_3$.

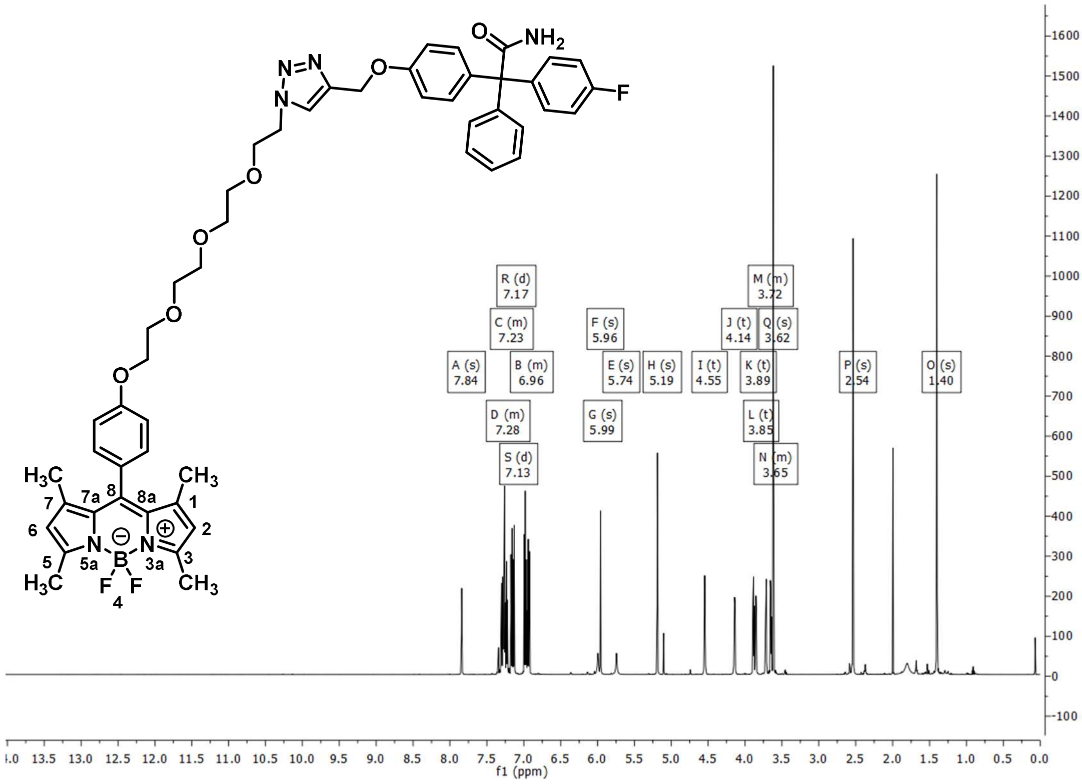

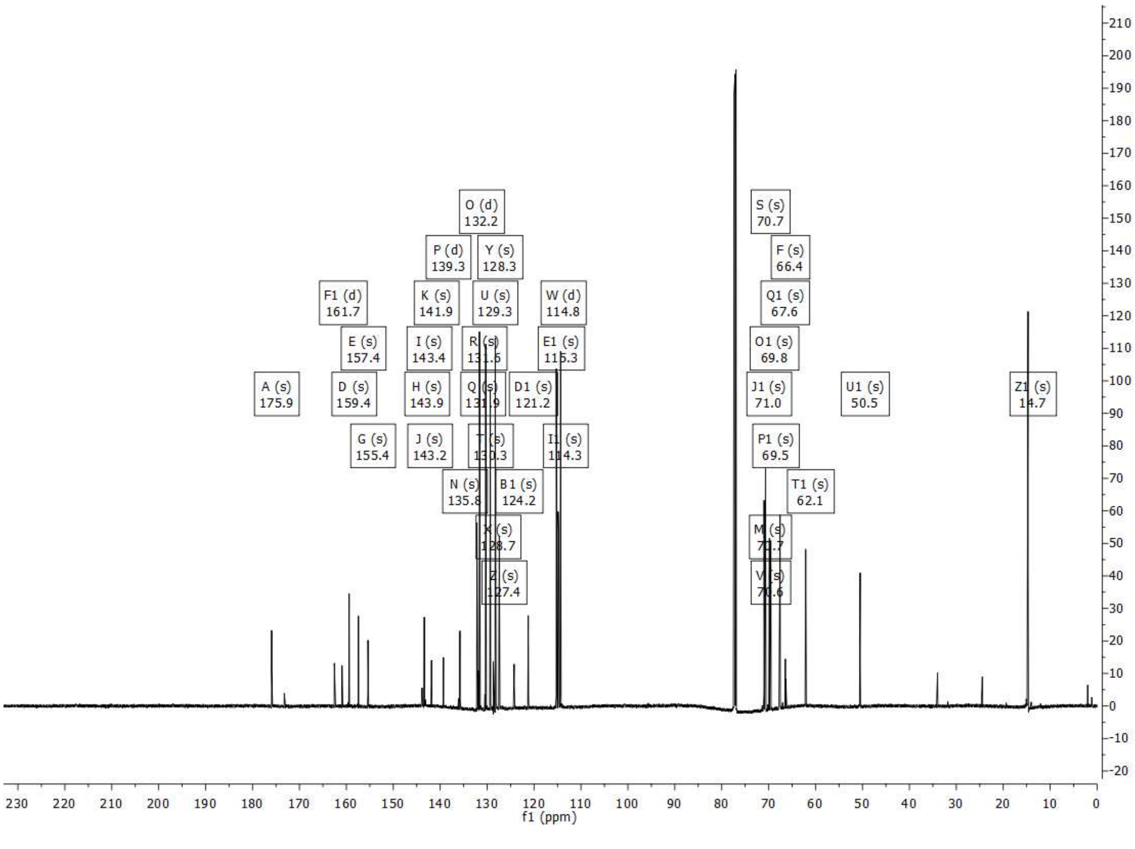

7. HPLC traces of key target compounds

HPLC trace of **15**

Chromatogram and Results

Injection Details

Injection Name:	**KB82a**	*Run Time (min):*	**40,00**
Vial Number:	**GB5**	*Injection Volume:*	**5,00**
Injection Type:	**Unknown**	*Channel:*	**UV_VIS_1**
Calibration Level:		*Wavelength:*	**210,0**
Instrument Method:	**Chromni - steiler**	*Bandwidth:*	**n.a.**
Processing Method:	**Chromni Processing Method - steiler**	*Dilution Factor:*	**1,0000**
Injection Date/Time:	**28.Jun.18 00:52**	*Sample Weight:*	**1,0000**

Chromatogram

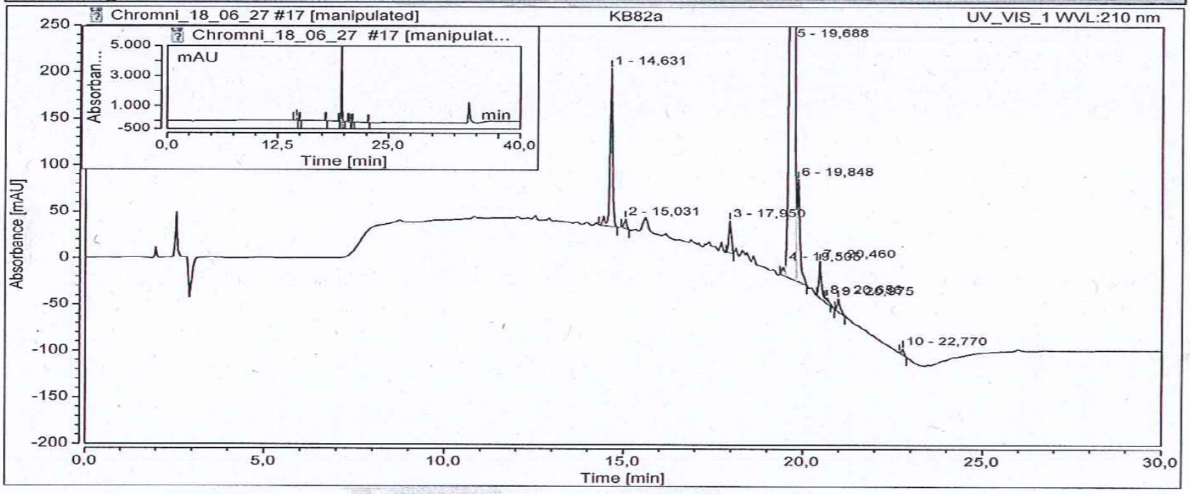

Integration Results

No.	Peak Name	Retention Time min	Area mAU*min	Height mAU	Relative Area %	Relative Height %	Amount n.a.
1		14,631	13,853	169,576	3,11	3,13	n.a.
2		15,031	0,808	10,124	0,18	0,19	n.a.
3		17,950	2,798	34,130	0,63	0,63	n.a.
4		19,505	0,980	13,017	0,22	0,24	n.a.
5		19,688	410,874	5021,604	92,13	92,63	n.a.
6		19,848	10,830	109,718	2,43	2,02	n.a.
7		20,460	3,883	39,264	0,87	0,72	n.a.
8		20,656	0,202	3,093	0,05	0,06	n.a.
9		20,975	1,330	14,113	0,30	0,26	n.a.
10		22,770	0,431	6,409	0,10	0,12	n.a.
Total:			445,989	5421,048	100,00	100,00	

HPLC trace of **16**

Chromatogram and Results

Injection Details

Injection Name:	**KB51b**	*Run Time (min):*	**30,00**
Vial Number:	**GA8**	*Injection Volume:*	**5,00**
Injection Type:	**Unknown**	*Channel:*	**UV_VIS_1**
Calibration Level:		*Wavelength:*	**210,0**
Instrument Method:	**Chromni**	*Bandwidth:*	**n.a.**
Processing Method:	**Chromni Processing Method**	*Dilution Factor:*	**1,0000**
Injection Date/Time:	**18.Okt.17 20:57**	*Sample Weight:*	**1,0000**

Chromatogram

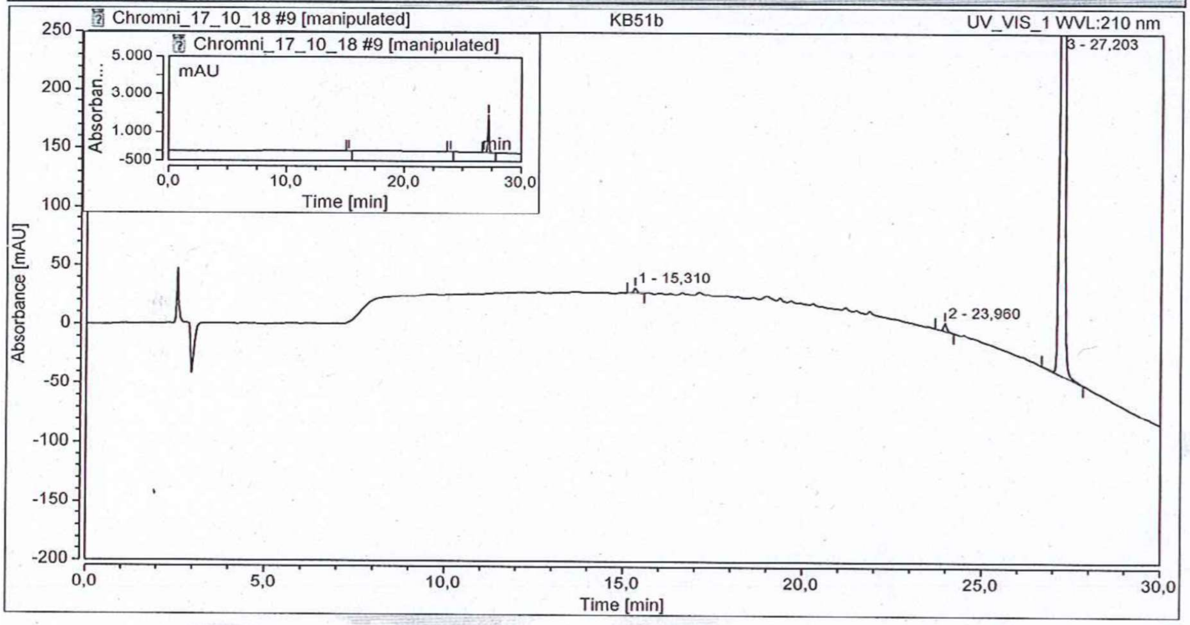

Integration Results

No.	Peak Name	Retention Time min	Area mAU*min	Height mAU	Relative Area %	Relative Height %	Amount n.a.
1		15,310	0,347	4,210	0,17	0,21	n.a.
2		23,960	0,673	6,587	0,34	0,33	n.a.
3		27,203	199,067	1969,465	99,49	99,45	n.a.
**Total:**			**200,087**	**1980,262**	**100,00**	**100,00**	

HPLC trace of **17**

Chromatogram and Results

Injection Details

Injection Name:	KB54a2.1	*Run Time (min):*	30,00
Vial Number:	GA2	*Injection Volume:*	5,00
Injection Type:	Unknown	*Channel:*	UV_VIS_1
Calibration Level:		*Wavelength:*	210,0
Instrument Method:	Chromni	*Bandwidth:*	n.a.
Processing Method:	Chromni Processing Method	*Dilution Factor:*	1,0000
Injection Date/Time:	27.Sep.17 17:21	*Sample Weight:*	1,0000

Chromatogram

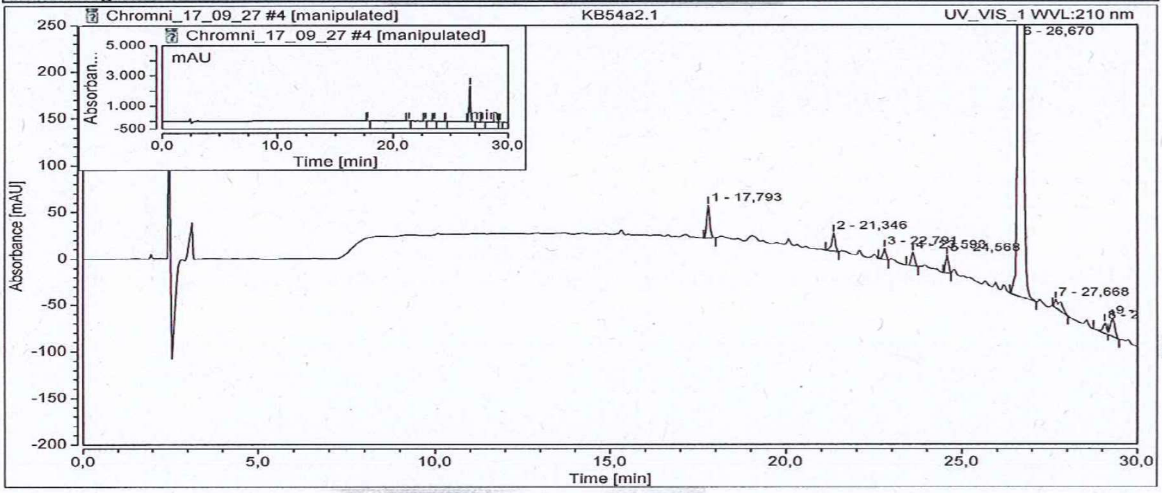

Integration Results

No.	Peak Name	Retention Time min	Area mAU*min	Height mAU	Relative Area %	Relative Height %	Amount n.a.
1		17,793	3,335	35,016	1,03	1,42	n.a.
2		21,346	2,194	19,207	0,68	0,78	n.a.
3		22,791	1,176	11,674	0,36	0,47	n.a.
4		23,593	1,636	14,448	0,51	0,58	n.a.
5		24,568	1,789	18,776	0,55	0,76	n.a.
6		26,670	305,953	2332,355	94,83	94,35	n.a.
7		27,668	2,338	9,266	0,72	0,37	n.a.
8		29,068	1,332	11,352	0,41	0,46	n.a.
9		29,293	2,873	20,030	0,89	0,81	n.a.
Total:			322,626	2472,124	100,00	100,00	

HPCL trace of **18**

Chromatogram and Results

Injection Details

Injection Name:	**KB55**	*Run Time (min):*	**40,00**
Vial Number:	**GA2**	*Injection Volume:*	**5,00**
Injection Type:	**Unknown**	*Channel:*	**UV_VIS_1**
Calibration Level:		*Wavelength:*	**210,0**
Instrument Method:	**Chromni - steiler**	*Bandwidth:*	**n.a.**
Processing Method:	**Chromni Processing Method - steiler**	*Dilution Factor:*	**1,0000**
Injection Date/Time:	**14.Nov.19 08:29**	*Sample Weight:*	**1,0000**

Chromatogram

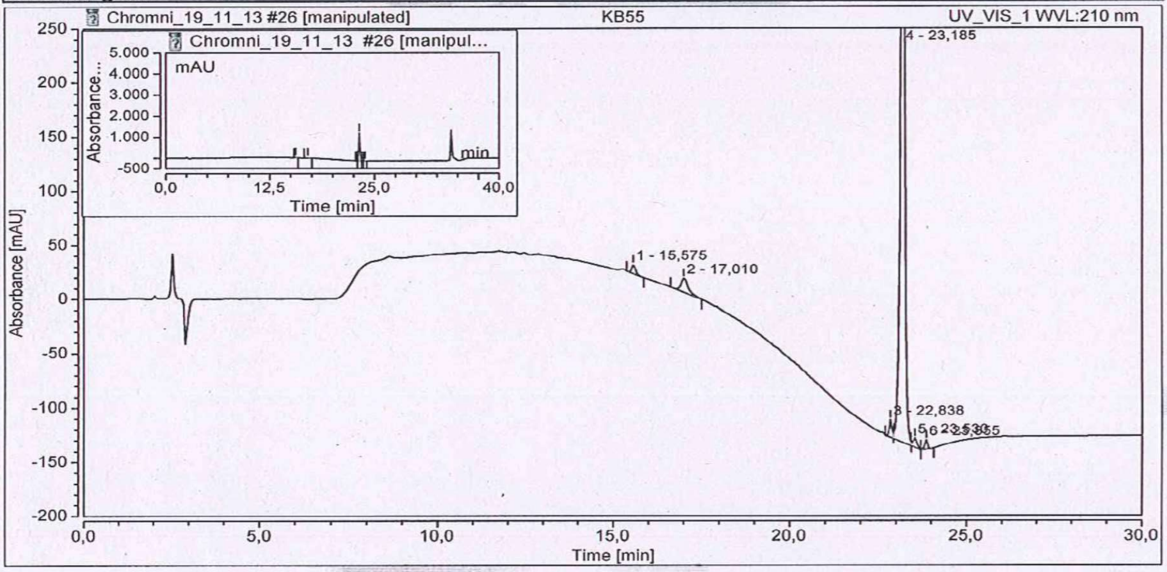

Integration Results

No.	Peak Name	Retention Time min	Area mAU*min	Height mAU	Relative Area %	Relative Height %	Amount n.a.
1		15,575	0,968	8,382	0,74	0,61	n.a.
2		17,010	3,172	13,048	2,41	0,95	n.a.
3		22,838	1,344	16,168	1,02	1,18	n.a.
4		23,185	123,955	1315,203	94,38	96,01	n.a.
5		23,530	1,192	8,694	0,91	0,63	n.a.
6		23,855	0,703	8,436	0,54	0,62	n.a.
**Total:**			**131,333**	**1369,930**	**100,00**	**100,00**	

HPCL trace of **19**

Chromatogram and Results

Injection Details

Injection Name:	**KB63a**	*Run Time (min):*	**30,00**
Vial Number:	**GA3**	*Injection Volume:*	**5,00**
Injection Type:	**Unknown**	*Channel:*	**UV_VIS_1**
Calibration Level:		*Wavelength:*	**210,0**
Instrument Method:	**Chromni**	*Bandwidth:*	**n.a.**
Processing Method:	**Chromni Processing Method**	*Dilution Factor:*	**1,0000**
Injection Date/Time:	**22.Aug.19 21:10**	*Sample Weight:*	**1,0000**

Chromatogram

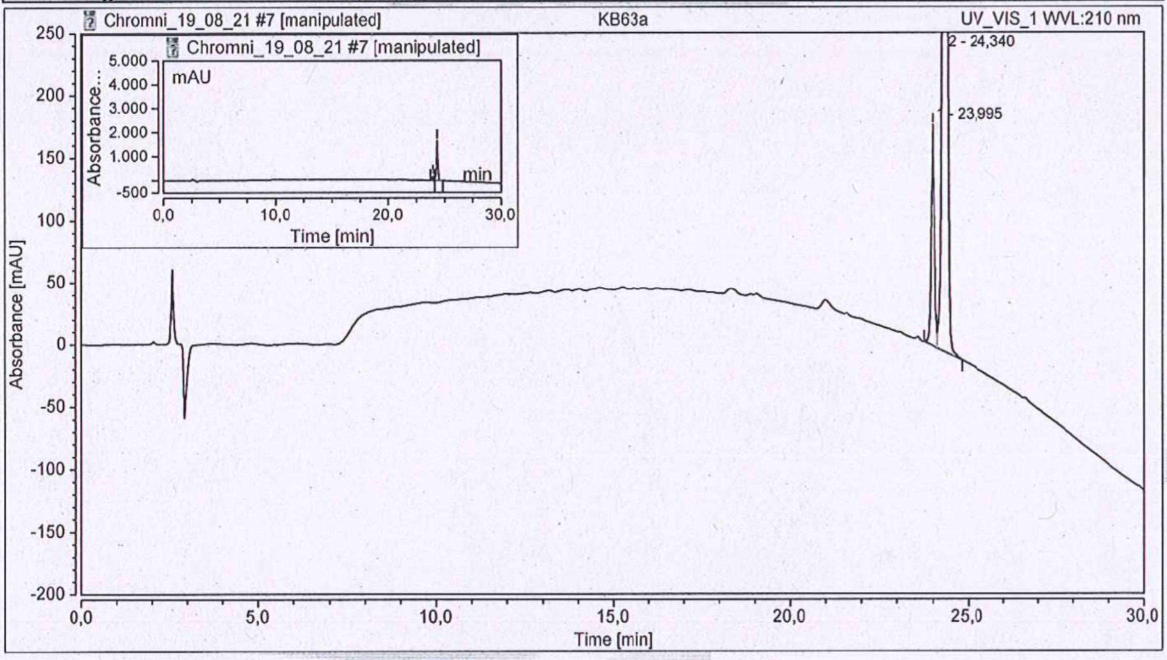

Integration Results

No.	Peak Name	Retention Time min	Area mAU*min	Height mAU	Relative Area %	Relative Height %	Amount n.a.
1		23,995	19,712	177,240	9,59	9,72	n.a.
2		24,340	185,891	1647,045	90,41	90,28	n.a.
**Total:**			**205,603**	**1824,285**	**100,00**	**100,00**	

HPLC trace of **20**

Chromatogram and Results

Injection Details

Injection Name:	**KB109**	*Run Time (min):*	**30,00**
Vial Number:	**GC5**	*Injection Volume:*	**5,00**
Injection Type:	**Unknown**	*Channel:*	**UV_VIS_1**
Calibration Level:		*Wavelength:*	**210,0**
Instrument Method:	**Chromni**	*Bandwidth:*	**n.a.**
Processing Method:	**Chromni Processing Method**	*Dilution Factor:*	**1,0000**
Injection Date/Time:	**28.Feb.19 04:07**	*Sample Weight:*	**1,0000**

Chromatogram

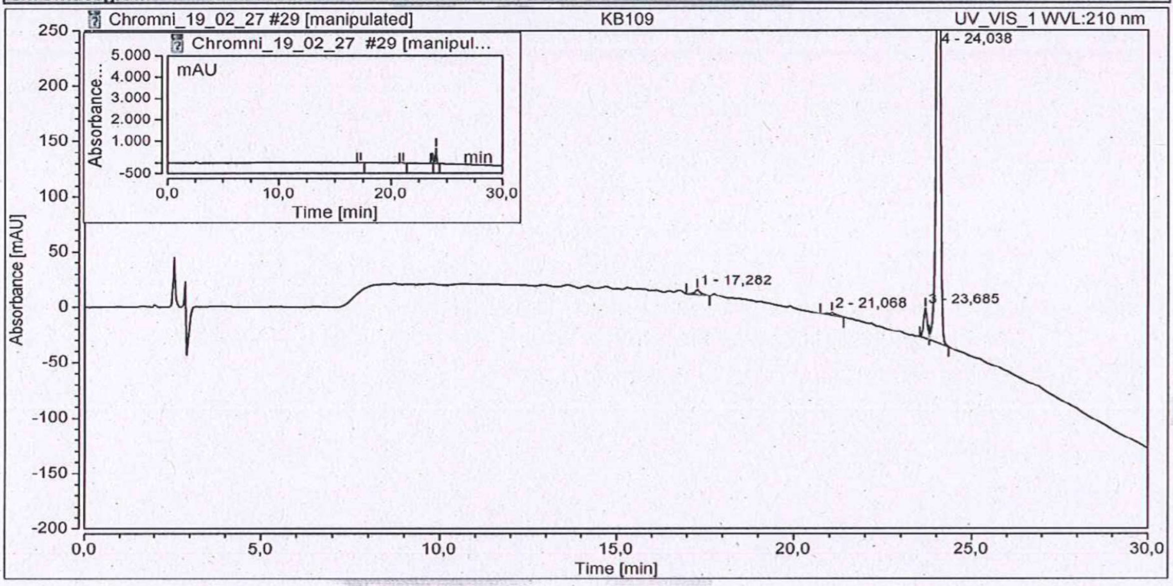

Integration Results

No.	Peak Name	Retention Time<br>min	Area<br>mAU*min	Height<br>mAU	Relative Area<br>%	Relative Height<br>%	Amount<br>n.a.
1		17,282	0,874	4,627	1,04	0,63	n.a.
2		21,068	0,577	2,656	0,69	0,36	n.a.
3		23,685	3,035	27,169	3,63	3,71	n.a.
4		24,038	79,210	697,461	94,64	95,29	n.a.
**Total:**			**83,696**	**731,913**	**100,00**	**100,00**	

HPLC trace of **21**

Chromatogram and Results

Injection Details

Injection Name:	**KB85b1**	*Run Time (min):*	**40,00**
Vial Number:	**GA8**	*Injection Volume:*	**5,00**
Injection Type:	**Unknown**	*Channel:*	**UV_VIS_1**
Calibration Level:		*Wavelength:*	**210,0**
Instrument Method:	**Chromni - steiler**	*Bandwidth:*	**n.a.**
Processing Method:	**Chromni Processing Method - steiler**	*Dilution Factor:*	**1,0000**
Injection Date/Time:	**19.Jul.18 13:19**	*Sample Weight:*	**1,0000**

Chromatogram

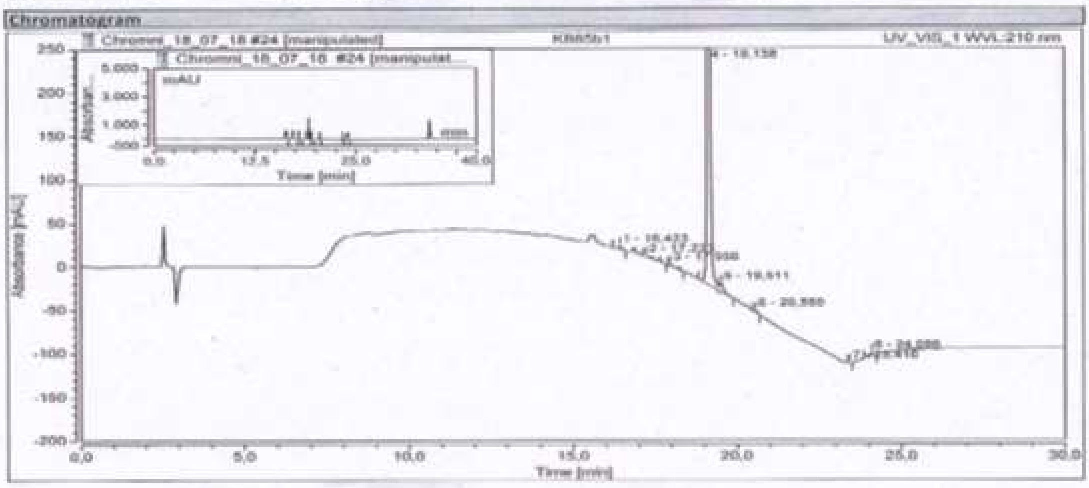

Integration Results

No.	Peak Name	Retention Time min	Area mAU*min	Height mAU	Relative Area %	Relative Height %	Amount n.a.
1		16,423	0,321	4,001	0,37	0,41	n.a.
2		17,223	0,910	1,764	1,05	0,18	n.a.
3		17,956	0,500	2,116	0,58	0,21	n.a.
4		19,138	82,914	963,198	95,57	97,28	n.a.
5		19,511	1,338	10,241	1,54	1,03	n.a.
6		20,560	0,239	3,069	0,27	0,31	n.a.
7		23,416	0,034	0,565	0,04	0,06	n.a.
8		24,086	0,495	5,118	0,57	0,52	n.a.
Total:			86,756	990,131	100,00	100,00	

HPLC trace of **22**

Chromatogram and Results

Injection Details

Injection Name:	KB58a2	*Run Time (min):*	40,00
Vial Number:	GB8	*Injection Volume:*	5,00
Injection Type:	Unknown	*Channel:*	UV_VIS_1
Calibration Level:		*Wavelength:*	210,0
Instrument Method:	Chromni - steiler	*Bandwidth:*	n.a.
Processing Method:	Chromni Processing Method	*Dilution Factor:*	1,0000
Injection Date/Time:	30.Nov.17 20:20	*Sample Weight:*	1,0000

Chromatogram

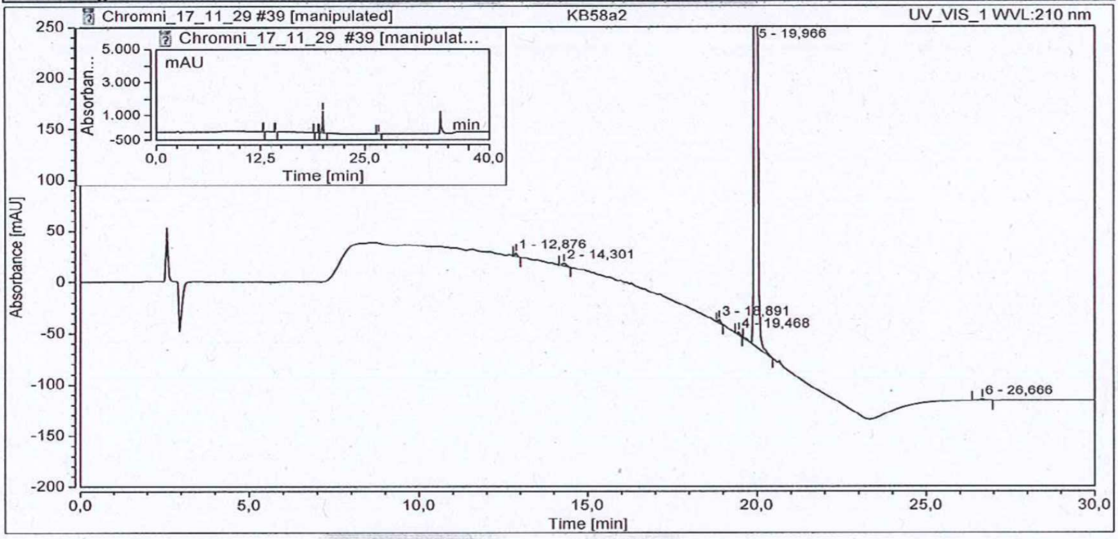

Integration Results

No.	Peak Name	Retention Time min	Area mAU*min	Height mAU	Relative Area %	Relative Height %	Amount n.a.
1		12,876	0,226	3,253	0,20	0,25	n.a.
2		14,301	0,390	2,571	0,35	0,20	n.a.
3		18,891	0,261	3,204	0,24	0,25	n.a.
4		19,468	0,207	2,886	0,19	0,22	n.a.
5		19,966	109,518	1293,891	98,77	98,98	n.a.
6		26,666	0,285	1,356	0,26	0,10	n.a.
**Total:**			**110,887**	**1307,160**	**100,00**	**100,00**	

HPLC trace of **23**

Chromatogram and Results

Injection Details

Injection Name:	KB57a2	*Run Time (min):*	40,00
Vial Number:	GC1	*Injection Volume:*	5,00
Injection Type:	Unknown	*Channel:*	UV_VIS_1
Calibration Level:		*Wavelength:*	210,0
Instrument Method:	Chromni - steiler	*Bandwidth:*	n.a.
Processing Method:	Chromni Processing Method	*Dilution Factor:*	1,0000
Injection Date/Time:	30.Nov.17 21:01	*Sample Weight:*	1,0000

Chromatogram

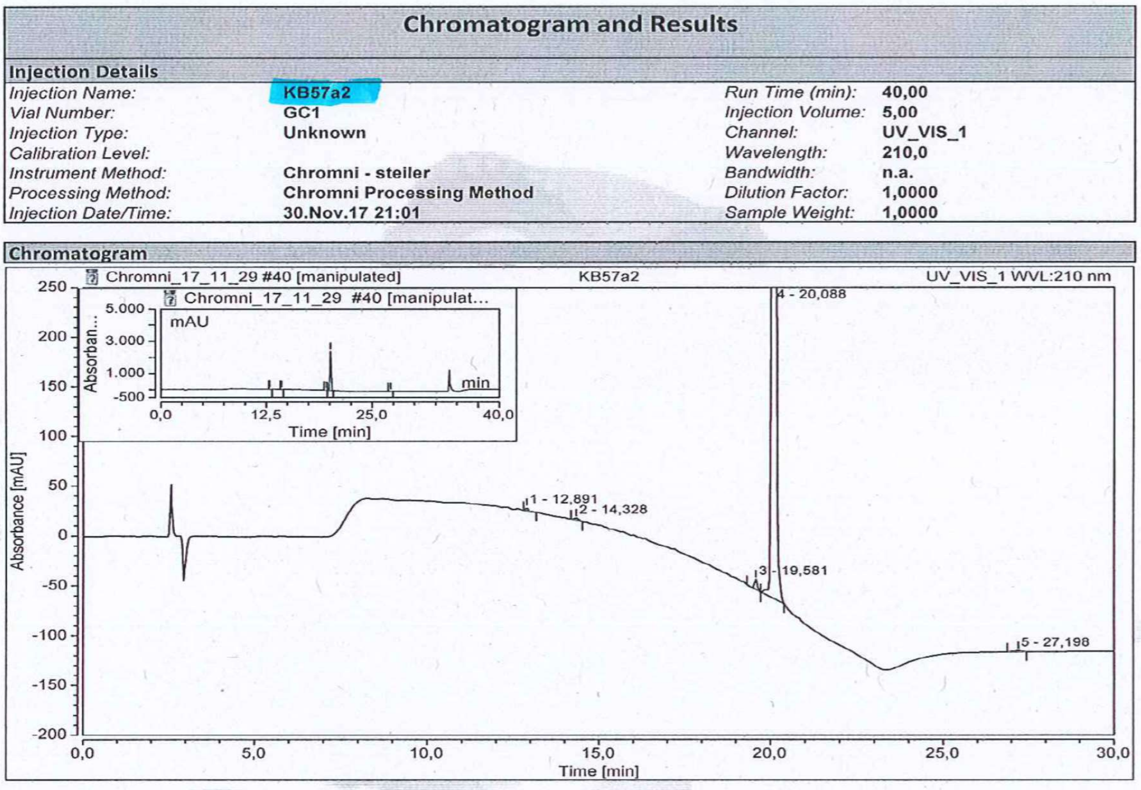

Integration Results

No.	Peak Name	Retention Time min	Area mAU*min	Height mAU	Relative Area %	Relative Height %	Amount n.a.
1		12,891	0,194	3,392	0,07	0,14	n.a.
2		14,328	0,369	2,146	0,14	0,09	n.a.
3		19,581	0,856	10,260	0,32	0,42	n.a.
4		20,088	264,507	2422,964	99,37	99,31	n.a.
5		27,198	0,255	1,149	0,10	0,05	n.a.
**Total:**			**266,181**	**2439,910**	**100,00**	**100,00**	

HPLC trace of **24**

Chromatogram and Results

Injection Details

Injection Name:	**KB59**	*Run Time (min):*	**40,00**
Vial Number:	**GA4**	*Injection Volume:*	**5,00**
Injection Type:	**Unknown**	*Channel:*	**UV_VIS_1**
Calibration Level:		*Wavelength:*	**210,0**
Instrument Method:	**Chromni - steiler**	*Bandwidth:*	**n.a.**
Processing Method:	**Chromni Processing Method - steiler**	*Dilution Factor:*	**1,0000**
Injection Date/Time:	**29.Okt.19 02:47**	*Sample Weight:*	**1,0000**

Chromatogram

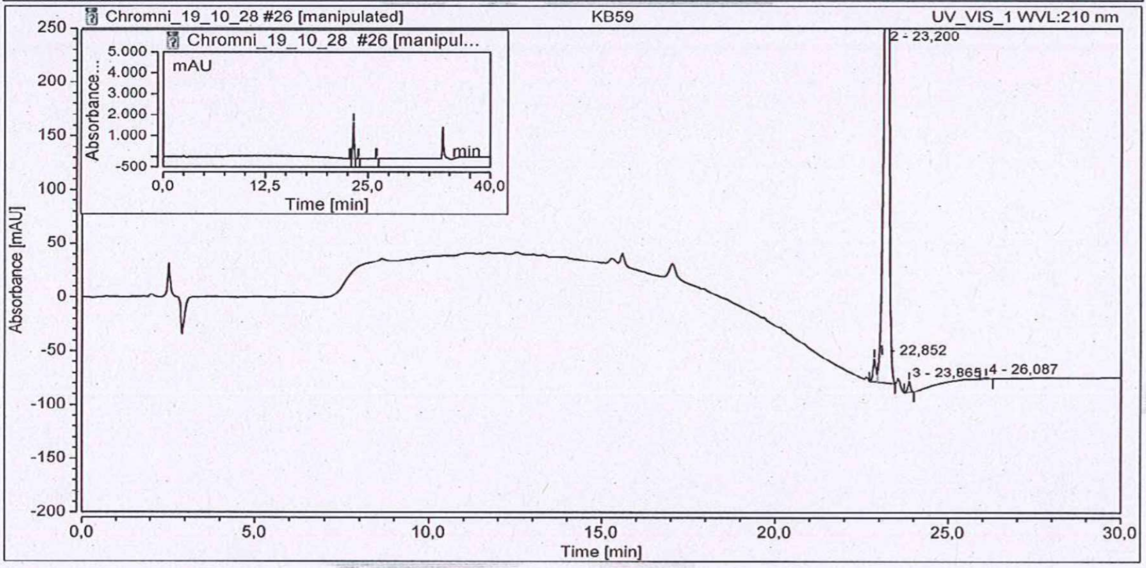

Integration Results

No.	Peak Name	Retention Time min	Area mAU*min	Height mAU	Relative Area %	Relative Height %	Amount n.a.
1		22,852	1,961	20,993	1,11	1,22	n.a.
2		23,200	173,580	1693,365	98,27	98,15	n.a.
3		23,865	0,956	10,031	0,54	0,58	n.a.
4		26,087	0,134	0,907	0,08	0,05	n.a.
**Total:**			**176,630**	**1725,296**	**100,00**	**100,00**	

HPLC trace of **25**

Chromatogram and Results

Injection Details

Injection Name:	46a4	*Run Time (min):*	30,00
Vial Number:	GB5	*Injection Volume:*	5,00
Injection Type:	Unknown	*Channel:*	UV_VIS_1
Calibration Level:		*Wavelength:*	210,0
Instrument Method:	Chromni	*Bandwidth:*	n.a.
Processing Method:	Chromni Processing Method	*Dilution Factor:*	1,0000
Injection Date/Time:	30.Nov.17 03:59	*Sample Weight:*	1,0000

Chromatogram

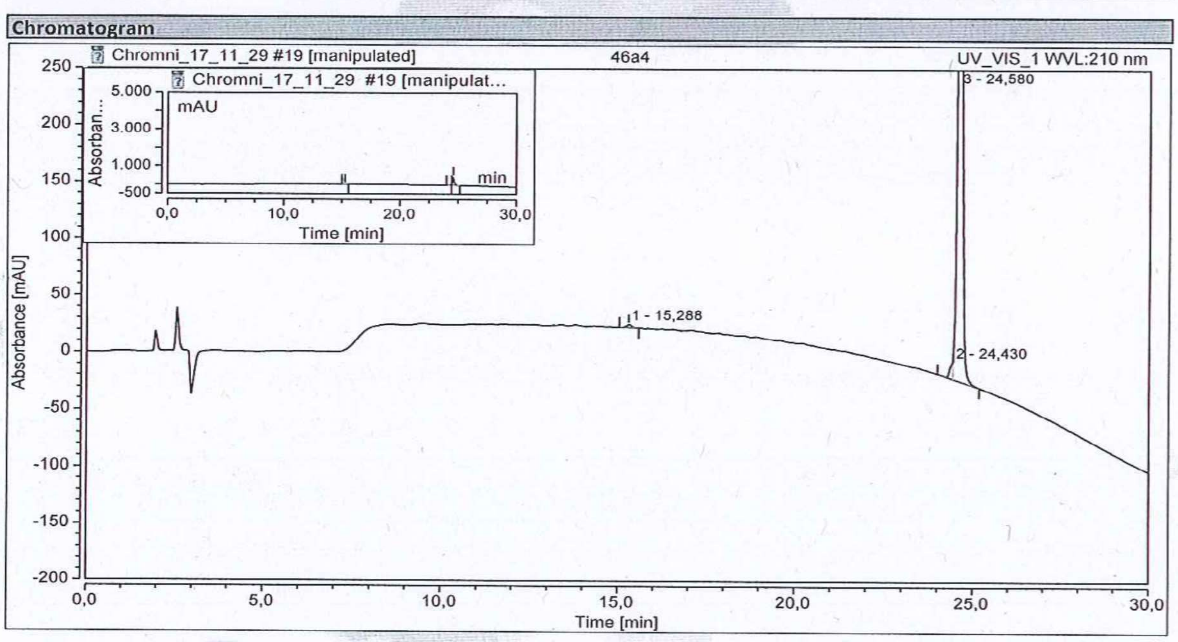

Integration Results

No.	Peak Name	Retention Time min	Area mAU*min	Height mAU	Relative Area %	Relative Height %	Amount n.a.
1		15,288	0,396	2,330	0,51	0,50	n.a.
2		24,430	1,318	14,245	1,69	3,06	n.a.
3		24,580	76,447	448,556	97,81	96,44	n.a.
**Total:**			**78,160**	**465,132**	**100,00**	**100,00**	

HPLC trace of **26**

Chromatogram and Results

Injection Details

Injection Name:	**KB111.2**	*Run Time (min):*	**30,00**
Vial Number:	**GA3**	*Injection Volume:*	**5,00**
Injection Type:	**Unknown**	*Channel:*	**UV_VIS_1**
Calibration Level:		*Wavelength:*	**210,0**
Instrument Method:	**Chromni**	*Bandwidth:*	**n.a.**
Processing Method:	**Chromni Processing Method**	*Dilution Factor:*	**1,0000**
Injection Date/Time:	**04.Apr.19 03:40**	*Sample Weight:*	**1,0000**

Chromatogram

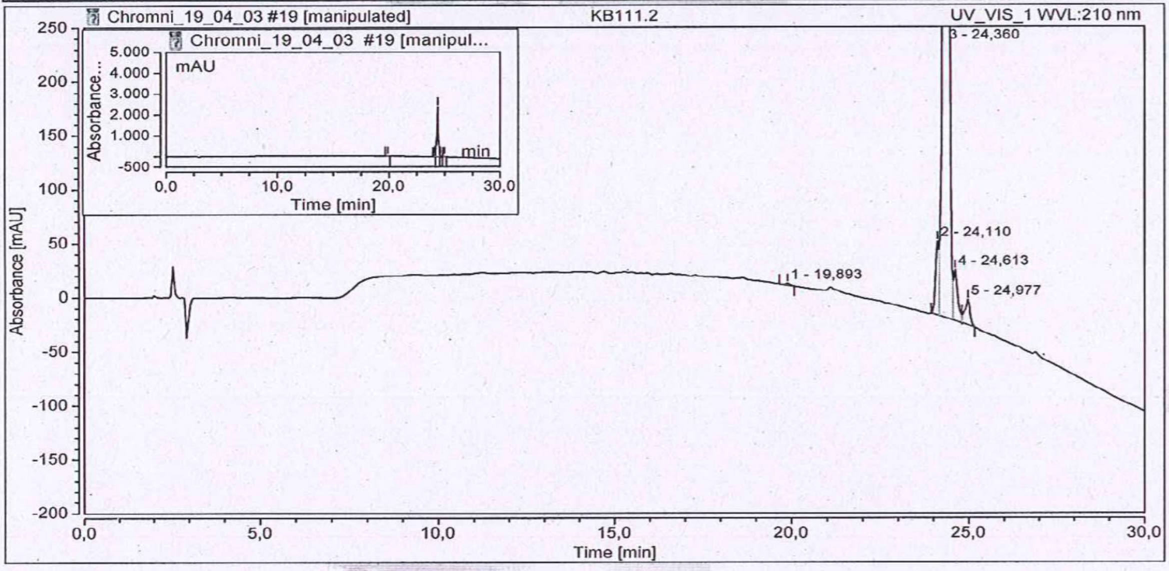

Integration Results

No.	Peak Name	Retention Time min	Area mAU*min	Height mAU	Relative Area %	Relative Height %	Amount n.a.
1		19,893	0,184	1,544	0,05	0,06	n.a.
2		24,110	7,154	67,898	2,07	2,70	n.a.
3		24,360	327,973	2379,001	94,81	94,46	n.a.
4		24,613	6,677	46,970	1,93	1,86	n.a.
5		24,977	3,928	23,178	1,14	0,92	n.a.
**Total:**			**345,916**	**2518,591**	**100,00**	**100,00**	

8. Absorption and emission spectra of compounds 15 – 20

Absorption and emission spectra of **15**

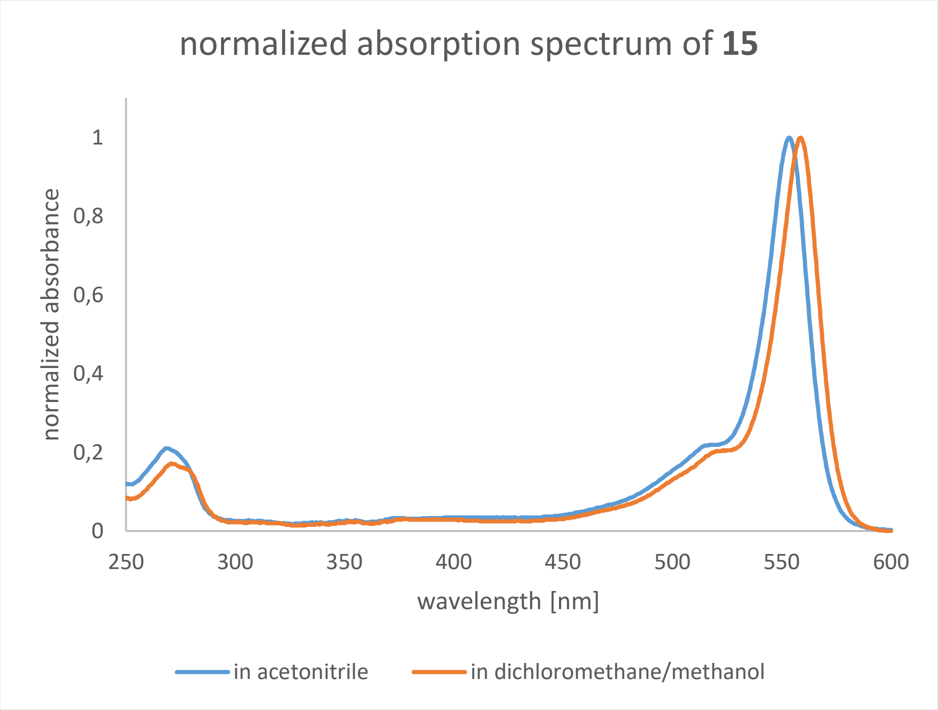

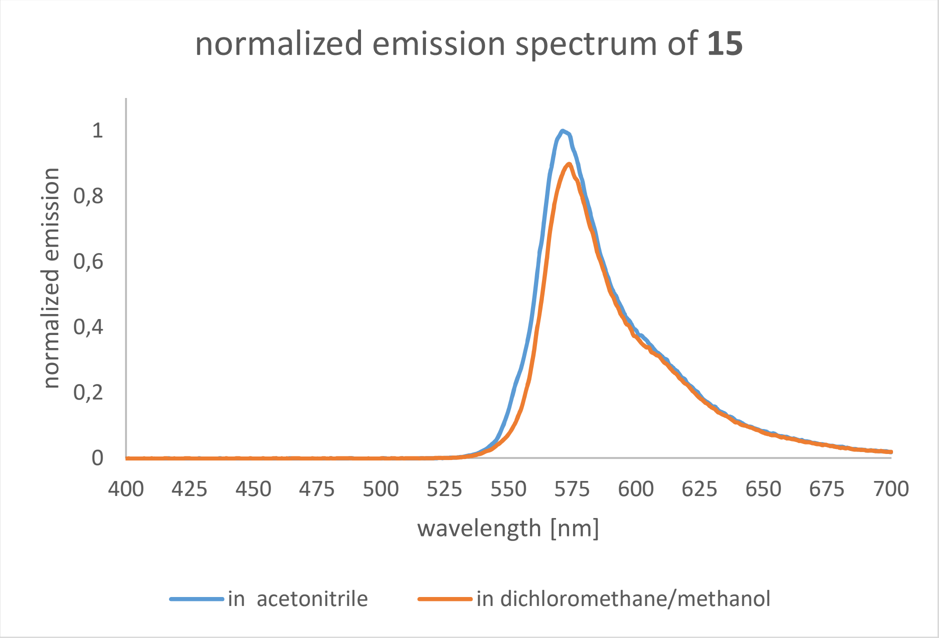

Absorption and emission spectra of **16**

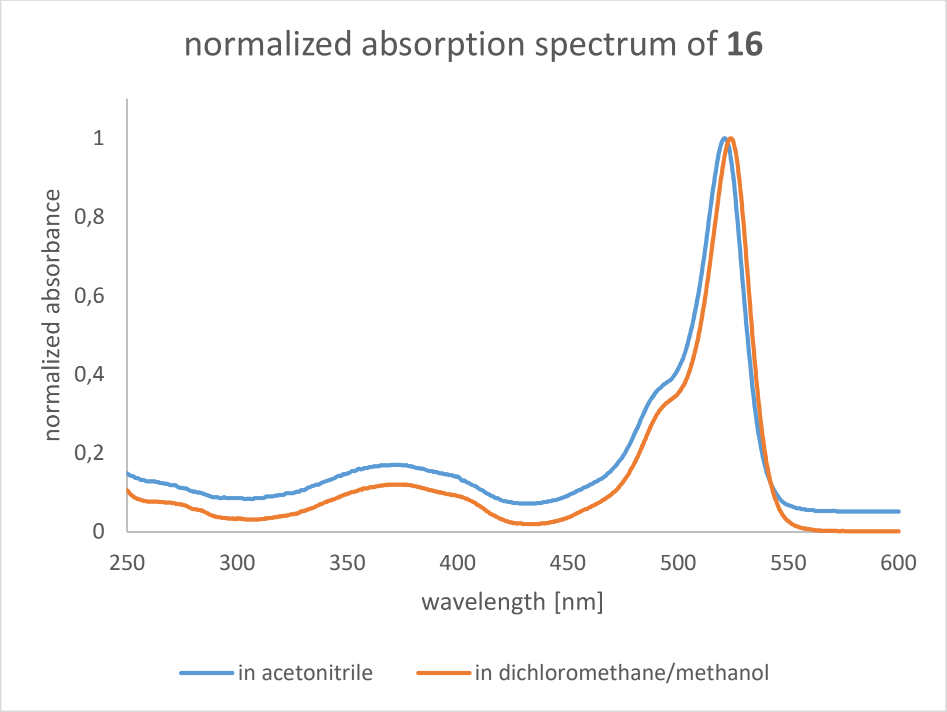

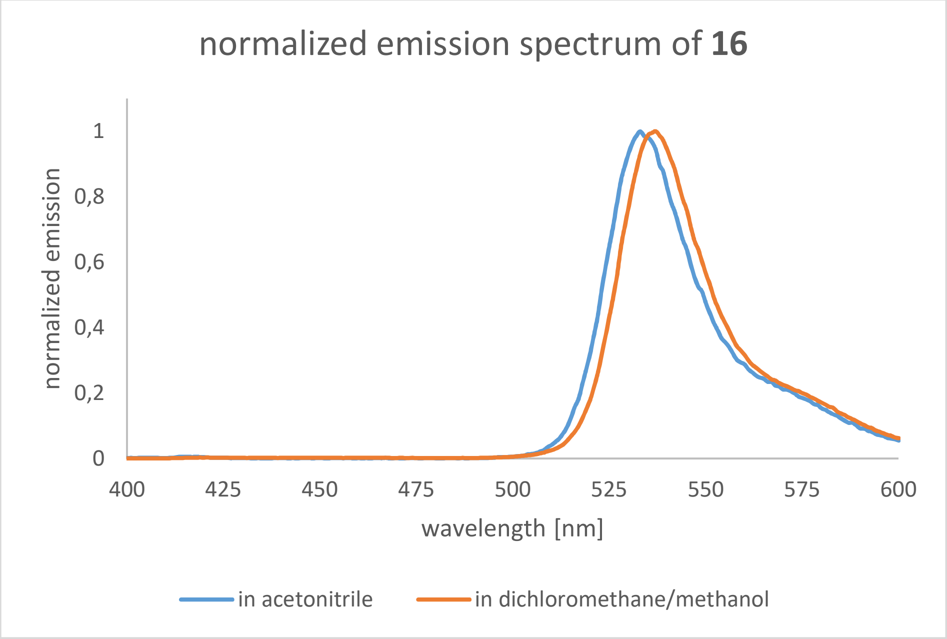

Absorption and emission spectra of **17**

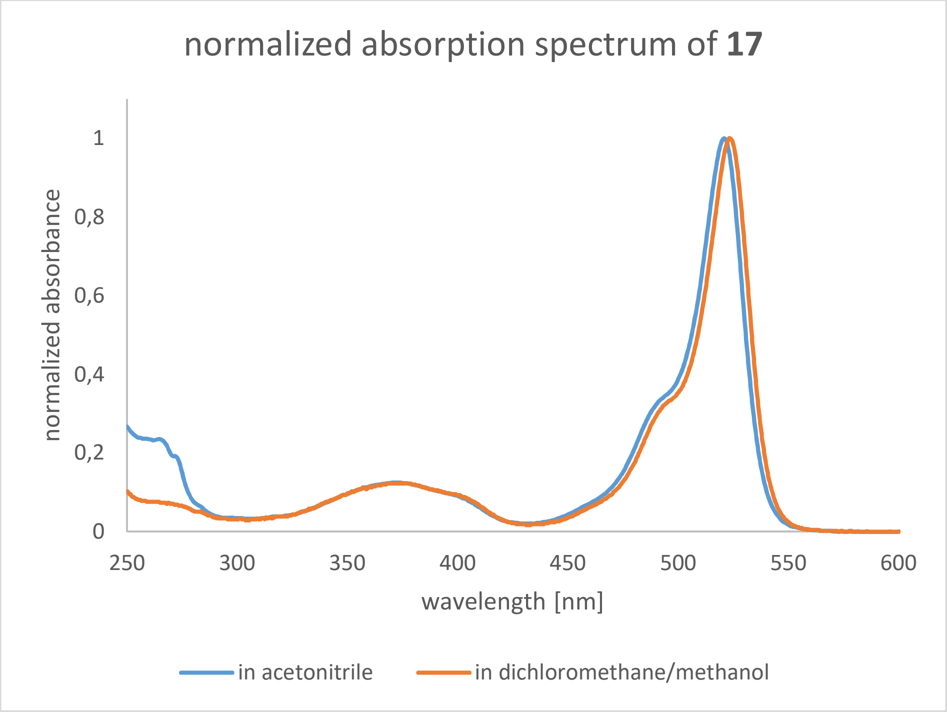

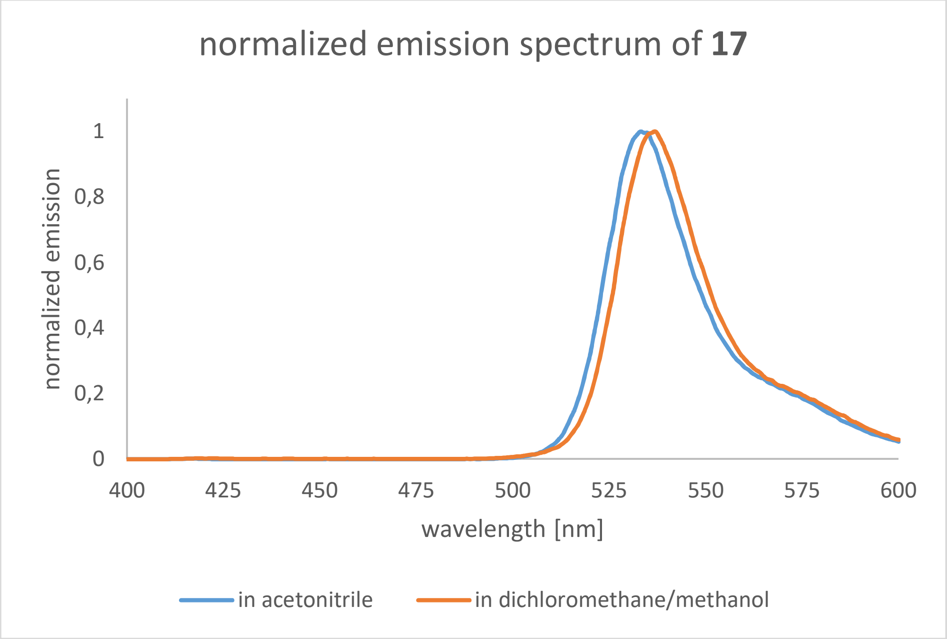

Absorption and emission spectra of **18**

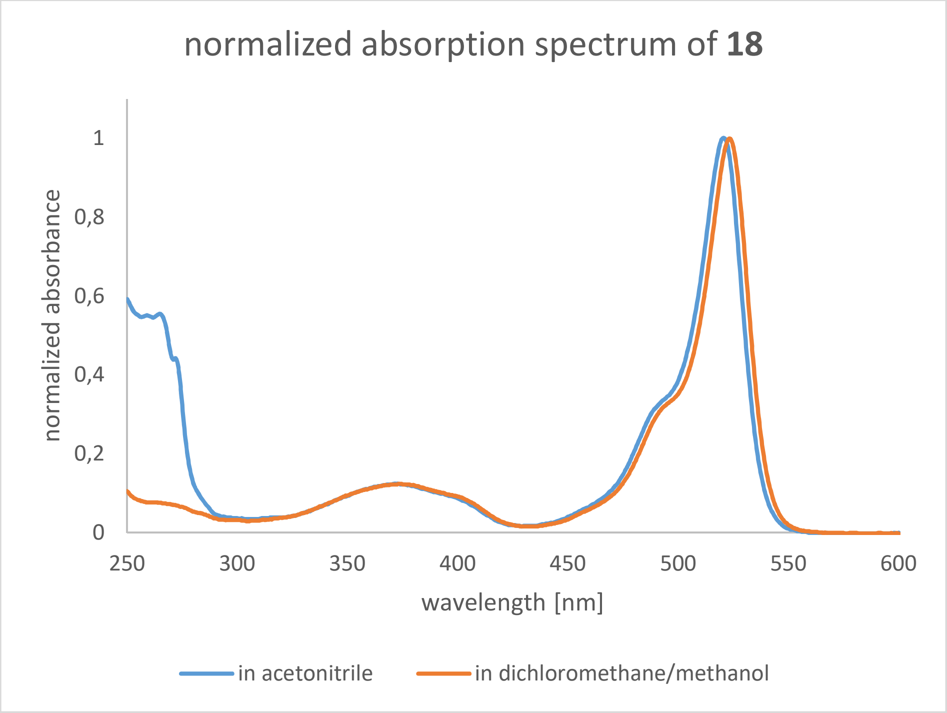

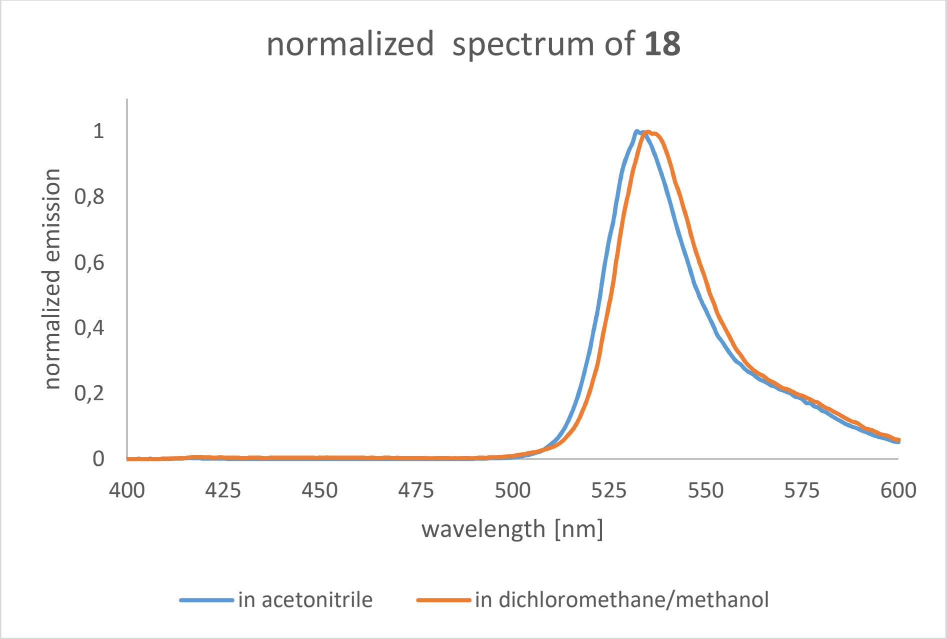

Absorption and emission spectra of **19**

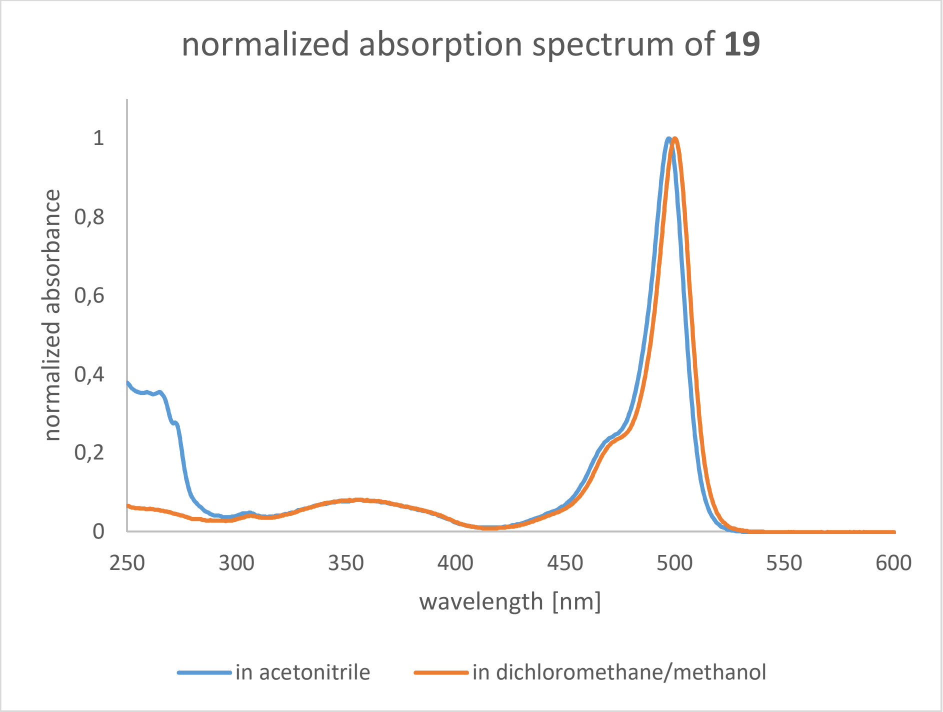

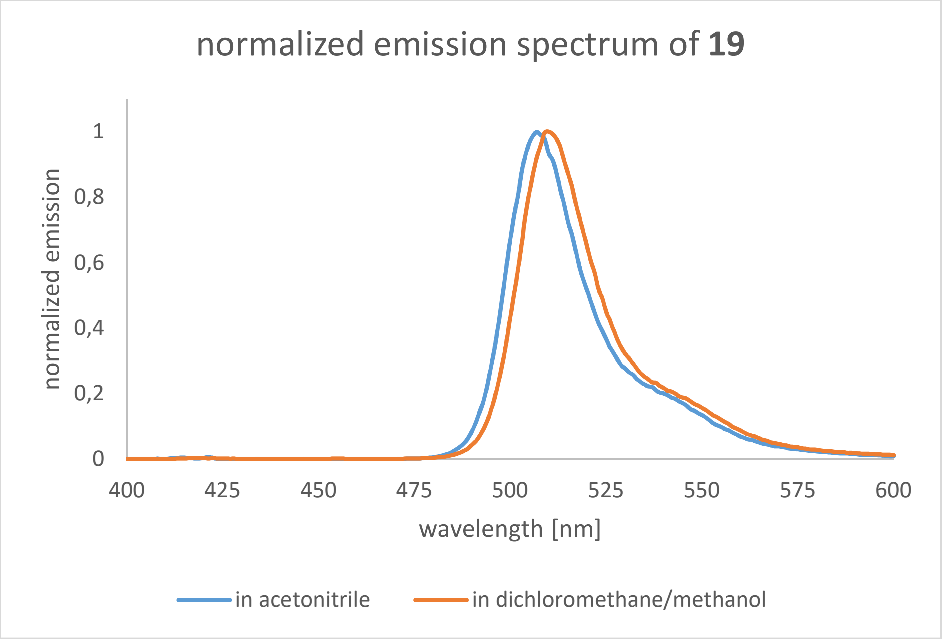

Absorption and emission spectra of **20**

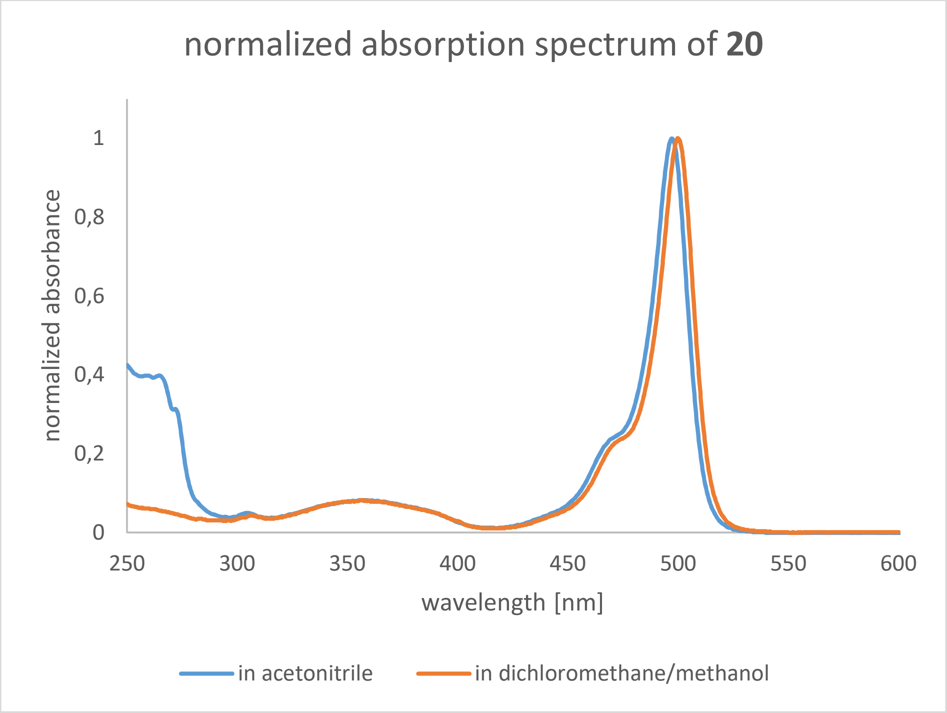

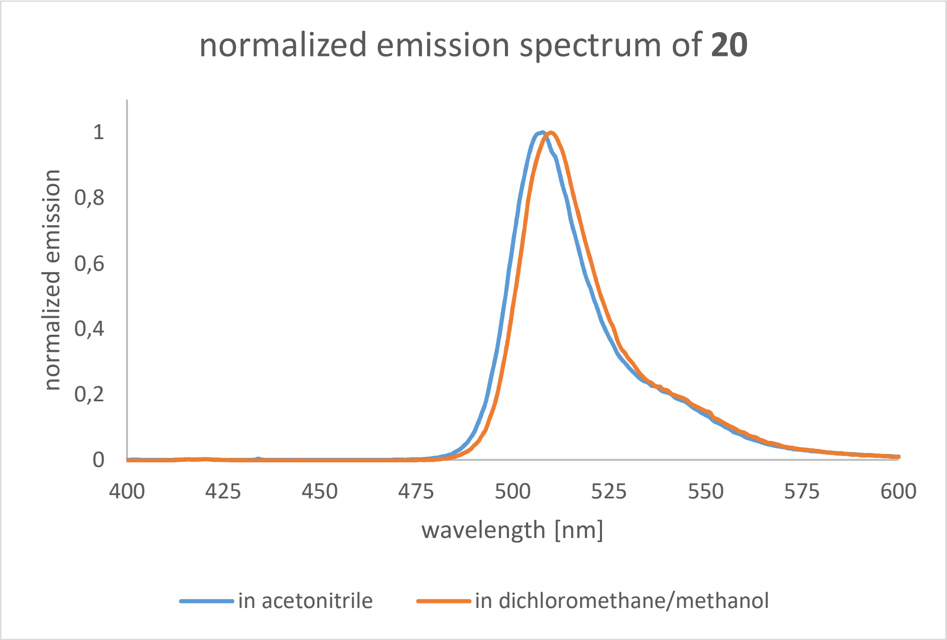

9. Photophysical data of compounds 15 - 20

Photoluminescence quantum yields (Φ_L)

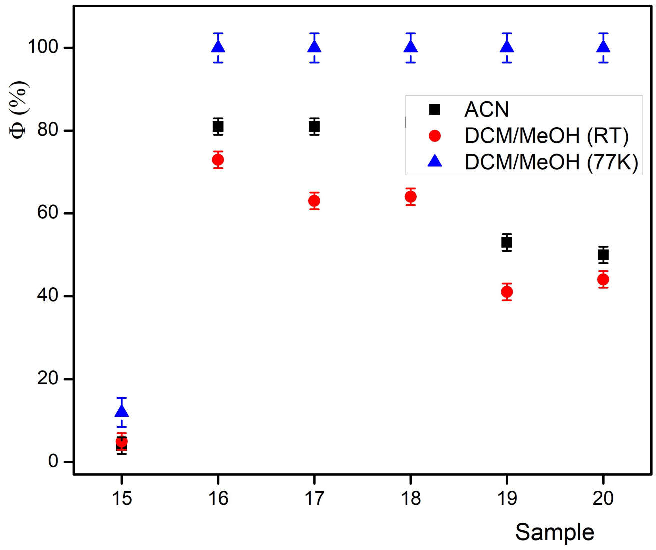

Amplitude-Weighted Average Lifetimes ⟨τ⟩

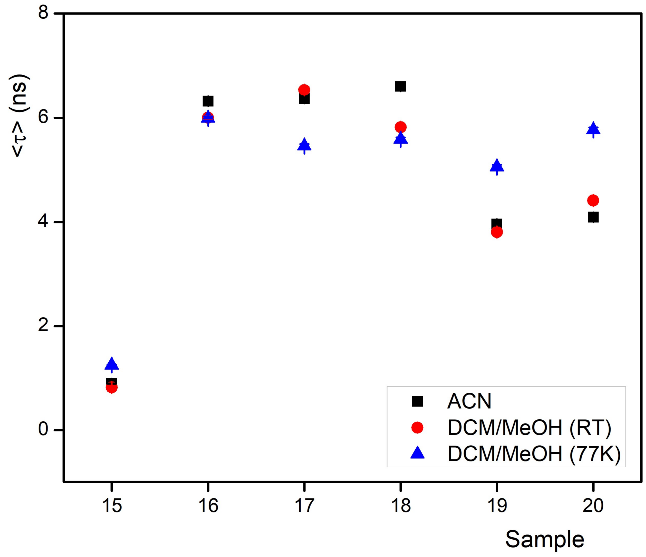

Radiative deactivation rate constants (k_r)

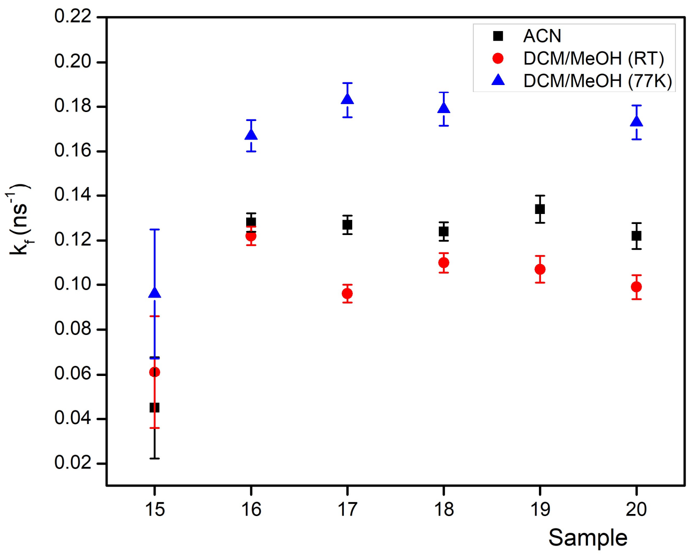

Non-radiative deactivation rate constants (k_{nr})

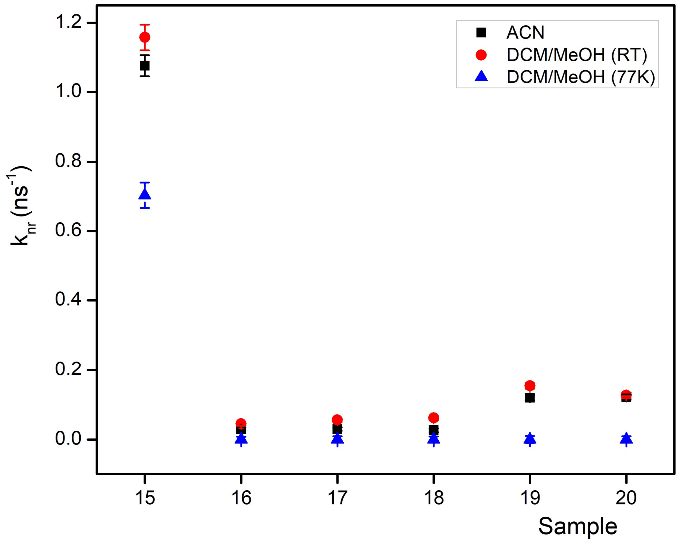

Time-resolved photoluminescence decay (including the instrument response function in red and the residuals of the fit) of compound **15** in CH_3CN (top), CH_2Cl_2/CH_3OH at rt (center) and CH_2Cl_2/CH_3OH at 77 K (bottom).

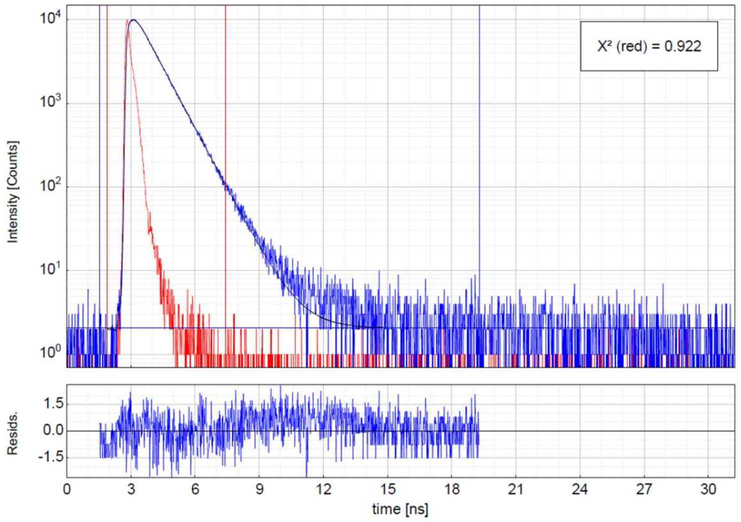

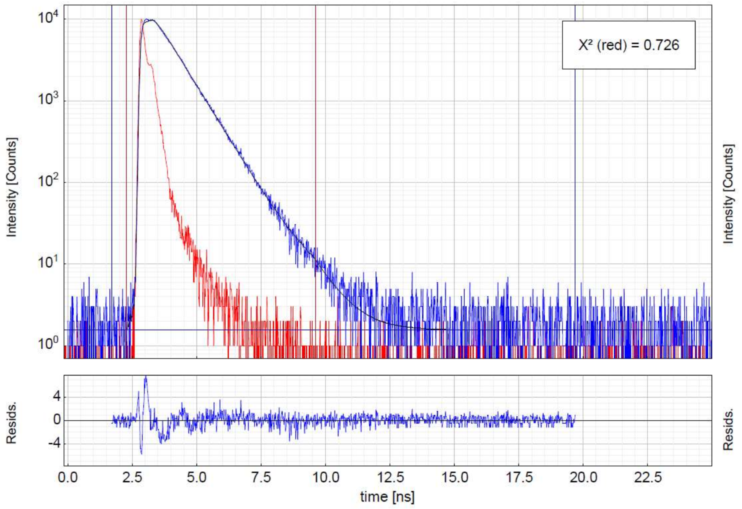

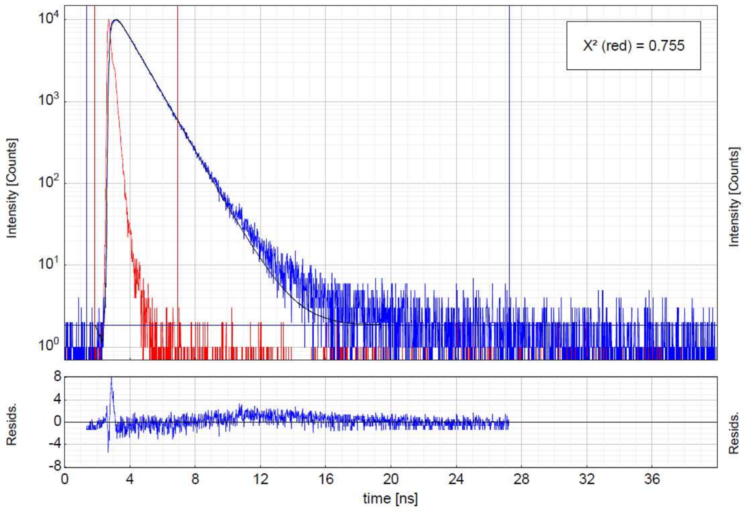

Time-resolved photoluminescence decay (including the instrument response function in red and the residuals of the fit) of compound **16** in CH_3CN (top), CH_2Cl_2/CH_3OH at rt (center) and CH_2Cl_2/CH_3OH at 77 K (bottom).

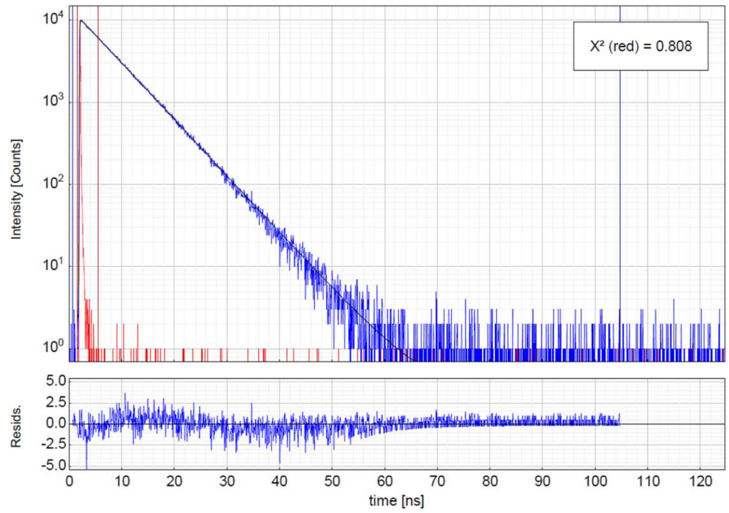

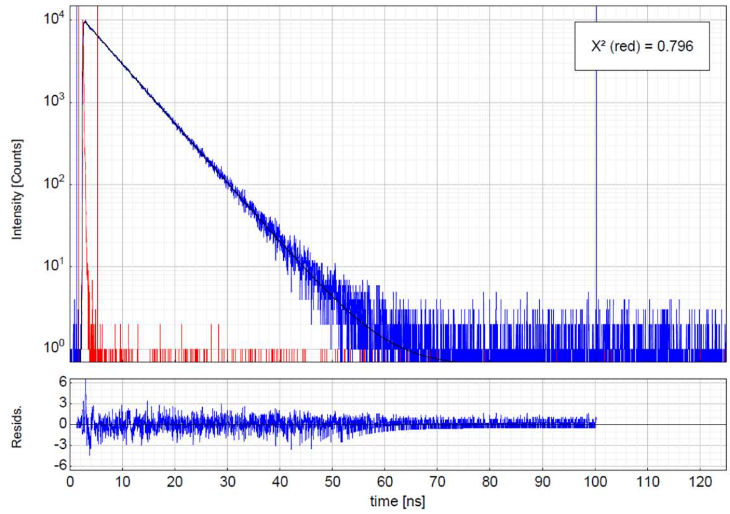

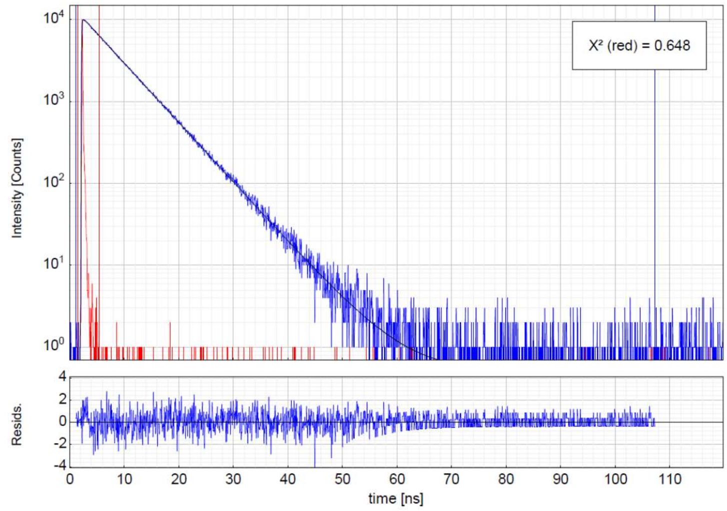

Time-resolved photoluminescence decay (including the instrument response function in red and the residuals of the fit) of compound **17** in CH_3CN (top), CH_2Cl_2/CH_3OH at rt (center) and CH_2Cl_2/CH_3OH at 77 K (bottom).

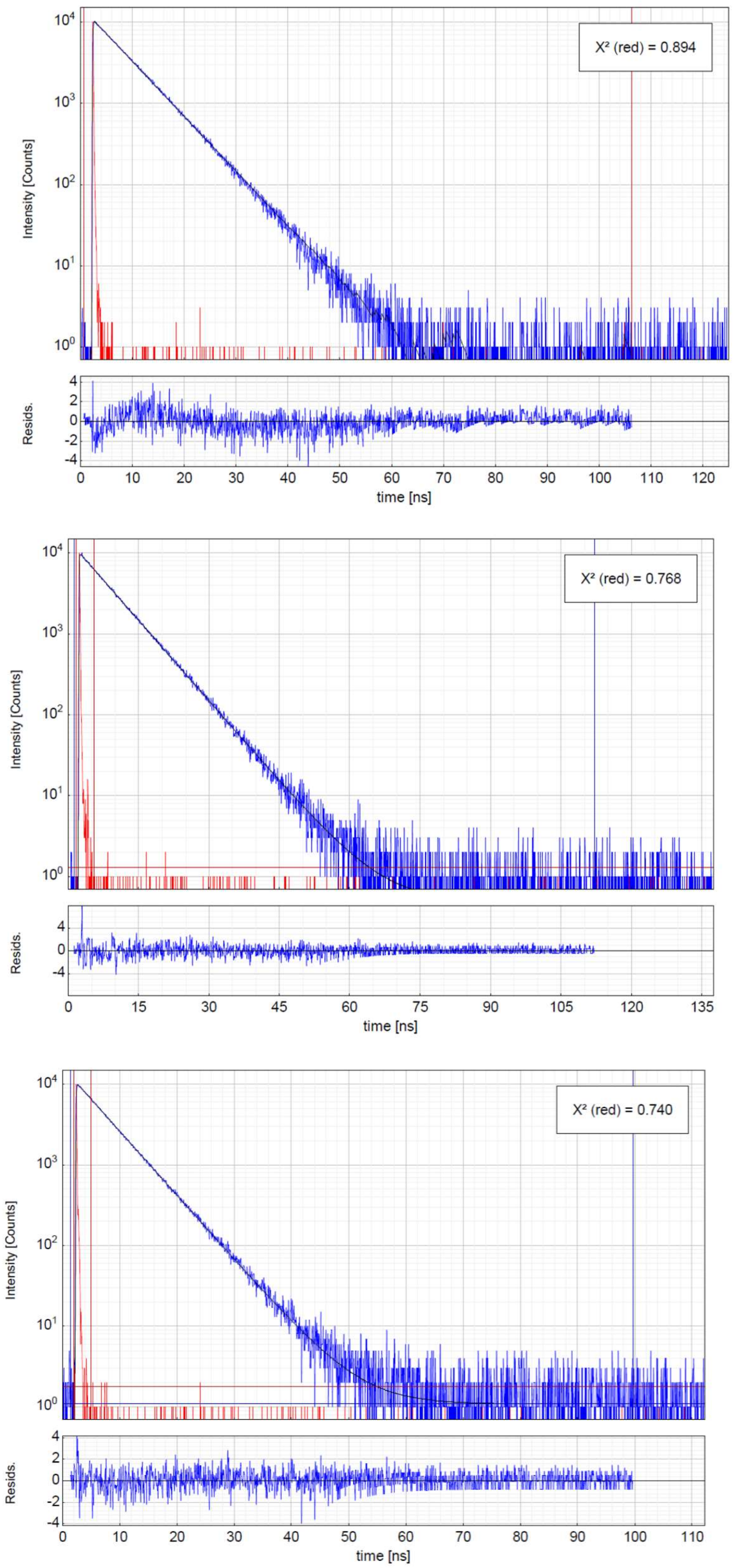

Time-resolved photoluminescence decay (including the instrument response function in red and the residuals of the fit) of compound **18** in CH_3CN (top), CH_2Cl_2/CH_3OH at rt (center) and CH_2Cl_2/CH_3OH at 77 K (bottom).

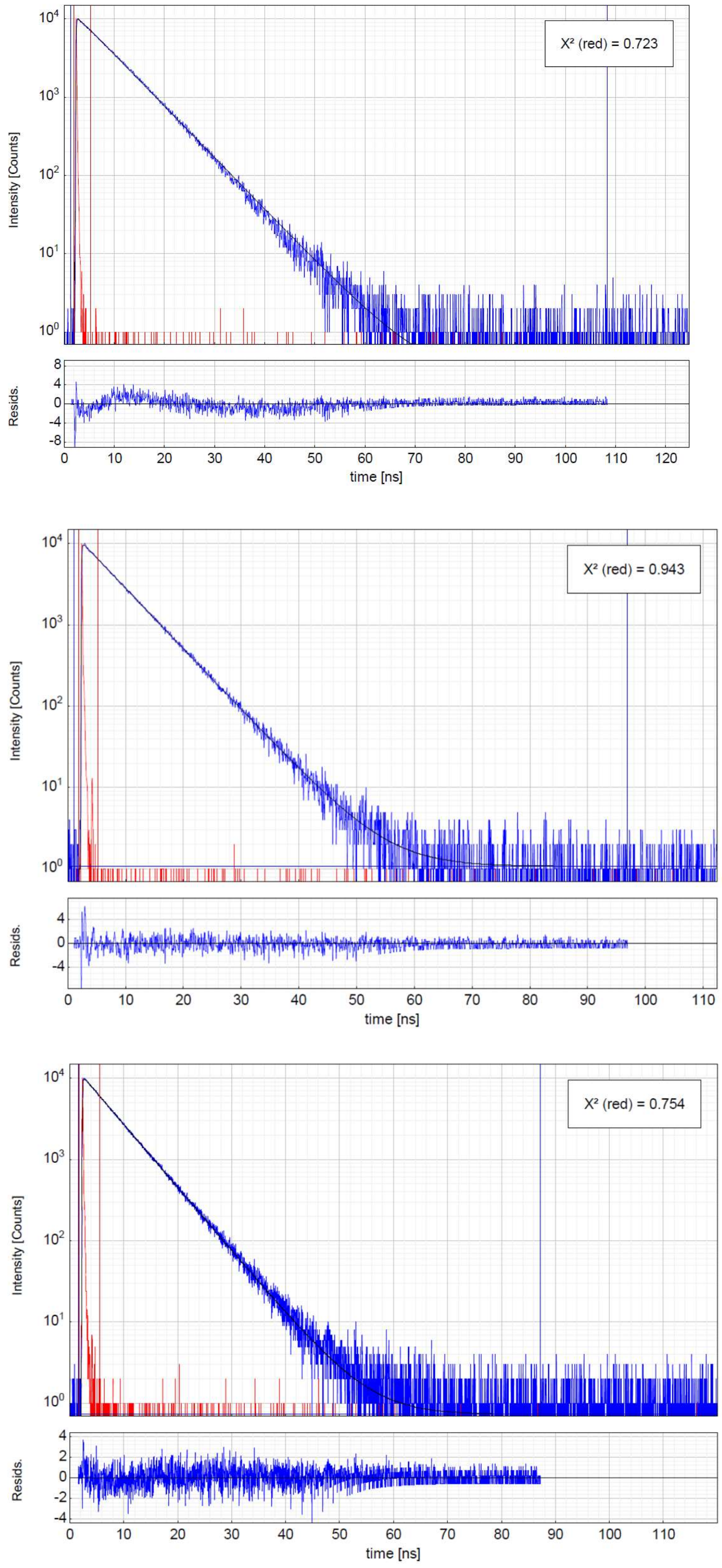

Time-resolved photoluminescence decay (including the instrument response function in red and the residuals of the fit) of compound **19** in CH_3CN (top), CH_2Cl_2/CH_3OH at rt (center) and CH_2Cl_2/CH_3OH at 77 K (bottom).

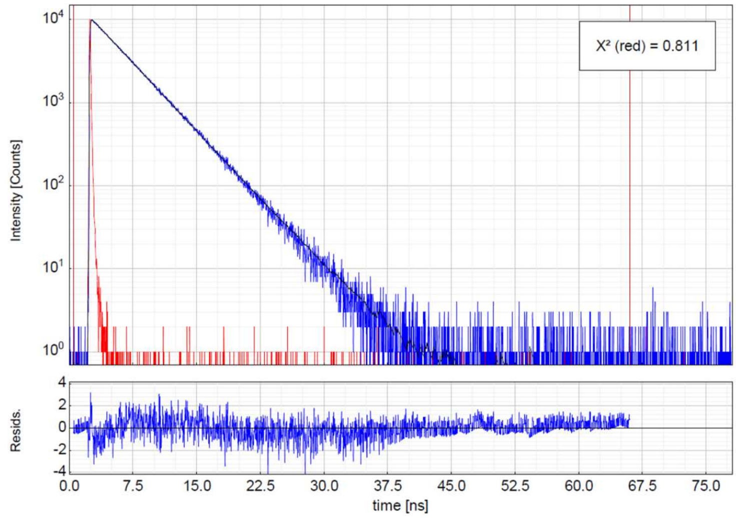

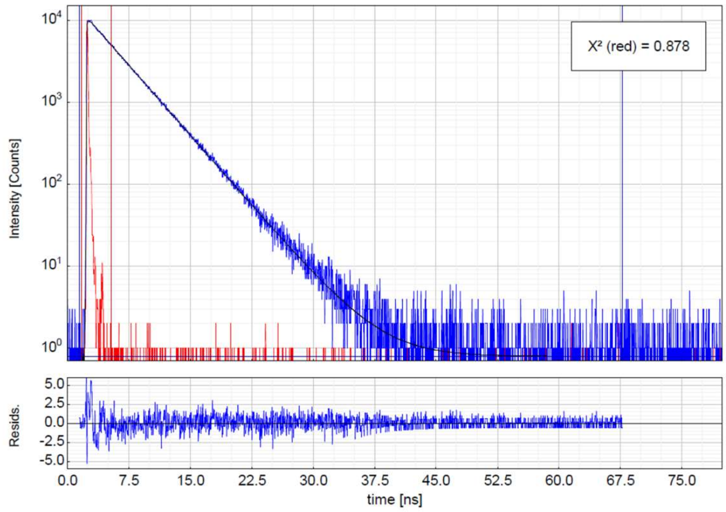

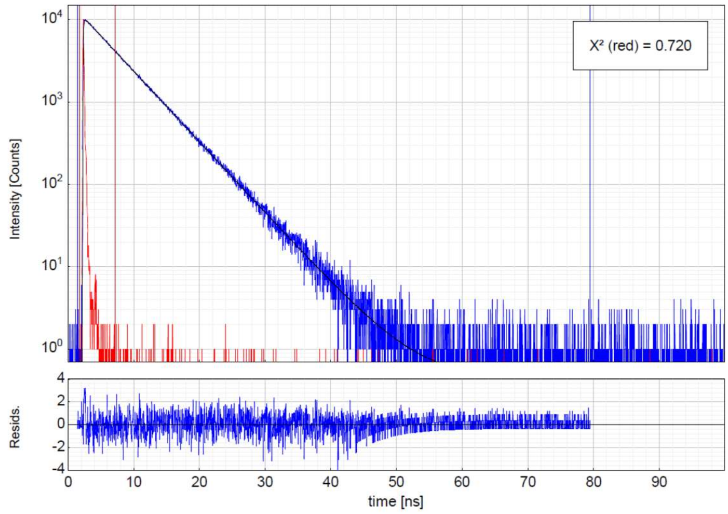

Time-resolved photoluminescence decay (including the instrument response function in red and the residuals of the fit) of compound **20** in CH_3CN (top), CH_2Cl_2/CH_3OH at rt (center) and CH_2Cl_2/CH_3OH at 77 K (bottom).

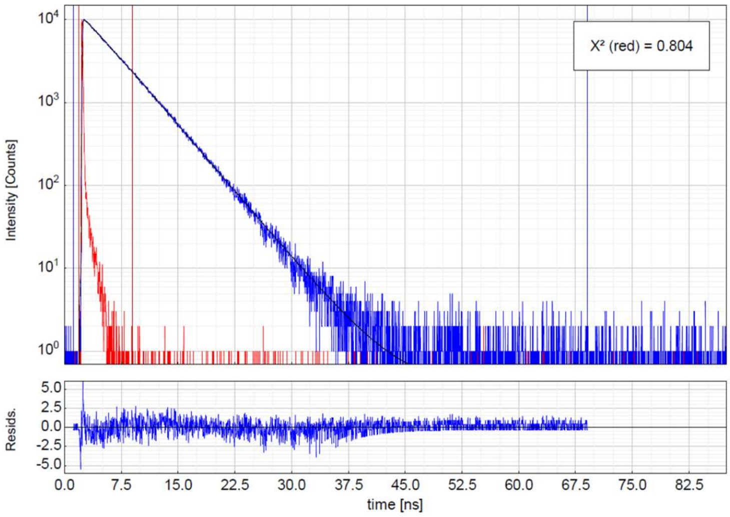

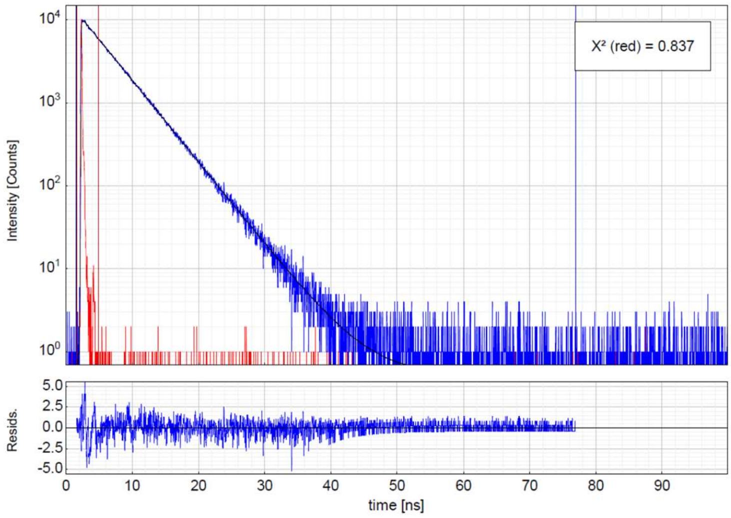

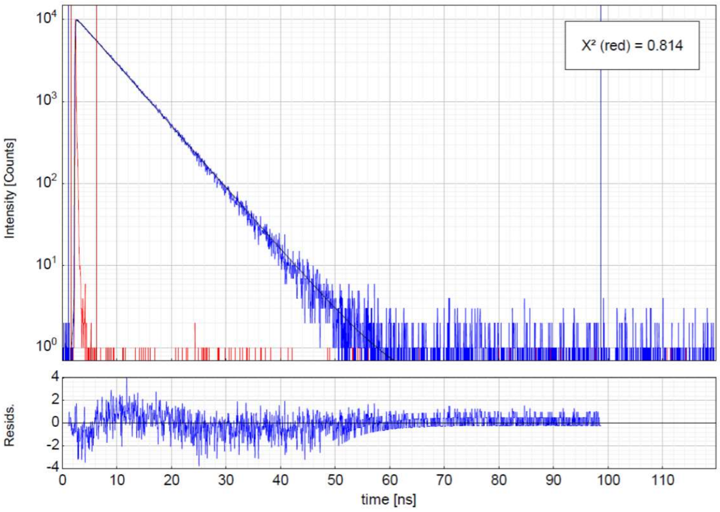

10. References

[1] B. A. Kamino, J. Castrucci, T. P. Bender, *Silicon* **2011**, *3*, 125.

[2] C. P. Konken, G. Haufe, K. Brömmel, B. Wünsch, M. Schäfers, S. Wagner, V. Hugenberg, *Dyes Pigm.* **2018**, *158*, 88.

[3] X. Zhang, Y. Xiao, X. Qian, *Org. lett.* **2008**, *10*, 29.

[4] C.-H. Lee, R. MacKinnon, *Science (New York, N.Y.)* **2018**, *360*, 508.

[5] Molecular Operating Environment (MOE) 2012.10; Chemical Computing Group Inc., 1010 Sherbooke St. West, Suite #910, Montreal, QC, Canada, H3A 2R7, 2012.

[6] W. D. Cornell, P. Cieplak, C. I. Bayly, I. R. Gould, K. M. Merz, D. M. Ferguson, D. C. Spellmeyer, T. Fox, J. W. Caldwell, P. A. Kollman, *J. Am. Chem. Soc.* **1995**, *117*, 5179.

[7] A. Jakalian, B. L. Bush, D. B. Jack, C. I. Bayly, *J. Comput. Chem.* **2000**, *21*, 132.

[8] H. M. Nguyen, V. Singh, B. Pressly, D. P. Jenkins, H. Wulff, V. Yarov-Yarovoy, *Mol. Pharmacol.* **2017**, *91*, 392.
